# A Case-Based, Longitudinal Curriculum in Pediatric Behavioral and Mental Health

**DOI:** 10.15766/mep_2374-8265.11400

**Published:** 2024-04-29

**Authors:** Michelle E. Kiger, Lauryn Fowler, Maggie Eviston, Amanda Cook, Jason Forbush, Suzie Nelson, William Clark, Caitlin Hammond, Kara Knickerbocker, Elizabeth Gawrys-Strand, Daniel Schulteis, John Duby

**Affiliations:** 1 Associate Professor, Department of Pediatrics, Uniformed Services University of the Health Sciences F. Edward Hébert School of Medicine; Military Pediatric Residency Program Director, Wright-Patterson Medical Center; 2 Assistant Professor, Department of Pediatrics, Uniformed Services University of the Health Sciences F. Edward Hébert School of Medicine; 3 Third-Year Pediatric Resident, Dayton Children's Hospital/Wright-Patterson Medical Center; 4 Assistant Professor, Department of Psychiatry, Wright State University Boonshoft School of Medicine; 5 Assistant Professor, Department of Pediatrics, Medical College of Wisconsin; 6 Professor and Chair, Department of Pediatrics, Wright State University Boonshoft School of Medicine

**Keywords:** Child & Adolescent Psychiatry, Entrustable Professional Activities, Pediatric Behavioral Health, Pediatric Mental Health, Case-Based Learning, Competency-Based Medical Education (Competencies, Milestones, EPAs), Pediatrics, Psychiatry, Integrated Behavioral Health

## Abstract

**Introduction:**

Pediatric behavioral and mental health (BMH) disorders are increasingly common, but most pediatricians feel inadequately trained to manage them. We implemented a case-based, longitudinal curriculum in BMH within a pediatric residency program to prepare trainees to diagnose and manage these conditions.

**Methods:**

The pediatric residency program at Wright State University/Wright-Patterson Medical Center implemented a new BMH curriculum in 2020–2021. The curriculum consisted of five simulated cases involving depression, anxiety, attention deficit disorder with hyperactivity (ADHD), developmental delays, behavioral concerns, and autism. To reflect follow-up within a continuity clinic, cases included initial encounters and multiple follow-up visits. Faculty facilitators led residents in monthly small-group meetings over the academic year, with each session consisting of two to three simulated patient encounters. Residents completed pre-post surveys regarding their confidence in diagnosing and managing BMH conditions and pre- and posttests to evaluate the impact of the curriculum on knowledge gains.

**Results:**

All 47 pediatric residents participated in the curriculum; 38 (81%) completed pre-post surveys. Upon completion of the curriculum, residents reported significantly increased confidence in managing ADHD, treating depression, creating safety plans for suicidality, recognizing autism, and counseling patients and families on special education services. Knowledge-based pre- and posttests completed by 25 residents (53%) also demonstrated significant improvement (*M* = 92.4, *SD* = 10.9, pre vs. *M* = 99.3, *SD* = 6.6, post, *p* = .009).

**Discussion:**

This case-based, longitudinal curriculum in pediatric BMH simulating patient continuity improved residents’ confidence and knowledge in diagnosing and managing common BMH conditions.

## Educational Objectives

After completing this curriculum, learners will be able to:

1. Diagnose common pediatric behavioral and mental health conditions, including depression, anxiety, attention deficit disorder with hyperactivity, disruptive and aggressive behavior, learning difficulties/developmental delays, substance use, social-emotional issues, and autism.
2. Provide behavioral and, when indicated, medication management for common pediatric behavioral and mental health conditions, including initial treatments and management of follow-up concerns and complications.
3. Counsel families of children with behavioral and mental health concerns on accessing community- and school-based supports.

## Introduction

Behavioral and mental health (BMH) disorders are increasingly common in pediatrics, affecting an estimated 20% of children,^1,2^ and the COVID-19 pandemic worsened this already concerning trend through its negative impact on pediatric mental health.^3–5^ Given the high demand for BMH services and a shortage of mental health specialists, general pediatricians are often expected to diagnose and manage patients with these concerns and are the only source of care for over one-third of them.^6^ However, despite numerous calls to improve BMH training within pediatric residency programs,^7–9^ the requirement from the Accreditation Council for Graduate Medical Education (ACGME) that pediatric residency curricula include a developmental and behavioral pediatrics rotation,^10^ and the American Board of Pediatrics (ABP) creating a specific Entrustable Professional Activity (EPA) related to BMH,^11^ a majority of pediatricians do not feel adequately trained or equipped to care for these patients.^12–15^

The American Academy of Pediatrics’ Section on Pediatric Trainees has forwarded three recommendations for how to address this gap in knowledge and training regarding BMH: (1) participating in child and adolescent psychiatry rotations; (2) harnessing training opportunities within existing rotations, such as working with mental health crisis response teams within an emergency department rotation; and (3) incorporating structured didactic education in these topics.^15,16^ This report specifically recommends that any curricula developed be shared on interactive platforms. Several online lectures or modules on pediatric BMH topics have been published online,^17–20^ and specifically in *MedEdPORTAL,* a longitudinal curriculum in pediatric BMH has been described within a continuity clinic with integrated mental health care capabilities,^20^ in addition to specific modules on preschool behavioral concerns^18^ and adolescent depression.^19^

These curricula provide an important starting point for equipping pediatric residents in common BMH topics encountered in outpatient practice. However, none are specifically grounded in all elements of the ABP's EPA 9, which outlines expectations for the scope of activities that a pediatric resident should be entrusted to perform within the realm of BMH. Specifically, even the most comprehensive curriculum published to date^18^ does not address autism spectrum disorder, teenage depression, suicidality, substance use, or details of school-based interventions such as individualized education plans/504 plans.

Therefore, we created a curriculum in pediatric BMH that addresses these gaps, including covering all domains of the ABP EPA 9. Furthermore, while some of the prior curricula incorporate case-based learning in some areas, our curriculum is entirely case-based and designed for implementation in small groups to maximize resident engagement and active learning. Our curriculum also delves into greater depth regarding medication management and engages learners to make comprehensive diagnostic and management decisions for these common conditions in significant detail from the point of diagnosis to multiple follow-up visits, including how to troubleshoot common difficulties in management. We investigated the effect of this curriculum on resident comfort in caring for BMH concerns and knowledge gains in these domains.

## Methods

### Population and Setting

In the 2020–2021 academic year, the pediatric residency program at Wright State University/Wright-Patterson Medical Center (WSU/WPMC) implemented a curriculum in BMH. The WSU/WPMC residency program was an integrated military-civilian program with 47 residents, approximately half military and half civilian, and all residents participated in the same rotations and academic curriculum. Prior to implementation of the new curriculum, residents participated in a lecture-based series on common BMH topics toward the beginning of each academic year, but this was replaced by the new curriculum. The lectures covered basic diagnostic criteria and first-line management of anxiety, depression, attention deficit disorder with hyperactivity (ADHD), and autism. Residents from our program participated in outpatient clinic in two locations: (1) Dayton Children's Pediatrics clinic connected to Dayton Children's Hospital, serving a racially and ethnically diverse patient population primarily of low income and publicly insured, and (2) Wright-Patterson Medical Center Pediatric Clinic, serving dependents of both active-duty military and retired service members known to face unique BMH challenges connected to stressors such as parental deployments and frequent moves.^21^

### Curriculum Design

A multidisciplinary group of faculty including general pediatricians, developmental and behavioral pediatricians, child psychologists, and child and adolescent psychiatrists collaborated to create a longitudinal curriculum in BMH. To simulate cases seen longitudinally in a pediatric continuity clinic, we created five cases, each with initial consultations and multiple follow-up visits, as well as corresponding faculty guides and resource handouts:

1. Teenager with depression (Appendices A–D)
2. Young adolescent with anxiety (Appendices E–G)
3. School-age child with ADHD (Appendices H–J)
4. Young child with developmental delays (Appendices K–N)
5. Young child with behavioral concerns and concern for autism (Appendices O–Q)

**Figure 1.**
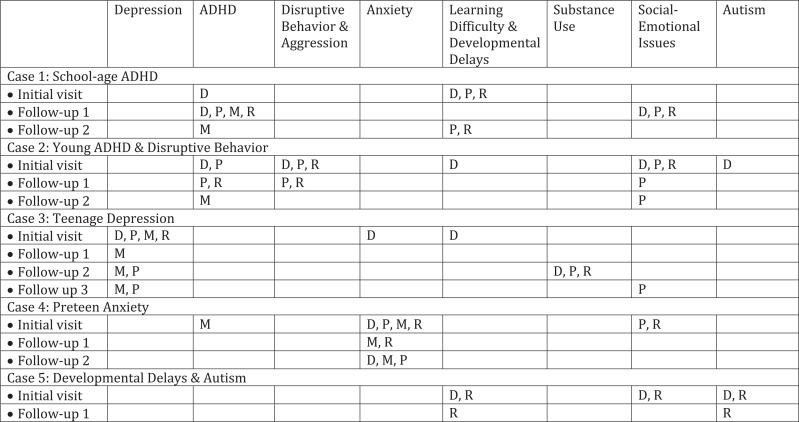
Behavioral and mental health curriculum map linked to Entrustable Professional Activity (EPA) 9. We designed the curriculum to align with the American Board of Pediatrics’ EPA 9 to ensure that each element was contained within the curriculum. Abbreviations: ADHD, attention deficit disorder with hyperactivity; D, diagnosis; M, medication treatment; P, psychosocial treatment; R, referring/comanaging care.

We designed the content of the cases to mirror the ABP's EPA 9, which covered assessing and managing depression, anxiety, ADHD, disruptive and aggressive behavior, learning difficulties/developmental delays, substance use, social-emotional issues, and autism. To operationalize the EPA, we created a curriculum map to guide the content discussed in each case (Figure 1). For each topic, we ensured cases covered diagnosis, psychosocial treatments, and referrals/comanagement, and for the subset of topics in which medication treatment was also recommended by the EPA (specifically, depression, anxiety, and ADHD), cases also addressed medication management.

### Curriculum Implementation

**Figure 2.**
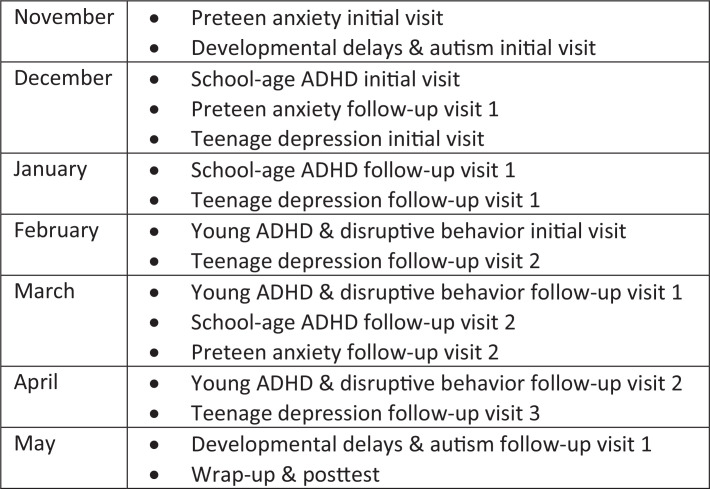
Curriculum implementation schedule. Each 60- to 90-minute session included two to three cases held across the academic year during protected educational half-days. Abbreviation: ADHD, attention deficit disorder with hyperactivity.

Residents from all years of training met monthly in small groups during protected academic half-days with faculty mentors from general pediatrics, developmental and behavioral pediatrics, child and adolescent psychiatry, and psychology to work through the cases over the course of an academic year. Each 60- to 90-minute session consisted of combinations of two to three simulated patient encounters (Figure 2), with the faculty mentor acting as the patient and/or patient family member. Due to the COVID-19 pandemic, we conducted small groups in a hybrid model, with some held in person and some on a video platform (based on current COVID-19 restriction levels at the time of each case and resident clinical rotation assignments).

Resident small groups ranged from eight to 15 residents per session, depending on the number of residents able to attend that session (based on call schedules) and the number of faculty facilitators available. Facilitators were asked to spend approximately 30–40 minutes for each initial encounter and 15–20 minutes for each follow-up visit scheduled during that day's session. Any remaining time was allotted for additional resident questions or discussion following the cases. Since the sessions were entirely paper and discussion based, the curriculum implementation required no specialized audio or visual needs aside from using a secure videoconferencing system to allow residents to join virtually when dictated by local guidelines regarding in-person meeting limitations. In the case of virtual or hybrid sessions, case materials were emailed to residents at the time of the session. Sessions were held in conference rooms typically used for resident didactics.

In each session, the faculty mentor used the faculty guides (Appendices B, F, I, L, P) to help them play the role of the patient and/or patient family member, and residents worked collaboratively to answer questions and devise diagnostic and treatment plans. For follow-up cases, faculty began each case by giving a summary of the prior encounter(s) to provide continuity, which appeared at the top of each resident and faculty guide. Each resident was given paper copies of the corresponding case handout without answers (Appendices A, E, H, K, O) and resources/summary handout (Appendices D, G, J, N, Q) that they could keep and annotate throughout the sessions. Faculty were expected to have thoroughly reviewed cases and facilitator guides ahead of time, but residents were not expected to have completed any preparatory work prior to the sessions. For sessions in which screening tools were used as part of the patient encounter (i.e., SCARED questionnaire for anxiety/Appendix C and Vanderbilt screeners for ADHD/Appendix M), residents were given marked-up copies of the screener and expected to score and interpret the forms as part of the case.

The curriculum was conducted over the course of the academic year and continues to be repeated and updated annually based on resident and faculty feedback, but the core material has not significantly changed. While we held the cases over seven sessions (Figure 2), other programs could consolidate the sessions to run over fewer days or cover fewer cases per session and spread them over a longer period of time. We did time the schedule to try to make the proposed follow-up plan for each patient (e.g., following up with a patient in 1–2 months after making a change to the dosing of a selective serotonin reuptake inhibitor [SSRI]) roughly correlate with when the corresponding follow-up session would be held in real time. The files were stored electronically on a shared drive, which enabled us to ensure links and references were updated as needed each year.

### Curriculum Evaluation

To assess the impact of the curriculum, residents completed a 30-item survey consisting of 5-point rating-scale questions (1 = *not at all confident,* 5 = *very confident*) on their confidence in diagnosing and treating ADHD, anxiety, sleep disorders, suicidality, developmental delays, autism, and special education services before and after the implementation of the curriculum (Appendix R). Additionally, residents completed a 125-point, knowledge-based pre- and posttest covering these topics before and after participating in the curriculum, with test questions also mapped to EPA 9 (Appendix S). We developed the surveys and tests collaboratively based on content from EPA 9 and then provided the survey to two outside faculty members for additional expert feedback on clarity and content to build validity evidence. Pre- and postimplementation survey and test scores were compared using two-tailed *t* tests, with an alpha value of .05 considered statistically significant.

This project was determined to be exempt by the Institutional Review Board of Wright-Patterson Medical Center.

## Results

All 47 residents participated in the curriculum. Attendance at each session averaged around 80% due to some residents being on night shifts, postcall, or on vacation, but residents who were unable to attend in person were able to access case materials electronically. Thirty-eight residents (81%) completed the pre-post surveys (Table). After completing the curriculum, residents reported significantly improved confidence in diagnosing and treating ADHD (*M*s = 3.7 vs. 4.2, *t* = 2.45, *p* = .02, diagnosis; *M*s = 3.6 vs. 4.3, *t* = 2.54, *p* = .02, treatment), treating depression (*M*s = 3.9 vs. 4.4, *t* = 2.22, *p* = .03), developing safety plans for suicidality (*M*s = 2.7 vs. 3.4, *t* = 2.53, *p* = .01), recognizing autism (*M*s = 3.9 vs. 4.4, *t* = 2.35, *p* = .02), and counseling families on advocating for special education services (*M*s = 2.6 vs. 3.8, *t* = 4.44, *p* < .001). Resident confidence in diagnosing depression and anxiety was relatively high and did not show significant differences pre versus post. Feedback sessions with residents also reported high satisfaction with the format of the small groups, the simulated continuity of care, and the level of detail covered in the cases.

**Table.**
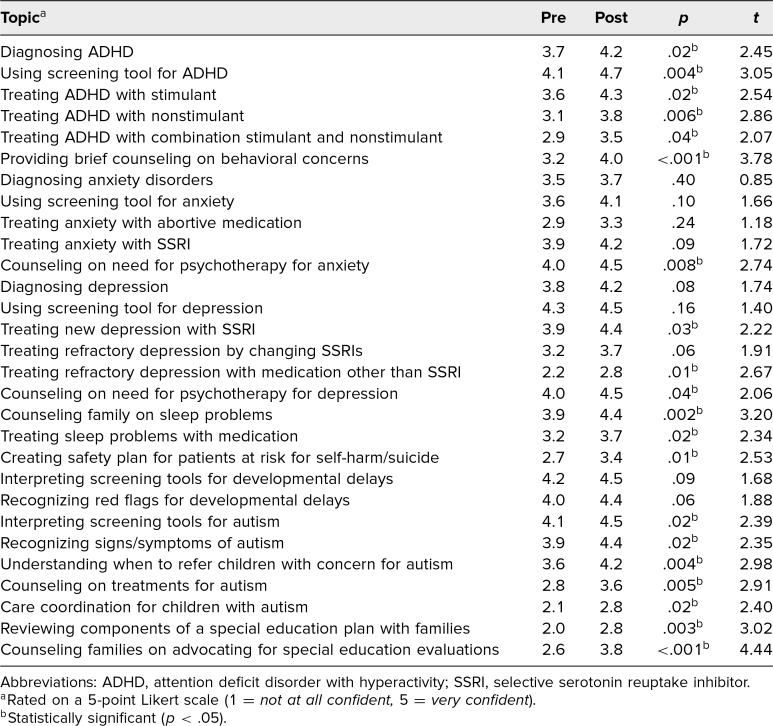
Resident Confidence Pre- Versus Postparticipation in the Curriculum

Twenty-five residents (53%) completed a comprehensive 125-point knowledge pre- and posttest. Resident knowledge scores significantly improved after completing the curriculum (*M* = 92.4, *SD* = 10.9, pre vs. *M* = 99.3, *SD* = 6.6, post, *t*(24) = −2.96, *p* = .009, Cohen's *d* = 0.76). In analyzing scores by class year, we found improvements across all three years, although the intern class demonstrated the largest improvements (*M* = 86.9, *SD* = 13.0, pre vs. *M* = 98.1, *SD* = 7.5, post, *t*(11) = 2.75, Cohen's *d* = 1.05). The test subsections on which residents scored lowest were the details of pharmacologic management for anxiety/depression and ADHD, particularly when moving past first-line SSRI and stimulant therapies, respectively, and questions related to special education services.

## Discussion

To address the gap in pediatric BMH training, we successfully implemented a case-based, longitudinal curriculum in pediatric BMH. The curriculum was feasible to implement on both in-person and virtual platforms and improved resident confidence and knowledge related to treatment of common pediatric BMH conditions. One of the primary concerns amongst residents in our program about their BMH training prior to this curricular implementation was that the elements of BMH care they personally saw in continuity clinics were sporadic. For example, many had provided refills of ADHD medications but had never made the initial diagnosis or been responsible for choosing an initial medication; others had diagnosed depression and started an SSRI but did not have the patient continuity to follow such patients and adjust medications as indicated. The longitudinal format of the curriculum and the cases being tied to the EPA-based curriculum map ensured that all residents had exposure to, at minimum, a simulated setting in which to diagnose and make ongoing management decisions for these common conditions. The case-based nature of the curriculum also enabled active learning throughout all its portions. Having a multidisciplinary team of faculty who developed and facilitated the cases was key to implementation success, so we highly recommend obtaining buy-in from key stakeholders up front when adopting such a curriculum so that a diverse group of faculty can actively participate. When possible based on faculty availability, we tried to have one general pediatrician and one subspecialty faculty member (e.g., child/adolescent psychiatrist or behavioral and developmental pediatrician) facilitate each small group in order to provide the general pediatric perspective on how to manage such patients in the context of an outpatient clinic, as well as more subspecialized knowledge when needed.

Given the severity of the pediatric mental health crisis and the need to improve training for pediatric trainees in BMH topics, we believe that a variety of curricula in pediatric BMH are urgently needed so that programs can decide which best meets the needs of their learners. These curricula will become even more relevant as pediatric residency programs are required to include a mental health rotation under proposed revisions to the ACGME guidelines and must determine how to prepare residents for and maximize the educational benefits of such rotations.^22^ Particularly considering the increased demand for pediatric mental health care due to the COVID-19 pandemic,^3–5^ as well as increasing recognition that the burden of such conditions falls disproportionately on racial and sexual minority youth who often have greater difficulty accessing care,^23,24^ pediatric residents must be trained to provide comprehensive BMH care to their patients and to practice to the fullest scope of their abilities. This curriculum can serve as a stand-alone resource for more advanced learners to expand their scope of practice or as a follow-on or complementary curriculum to others such as those by Meyers and colleagues^20^ that introduce these topics. Additionally, in the context of the shift toward competency-based medical education, aligning curricula with EPAs, particularly when implemented in an active, participatory format, can provide valuable information for faculty looking for ways to assess competence among pediatric trainees. For example, cases from this curriculum could be incorporated into observed structured clinical encounters or other trainee evaluations to inform entrustment decisions.

Our curriculum is limited in that it was conducted at a single pediatric residency program. Also, it has been designed with the expectation that residents have some fundamental background knowledge of pediatric BMH conditions and focuses on application of these principles to case-based scenarios. For residents without a foundation in such conditions, or if this curriculum were implemented with medical students, some might require additional preparatory work or background reading to optimally interact in the small groups. Additionally, while our program conducted the cases in 60- to 90-minute sessions, other programs may choose to allot more time if needed for additional didactic instruction or discussion. Furthermore, we had a lower response rate to our posttest (53%) and were unable to separate differences in learning gains between hybrid and in-person sessions. It is also possible that some of the pre-post survey and test score improvements were the result of natural learning and progression across the academic year (i.e., through direct patient care and/or independent study) and not solely due to our curriculum. However, based on resident feedback, we are confident that this curriculum has had a significant impact on resident confidence and competence in caring for BMH conditions.

Future studies should examine the effect of such curricula on changes in practice patterns and other patient-level outcomes, such as access to BMH care or rates of subspecialty referrals, amongst pediatric residents and recent graduates. We are currently planning a follow-on study in which we will investigate practice patterns related to in-office brief counseling and medication prescribing for BMH conditions amongst our residency graduates who have completed this curriculum. Additionally, for our trainees in their final year of residency who have twice participated in these cases, we are now incorporating a peer teaching track in which they can serve as cofacilitators alongside faculty for these cases, and we plan to investigate the impact of this change on the experiences of residents progressing through the curriculum.

Pediatric BMH is increasingly situated within the expected scope of practice of general pediatricians, so pediatric residency programs must prepare their trainees to be competent and confident in treating such conditions. This case-based, longitudinal curriculum significantly improved resident confidence and knowledge in diagnosis and management of BMH conditions covered by the ABP's EPA 9.

## Appendices


Preteen Anxiety Case - Residents.docxPreteen Anxiety Case - Faculty Guide.docxPreteen Anxiety Case - SCARED Forms.pdfAnxiety Resources Handout.docxASD Delays Case - Residents.docxASD Delays Case - Faculty Guide.docxAutism Summary Handout and Resources.docxDepression Case - Residents.docxDepression Case - Faculty Guide.docxDepression Resources Handout.docxSchool-age ADHD Case - Residents.docxSchool-age ADHD Case - Faculty Guide.docxSchool-age ADHD Case - Vanderbilts.pdfADHD Handout.docxYoung ADHD and Behavior Case - Residents.docxYoung ADHD and Behavior Case - Faculty Guide.docxParenting Handout and Resource Sheet.docxBehavioral and Mental Health Curriculum Survey.docxBehavioral and Mental Health Pre-Post Test.docx
*All appendices are peer reviewed as integral parts of the Original Publication.*

## References

[1] American Academy of Child and Adolescent Psychiatry Committee on Health Care Access and Economics Task Force on Mental Health. Improving mental health services in primary care: reducing administrative and financial barriers to access and collaboration. Pediatrics. 2009;123(4):1248–1251. 10.1542/peds.2009-0048 19336386

[2] Whitney DG, Peterson MD. US national and state-level prevalence of mental health disorders and disparities of mental health care use in children. JAMA Pediatr. 2019;173(4):389–391. 10.1001/jamapediatrics.2018.5399 30742204 PMC6450272

[3] Golberstein E, Wen H, Miller BF. Coronavirus disease 2019 (COVID-19) and mental health for children and adolescents. JAMA Pediatr. 2020;174(9):819–820. 10.1001/jamapediatrics.2020.1456 32286618

[4] Ibeziako P, Kaufman K, Scheer KN, Sideridis G. Pediatric mental health presentations and boarding: first year of the COVID-19 pandemic. Hosp Pedatr. 2022;12(9):751–760. 10.1542/hpeds.2022-006555 35578918

[5] Gould JB, Walter HJ, Bromberg J, Correa ET, Hatoun J, Vernacchio L. Impact of the coronavirus disease 2019 pandemic on mental health visits in pediatric primary care. Pediatrics. 2022;150(6):e2022057176. 10.1542/peds.2022-057176 36330753

[6] Anderson LE, Chen ML, Perrin JM, Van Cleave J. Outpatient visits and medication prescribing for US children with mental health conditions. Pediatrics. 2015;136(5):e1178–e1185. 10.1542/peds.2015-0807 26459647 PMC4621795

[7] McMillan JA, Land M Jr, Leslie LK. Pediatric residency education and the behavioral and mental health crisis: a call to action. Pediatrics. 2017;139(1):e20162141. 10.1542/peds.2016-2141 28011943

[8] Haggerty RJ. The changing role of the pediatrician in child health care. Am J Dis Child. 1974;127(4):545–549. 10.1001/archpedi.1974.02110230091016 4821319

[9] Leslie L, Rappo P, Abelson H, et al. Final report of the FOPE II Pediatric Generalists of the Future Workgroup. Pediatrics. 2000;106(suppl E1):1199–1223. 10.1542/peds.106.SE1.1199 11073552

[10] Accreditation Council for Graduate Medical Education. ACGME Program Requirements for Graduate Medical Education in Pediatrics. Accreditation Council for Graduate Medical Education; 2022. Accessed March 25, 2024. https://www.acgme.org/globalassets/pfassets/programrequirements/320_pediatrics_2023.pdf

[11] American Board of Pediatrics. Entrustable Professional Activities: EPA 9 for General Pediatrics. American Board of Pediatrics; 2021. Accessed March 25, 2024. https://www.abp.org/sites/abp/files/pdf/gen_peds_epa_9.pdf

[12] Stein REK, Storfer-Isser A, Kerker BD, et al. Beyond ADHD: how well are we doing? Acad Pediatr. 2016;16(2):115–121. 10.1016/j.acap.2015.08.012 26514649 PMC5560870

[13] Horwitz SM, Storfer-Isser A, Kerker BD, et al. Barriers to the identification and management of psychosocial problems: changes from 2004 to 2013. Acad Pediatr. 2015;15(6):613–620. 10.1016/j.acap.2015.08.006 26409303 PMC4639452

[14] Horwitz SM, Kelleher KJ, Stein REK, et al. Barriers to the identification and management of psychosocial issues in children and maternal depression. Pediatrics. 2007;119(1):e208–e218. 10.1542/peds.2005-1997 17200245

[15] Freed GL, Dunham KM, Switalski KE, Jones MD Jr, McGuinness GA; Research Advisory Committee of the American Board of Pediatrics. Recently trained general pediatricians: perspectives on residency training and scope of practice. Pediatrics. 2009;123(suppl 1):S38–S43. 10.1542/peds.2008-1578J 19088244

[16] Raval GR, Doupnik SK. Closing the gap: improving access to mental health care through enhanced training in residency. Pediatrics. 2017;139(1):e20163181. 10.1542/peds.2016-3181 27940515 PMC5192092

[17] Child Health Education Center: building mental wellness abnormal development screening. Ohio Chapter, American Academy of Pediatrics. 2019. Accessed March 25, 2024. https://ohioaap.org/child-health-education-center/building-mental-wellness-abnormal-developmental-screening/

[18] Axelrad ME, Chapman S. The Brief Behavioral Intervention for preschoolers with disruptive behaviors: a clinical program guide for clinicians. MedEdPORTAL. 2016;12:10376. 10.15766/mep_2374-8265.10376

[19] Stanley A, Chelvakumar G, Cody P, et al. Resident training curriculum in adolescent depression and suicide screening. MedEdPORTAL. 2016;12:10361. 10.15766/mep_2374-8265.10361

[20] Meyers N, Maletz B, Berger-Jenkins E, et al. Mental health in the medical home: a longitudinal curriculum for pediatric residents on behavioral and mental health care. MedEdPORTAL. 2022;18:11270. 10.15766/mep_2374-8265.11270 35990196 PMC9343532

[21] Huebner CR; AAP Section on Uniformed Services; AAP Committee on Psychosocial Aspects of Child and Family Health. Health and mental health needs of children in US military families. Pediatrics. 2019;143(1):e20183258. 10.1542/peds.2018-3258 30584059

[22] Accreditation Council for Graduate Medical Education. ACGME Program Requirements for Graduate Medical Education in Pediatrics: Summary and Impact of Major Requirement Revisions. Accreditation Council for Graduate Medical Education; 2023. Accessed March 25, 2024. https://www.acgme.org/globalassets/pfassets/reviewandcomment/320_pediatrics_impact-022023.pdf

[23] Patcher LM, Coll CG. Racism and child health: a review of the literature and future directions. J Dev Behav Pediatr. 2009;30(3):255–263. 10.1097/DBP.0b013e3181a7ed5a 19525720 PMC2794434

[24] Coulter RWS, Egan JE, Kinsky S, et al. Mental health, drug, and violence interventions for sexual/gender minorities: a systematic review. Pediatrics. 2019;144(3):e20183367. 10.1542/peds.2018-3367 31427462 PMC6855817

