# Supplementary material for: A Case-Based, Longitudinal Curriculum in Pediatric Behavioral and Mental Health
Source: MedEdPORTAL. 2024 Apr 29;20:11400. doi: 10.15766/mep_2374-8265.11400 (PMC11056487; doi:10.15766/mep_2374-8265.11400)
Supplement: Supplementary file 1 — Preteen Anxiety Case - Residents.docxPreteen Anxiety Case - Faculty Guide.docxPreteen Anxiety Case - SCARED Forms.pdfAnxiety Resources Handout.docxASD Delays Case - Residents.docxASD Delays Case - Faculty Guide.docxAutism Summary Handout and Resources.docxDepression Case - Residents.docxDepression Case - Faculty Guide.docxDepression Resources Handout.docxSchool-age ADHD Case - Residents.docxSchool-age ADHD Case - Faculty Guide.docxSchool-age ADHD Case - Vanderbilts.pdfADHD Handout.docxYoung ADHD and Behavior Case - Residents.docxYoung ADHD and Behavior Case - Faculty Guide.docxParenting Handout and Resource Sheet.docxBehavioral and Mental Health Curriculum Survey.docxBehavioral and Mental Health Pre-Post Test.docx [file mep_2374-8265.11400-s001.zip › Q. Parenting Handout and Resource Sheet.docx]

**Resources on Psychoeducation, Parenting Tips, and School**

**Tips you can provide in the moment that are true for most parenting, but especially true for ADHD:**

- Set clear, consistent expectations, directions, and limits
- Set goals with agreed upon rewards (write it out)
- Visual reminders are helpful
  - Written and/or pictures, depending on developmental level
  - Use to track progress toward goals
- Praise successes, no matter how small. Positive reinforcement techniques always work best!
- Set aside a special time for positive interaction
  - a place your child will thrive: a walk, playing legos, etc.
- Stay calm
  - Avoids “high expressed emotion” which is often harmful
  - Sets the example
  - Also avoids accidental reinforcement through attention
- May need to help make the connection with behavior and consequences (even natural consequences), help to learn from mistakes
- Pick your battles
  - Ignore behaviors you don’t like (unless UNSAFE)
- Give time to process changes, prep for transitions with countdowns
- Sleep tips: exercise daily, avoid caffeine, stick to a schedule, sleep-enriching environment

**Web-based Resources:**

- <https://CHADD.org>
- Healthychildren.org
  - Search keywords “ADHD” and “ADHD and Schools”
- <https://www.aacap.org/AACAP/Families_Youth/Resource_Centers/AACAP/Families_and_Youth/Resource_Centers/ADHD_Resource_Center/Home.aspx>
- <https://mydoctor.kaiserpermanente.org/ncal/Images/ADHD_A_Guide_for_Parents_English_ADA_tcm75-891010.pdf>
- <https://www.triplep-parenting.com/oh-en/triple-p/> (FREE in Ohio!)

**Book Resources:**

- Parenting with Love and Logic by Foster Cline and Jim Fay NavPress, 2006
- Common Sense Parenting by Ray Burke, Ron Herron, Bridget Barnes, 4^th^ ed 2015
- 1, 2, 3, Magic by Thomas W. Phelan, Child Management Incorporated, 6^th^ ed 2016
- Taking Charge of ADHD by Russell A Barkley, Guilford Press New York, 4^th^ ed 2020
- The Incredible Years by Carolyn Webster-Stratton, 3^rd^ ed 2019
- Parenting That Works by Edward Christophersen and Susan Mortweet, American Psychological Association, 2003
- How to Talk So Kids Will Listen and Listen So Kids Will Talk by Adele Faber and Elaine Mazlish, Scribner, 2012
- Raising Your Spirited Child by Mary Sheedy Kurcinka, William Morrow Paperbacks, 2015
- The Explosive Child by Ross W. Greene, Harper Collins, 4^th^ ed 2021
