# Supplementary material for: A Case-Based, Longitudinal Curriculum in Pediatric Behavioral and Mental Health
Source: MedEdPORTAL. 2024 Apr 29;20:11400. doi: 10.15766/mep_2374-8265.11400 (PMC11056487; doi:10.15766/mep_2374-8265.11400)
Supplement: Supplementary file 1 — Preteen Anxiety Case - Residents.docxPreteen Anxiety Case - Faculty Guide.docxPreteen Anxiety Case - SCARED Forms.pdfAnxiety Resources Handout.docxASD Delays Case - Residents.docxASD Delays Case - Faculty Guide.docxAutism Summary Handout and Resources.docxDepression Case - Residents.docxDepression Case - Faculty Guide.docxDepression Resources Handout.docxSchool-age ADHD Case - Residents.docxSchool-age ADHD Case - Faculty Guide.docxSchool-age ADHD Case - Vanderbilts.pdfADHD Handout.docxYoung ADHD and Behavior Case - Residents.docxYoung ADHD and Behavior Case - Faculty Guide.docxParenting Handout and Resource Sheet.docxBehavioral and Mental Health Curriculum Survey.docxBehavioral and Mental Health Pre-Post Test.docx [file mep_2374-8265.11400-s001.zip › R. Behavioral and Mental Health Curriculum Survey.docx]

**Behavioral and Mental Health Curriculum Pre- and Post-Survey**

1. Please indicate your current status in residency training: □ PGY1 □ PGY2 □ PGY3

For the following questions, please rate your current confidence level with performing the given task in your clinic (for questions involving treatment, assume that the given treatment is clinically indicated):

|  | Not at all  Confident | Somewhat  Unconfident | Neutral | Somewhat  Confident | Very  Confident |
| --- | --- | --- | --- | --- | --- |
| 1. Diagnosing Attention-Deficit Hyperactivity Disorder (ADHD) in a pediatric patient | 1 | 2 | 3 | 4 | 5 |
| 2. Using a screening tool for evaluation of ADHD (e.g., Vanderbilt, ADHD-rating scale) | 1 | 2 | 3 | 4 | 5 |
| 3. Treating ADHD with a stimulant medication | 1 | 2 | 3 | 4 | 5 |
| 4. Treating ADHD with a non-stimulant medication | 1 | 2 | 3 | 4 | 5 |
| 5. Treating ADHD with a combination of stimulant plus non-stimulant medications | 1 | 2 | 3 | 4 | 5 |
| 6. Providing brief counseling to parents on the management of common behavior concerns | 1 | 2 | 3 | 4 | 5 |
| 7. Diagnosing an Anxiety Disorder in a pediatric patient (Anxiety Disorders excluding PTSD) | 1 | 2 | 3 | 4 | 5 |
| 8. Using a screening tool for evaluation of anxiety (e.g. rating scales such as GAD-7, SCARED) | 1 | 2 | 3 | 4 | 5 |
| 9. Treating anxiety with an abortive medication for acute anxiety (i.e. hydroxyzine, propranolol) | 1 | 2 | 3 | 4 | 5 |
| 10. Treating anxiety with a selective serotonin reuptake inhibitor (SSRI) | 1 | 2 | 3 | 4 | 5 |
| 11. Counseling to encourage pursuit of psychotherapy for anxiety (e.g., discussing benefits of relaxation) | 1 | 2 | 3 | 4 | 5 |
| 12. Diagnosing a Depressive Disorder in a pediatric patient | 1 | 2 | 3 | 4 | 5 |
| 13. Using a screening tool for evaluation of depression (e.g., rating scales such as PHQ-9, CDI, Beck Depression Inventory) | 1 | 2 | 3 | 4 | 5 |
| 14. Treating new-onset depression with an initial trial of an SSRI | 1 | 2 | 3 | 4 | 5 |
| 15. Treating refractory depression by changing from one SSRI to another SSRI | 1 | 2 | 3 | 4 | 5 |
| 16. Treating refractory depression with any antidepressant other than SSRIs (e.g. venlafaxine, bupropion, mirtazapine) | 1 | 2 | 3 | 4 | 5 |
| 17. Counseling to encourage pursuit of psychotherapy for depression (e.g., cognitive behavioral therapy) | 1 | 2 | 3 | 4 | 5 |
| 18. Providing educational resources to a family for helping a child with sleep problems (e.g., sleep hygiene) | 1 | 2 | 3 | 4 | 5 |

|  | Not at all  Confident | Somewhat  Unconfident | Neutral | Somewhat  Confident | Very  Confident |
| --- | --- | --- | --- | --- | --- |
| 19. Treating sleep problems with a recommendation for medication (e.g., melatonin, clonidine, trazodone) | 1 | 2 | 3 | 4 | 5 |
| 20. Creating a safety plan for patients at risk for self-harm/suicide | 1 | 2 | 3 | 4 | 5 |
| 21. Interpreting screening tools for developmental delays, such as the Ages and Stages Questionnaire (ASQ) | 1 | 2 | 3 | 4 | 5 |
| 22. Recognizing “red flags” for developmental delay that would prompt further evaluation | 1 | 2 | 3 | 4 | 5 |
| 23. Interpreting screening tools for autism, such as the Modified Checklist for Autism in Toddlers, Revised (M-CHAT-R) | 1 | 2 | 3 | 4 | 5 |
| 24. Recognizing signs/symptoms of autism spectrum disorder | 1 | 2 | 3 | 4 | 5 |
| 25. Understanding when to refer children for further autism spectrum disorder evaluation | 1 | 2 | 3 | 4 | 5 |
| 26. Counseling families on treatment options for autism spectrum disorder | 1 | 2 | 3 | 4 | 5 |
| 27. Coordinating a comprehensive care plan for children with autism spectrum disorder | 1 | 2 | 3 | 4 | 5 |
| 28. Reviewing components of a special education plan (e.g., 504 plan, Individualized Education Plan) with a patient family | 1 | 2 | 3 | 4 | 5 |
| 29. Counseling families to advocate for obtaining/updating special education evaluations for children with special needs | 1 | 2 | 3 | 4 | 5 |
