# Supplementary material for: A Case-Based, Longitudinal Curriculum in Pediatric Behavioral and Mental Health
Source: MedEdPORTAL. 2024 Apr 29;20:11400. doi: 10.15766/mep_2374-8265.11400 (PMC11056487; doi:10.15766/mep_2374-8265.11400)
Supplement: Supplementary file 1 — Preteen Anxiety Case - Residents.docxPreteen Anxiety Case - Faculty Guide.docxPreteen Anxiety Case - SCARED Forms.pdfAnxiety Resources Handout.docxASD Delays Case - Residents.docxASD Delays Case - Faculty Guide.docxAutism Summary Handout and Resources.docxDepression Case - Residents.docxDepression Case - Faculty Guide.docxDepression Resources Handout.docxSchool-age ADHD Case - Residents.docxSchool-age ADHD Case - Faculty Guide.docxSchool-age ADHD Case - Vanderbilts.pdfADHD Handout.docxYoung ADHD and Behavior Case - Residents.docxYoung ADHD and Behavior Case - Faculty Guide.docxParenting Handout and Resource Sheet.docxBehavioral and Mental Health Curriculum Survey.docxBehavioral and Mental Health Pre-Post Test.docx [file mep_2374-8265.11400-s001.zip › D. Anxiety Resources Handout.docx]

**Anxiety Summary Sheet and Resources Handout**

**FDA-Approved Medication Options for Anxiety**


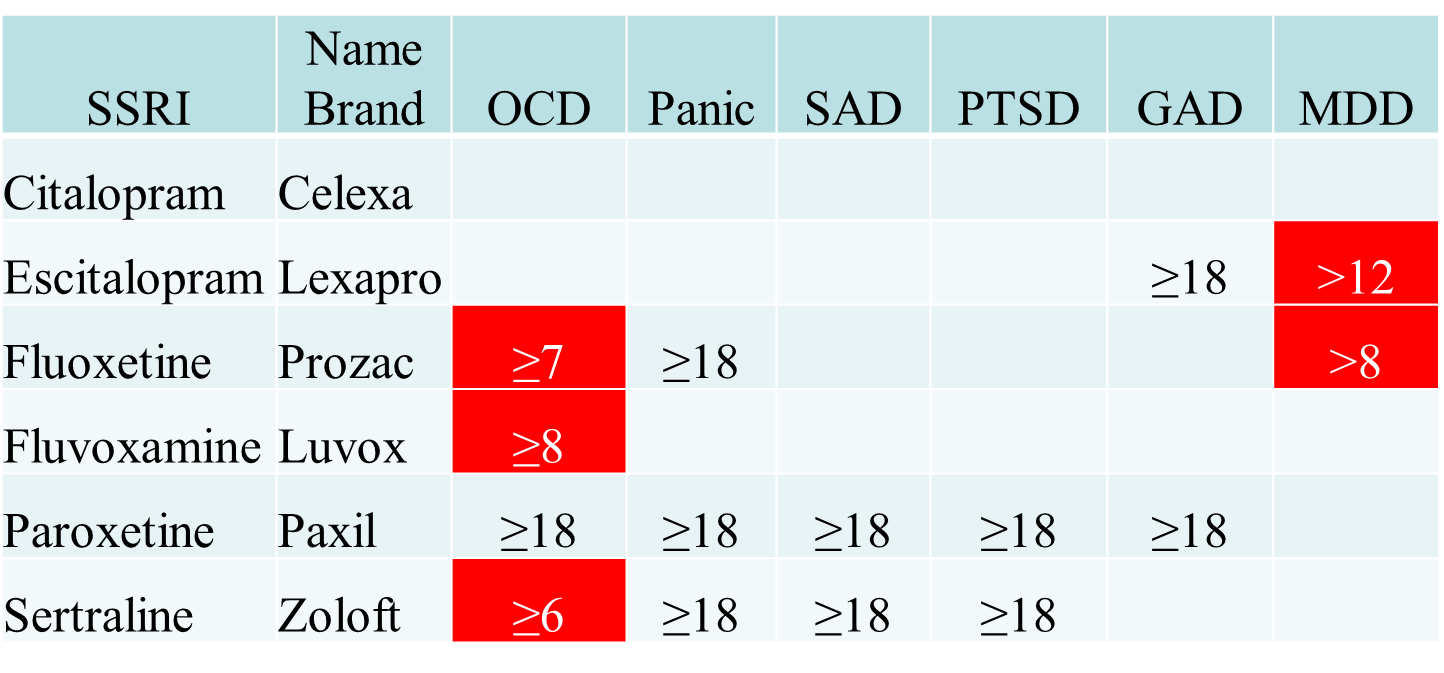


Author Owned

**Medication Dosing Chart**

| **SSRI** | **Starting Dose (mg)** | **Dosing Increments (mg)** | **Minimum Effective Dose (mg)** | **Maximum Dose (mg)** |
| --- | --- | --- | --- | --- |
| Fluoxetine  (Prozac®) | 10 | 10-20 | 20 | 60 |
| Citalopram  (Celexa®) | 10 | 10 | 20 | 40* |
| Fluvoxamine  (Luvox®) | 50 | 50 | 150 | 300 |
| Sertraline  (Zoloft®) | 25 | 12.5-25 | 50 | 200 |
| Escitalopram  (Lexapro®) | 5 | 5 | 10 | 20 |

Author Owned

**Helping Your Child Cope With Anxiety**

**Routines**

Predictable routines lead to fewer surprises and less stress for everyone!

Write them out and be consistent

**Sleep Hygiene**

o Create a regular sleep schedule and stick to it, even when school is out

o Decrease intake of sugar, caffeine, and second hand smoke for 3 hours before bedtime

o Use relaxation activities for 20 minutes before bedtime

o Create a routine of reading, storytelling, and/or bathing before sleep

o Quiet music may be helpful.

o Avoid TV and video games for at least 30 minutes before bedtime.

o Keep TV out of the bedroom

**Nutrition**

o Breakfast is essential

o Encourage balanced diet with plenty of protein

o Encourage water limit pop, juices, and other sweetened beverages

o Try to have at least one family meal every day. Sit at the table and talk about the day without the TV.

**Exercise**

o Encourage exercise at least 4 days per week for at least 20 minutes

o Walk in place during TV commercials

o Exercise together as a family

o Consider using a step pedometer to give a measurement of actual exercise

o Set small, concrete goals that are achievable, and build from there.

**Social Support**

- Increasing social support helps to improve moods, behavior, and relationships.
- Expanding social support helps to improve feelings of connectedness and improves communication.
- Purchase some inexpensive invitations or make some at home.
- Ask your child to make a list of people to whom they can give or mail the invitations.
- The invitations are to ask people to be open to a visit or a phone call when support is needed or help is needed with solving problems or some encouragement is needed to improve mood.

**Educational Books for Parents:**

- Understanding Anxiety Disorders in Children, American Academy of Pediatrics, 2018
- Anxiety disorders: Parents’ Medication Guide AACAP, 2020. <https://www.aacap.org/App_Themes/AACAP/docs/resource_centers/resources/med_guides/anxiety-parents-medication-guide.pdf>
- Chansky, Tamar, Freeing Your Child from Anxiety: Powerful, Practical Solutions to Overcome Your Child's Fears, Worries, and Phobias
- Wignall, Ann, Helping your anxious child: A step‐by‐step guide for parents.

**Websites for Parents:**

- [www.worrywisekids.org](http://www.worrywisekids.org) Educational material/tools for parents of children with anxiety disorders. Provided by

The Children’s Center for OCD and Anxiety

- [www.adaa.org](http://www.adaa.org) Anxiety Disorder Association of America. Information, resources and tools for families

**Tools to Help Manage Symptoms of Anxiety**

**Bubbles**

o Encourage taking slow, deep breaths to get as many bubbles as possible

o Do this at least once a day, or as often as needed.

**Play‐Doh**

o Knead, roll, pound, and shape the Play‐Doh

o Do this at least once a day, or as often as needed.

**Textured cloths**

o Corduroy and silky cloths work best

o Rub a material that feels soothing

o Can be kept in a pocket, or attached to the top or bottom of a desk

**Lotion**

o Give a squirt of lotion and ask the child to calm self

o Have child give themselves a hand massage

o Parent can give a hand massage, also

**Superpower Hands**

o Ask child to show how to make fists

o After making fists, hands rise up and muscles tense

o Have child give complete opposite response, relaxing hands

o Superpower hands relax

**“Big Daddy” Sunglasses**

o Can help a child discuss things that are scary or embarrassing

o Give a pair of big, inexpensive sunglasses to help provide some distance for the worries or fears.

o Once the sunglasses are on, no one can “watch” him/her talk about the “scary” thing or embarrassing

behavior.

**Superhero Comic Books or Movies**

o Have the child choose a favorite superhero comic book or movie

o Ask the child to describe the superhero and their superpowers

o Have the child take on the superhero’s power and use it to get through the scary situation.

**Music**

o Have the child listen to soothing, relaxing, and meaningful music before a stressful situation, to calm

after a stressful situation, or at the end of the day to relax and help get to sleep.

**Books and Other Resources**

**Children’s Books:**

- Belknap, Martha, Stress relief for kids: Taming your dragon
- Buron, Kari, When my worries get too big! A relaxation book for children who live with anxiety.
- Crist, James J., PhD., What do you do when you’re SCARED and WORRIED?
- Guanci, Anne, David and the worry beast: Helping children cope with anxiety.
- Huebner, Dawn, What to do when you worry too much: A kid’s guide to overcoming anxiety.
- Shapiro, Lawerence E., The Relaxation and Stress Reduction Workbook for Kids: Help for Children to Cope with
- Stress, Anxiety, and Transitions
- Weaver, Susan B., Worry busters: Activities for kids who worry too much.

**Books for Teens:**

- Schab, Lisa M., The anxiety workbook for teens: Activities to help you deal with anxiety and worry.
- Shannon, Jennifer, The Shyness and Social Anxiety Workbook for Teens: CBT and ACT Skills to Help You Build
- Social Confidence (Instant Help Solutions)
- Sokol, Leslie & Fox, Marci, Think Confident, Be Confident for Teens: A Cognitive Therapy Guide to Overcoming
- Self‐Doubt and Creating Unshakable Self‐Esteem (The Instant Help Solutions Series)
