# Supplementary material for: A Case-Based, Longitudinal Curriculum in Pediatric Behavioral and Mental Health
Source: MedEdPORTAL. 2024 Apr 29;20:11400. doi: 10.15766/mep_2374-8265.11400 (PMC11056487; doi:10.15766/mep_2374-8265.11400)
Supplement: Supplementary file 1 — Preteen Anxiety Case - Residents.docxPreteen Anxiety Case - Faculty Guide.docxPreteen Anxiety Case - SCARED Forms.pdfAnxiety Resources Handout.docxASD Delays Case - Residents.docxASD Delays Case - Faculty Guide.docxAutism Summary Handout and Resources.docxDepression Case - Residents.docxDepression Case - Faculty Guide.docxDepression Resources Handout.docxSchool-age ADHD Case - Residents.docxSchool-age ADHD Case - Faculty Guide.docxSchool-age ADHD Case - Vanderbilts.pdfADHD Handout.docxYoung ADHD and Behavior Case - Residents.docxYoung ADHD and Behavior Case - Faculty Guide.docxParenting Handout and Resource Sheet.docxBehavioral and Mental Health Curriculum Survey.docxBehavioral and Mental Health Pre-Post Test.docx [file mep_2374-8265.11400-s001.zip › G. Autism Summary Handout and Resources.docx]

**Autism Summary Handout and Resources**

***DSM-5 Criteria for Autism Spectrum Disorder***

A1. Deficits in social-emotional reciprocity (the ability to engage with others and share thoughts and feelings)

A2. Deficits (absent, reduced, or atypical relative to cultural norms) in nonverbal communicative behaviors used for social interaction

A3. Deficits (for age, gender, and culture) in developing, maintaining, and understanding relationships

B1. Stereotyped or repetitive motor mannerisms, use of objects, or speech

B2. Insistence on sameness, inflexible adherence to routines, or ritualized patterns of verbal or nonverbal behavior

B3. Highly restricted, fixated interests that are abnormal in intensity or focus

B4. Hyper- or hyporeactivity to sensory input or unusual interest in sensory aspects of the environment

C. Symptoms must be present in the early developmental period (but may not become fully manifest until social demands exceed limited capacities, or may be masked by learned strategies in later life)

D. Symptoms cause clinically significant impairment in social, occupational, or other important areas of current functioning

E. These disturbances are not better explained by intellectual disability (age ≥ 5 years) or global developmental delay (age < 5 years). ID and ASD frequently co-occur; to make comorbid diagnoses of ASD and ID, social communication should be below that expected for general developmental level

***Screening and Testing Tools used for Autism***

1. Modified Checklist for Autism in Toddlers, Revised with Follow-up (M-CHAT-R/F)
   1. An autism-focused screening tool that can be used in the primary care setting
   2. Valid from 16-30 months of age
   3. Filled out by parents, can be scored immediately
   4. Scoring is a risk stratification:
      1. Total score 0-2: low risk; no follow-up needed
      2. Total score 3-7: moderate risk; administer M-CHAT-R follow-up items. If 2 or more are at risk after administering follow-up questions then refer for early intervention and diagnostic evaluation
      3. Total score 8-20: HIGH risk; bypass follow-up items. Refer for early intervention and diagnostic evaluation
2. Ages and Stages Questionnaires: Social-Emotional, Second Edition (ASQ: SE-2)
   1. A narrow-band screening tool (focusing on social/emotional development) that can be used in the primary care setting
   2. Valid from 1-72 months of age
   3. 9 questionnaires range from 2 months to 60 months
   4. Not specific for autism; if specific concerns about autism arise may consider more targeted screening
   5. Scoring is more complex as it involves both competence and problem scores; may need to use manual
   6. If child scores in monitoring zone or above the cutoff, referral is not necessarily indicated; may opt to work with family on behavioral management and have close follow-up
3. Screening Tool for Autism in Toddlers (STAT)
   1. A play-based testing tool that can be used in the primary care or subspecialty setting to assess autism risk
   2. Valid from 24-36 months of age, though children as young as 18 months or as old as 48 months may be tested
   3. Requires specific training and certification to be used, and a specific testing kit and score sheet exist for administration
   4. The test is a screening tool for autism and it gives a risk assessment; it is not diagnostic. However if used by a trained provider as part of a formal assessment it can be used as part of the diagnostic process
   5. Features 12 items from 4 categories focused on social-emotional reciprocity and play: Play, Requesting, Directing Attention, and Imitation
      1. A total score of 2 or greater is considered “at risk” for autism; scores below 2 are not considered at risk
4. Tools used in the subspecialty setting as part of formal diagnosis:
   1. Autism Diagnostic Observation Scale, Second Edition (ADOS-2)
      1. A thorough, formal, structured assessment for autism
      2. Has multiple modules for both nonverbal and verbal patients, including a toddler module for very young patients
   2. Childhood Autism Rating Scale, Second Edition (CARS-2)
      1. An observational assessment in which the child is rated over 15 categories to assess severity of autism symptoms
   3. Gilliam Autism Rating Scale, Third Edition (GARS-3)
      1. A norm-referenced screening instrument used for identifying people with autism
      2. Can be filled out by parents, teachers, or other caregivers who know the child well, and it is scored by a trained examiner
      3. Gives a likelihood of autism based on scores from 6 subscales
      4. Can be used as part of the diagnostic process, and also to assess severity of autism symptoms for those already diagnosed

***Review of Recommendations and Resources for Autism***

1. Treatments for autism: oftentimes, autism-specific therapies are recommended, especially in children who have more severe symptoms or who are younger
   1. Applied Behavior Analysis
   2. Parent-Mediated/play based (e.g. the PLAY Project)
2. Other recommended therapies, if indicated
   1. Speech Therapy
   2. Occupational Therapy
   3. Physical Therapy
   4. Behavioral/Family Therapy
3. Early Intervention Services: for children younger than 3 years, it is recommended that they are enrolled in the state’s early intervention/birth-to-three program if they are not already
4. School-based supports: it is recommended that children 3 and older are evaluated for an IEP so that they can get school-based accommodations and special education supports
5. Medications: there is no medication to treat autism; however, many children on the autism spectrum receive medication treatment due to certain symptoms or diagnoses (e.g. ADHD, aggression, anxiety)
6. It is always recommended that a provider/team managing a child with autism understand resources/community supports in their local area so that they can communicate and share them with the family

*References:*

1. American Psychiatric Association. (2013). Diagnostic and statistical manual of mental disorders (5th ed.). https://doi.org/10.1176/appi.books.9780890425596
2. Hyman SL, Levy SE, Myers SM, et al. Executive Summary: Identification, Evaluation, and Management of Children with Autism Spectrum Disorders. Pediatrics (2020) 145 (1): e20193448
3. [*https://mchatscreen.com/*](https://mchatscreen.com/)
4. [*https://agesandstages.com/products-pricing/asqse-2/*](https://agesandstages.com/products-pricing/asqse-2/)
