# Supplementary material for: A Case-Based, Longitudinal Curriculum in Pediatric Behavioral and Mental Health
Source: MedEdPORTAL. 2024 Apr 29;20:11400. doi: 10.15766/mep_2374-8265.11400 (PMC11056487; doi:10.15766/mep_2374-8265.11400)
Supplement: Supplementary file 1 — Preteen Anxiety Case - Residents.docxPreteen Anxiety Case - Faculty Guide.docxPreteen Anxiety Case - SCARED Forms.pdfAnxiety Resources Handout.docxASD Delays Case - Residents.docxASD Delays Case - Faculty Guide.docxAutism Summary Handout and Resources.docxDepression Case - Residents.docxDepression Case - Faculty Guide.docxDepression Resources Handout.docxSchool-age ADHD Case - Residents.docxSchool-age ADHD Case - Faculty Guide.docxSchool-age ADHD Case - Vanderbilts.pdfADHD Handout.docxYoung ADHD and Behavior Case - Residents.docxYoung ADHD and Behavior Case - Faculty Guide.docxParenting Handout and Resource Sheet.docxBehavioral and Mental Health Curriculum Survey.docxBehavioral and Mental Health Pre-Post Test.docx [file mep_2374-8265.11400-s001.zip › J. Depression Resources Handout.docx]

**Depression Summary Sheet and Resources Handout**

**Key Points:**

- Clarify appropriate mental health dx and r/o comorbid mental health and medical conditions.
- Start antidepressant at low dose. Continue to titrate up slowly looking for response every 4-6 weeks. Treat to complete remission of symptoms.
- Utilize mental health symptom screeners for tracking treatment response
- Utilize safety planning for at-risk patients

**Mental Health Screeners:**

- PHQ-9A: Depression
- CSSRS: Suicide Risk Assessment
- GAD-7: Anxiety
- CYBOC: OCD
- CRAFFT: adolescent substance use
- Vanderbilt: ADHD

**Potential lab workup:**

- Limited evidence for lab screening in absence of other physical symptoms or medical co-morbidities
- Routine labs: CBC, BMP, TSH, UA, UPT, UDS
- Evidence with specific symptoms:
  - Iron/ferritin
  - Vitamin B12/folate
  - Vitamin D
- Limited clinical evidence for pharmacogenetic testing outside of complicated medical co-morbidities or polypharmacy

**Selective Serotonin Reuptake Inhibitors**: R/B/A

- **Benefits:** modulates serotonin in the brain/body, expected to improve mood in approximately 4-6wks at a particular dose.
- **Common side effects:** including GI, HA, drowsiness or activation, sexual SEs
- **Serious adverse effects:** including agitation, mania, suicidal thoughts and behaviors.
- **Black box warning (<24yo):** the potential for increased anxiety, energy, activation, agitation, suicidal ideations and suicidal behaviors, and close monitoring guidelines based on AACAP & FDA recommendations, to ensure the patient’s safety.
- **Alternatives:** multiple other SSRI’s, atypical antidepressants, individual psychotherapy

**Selective Serotonin Reuptake Inhibitors:**

|  | **Starting Dose (1 week)** | **Minimally Effective** | **Dose Change (4-6 weeks)** | **FDA Max** | **Taper (1 week at a time)** |
| --- | --- | --- | --- | --- | --- |
| Fluoxetine (Prozac)  FDA ind. MDD age 8+  FDA ind. OCD age 7+  Liquid avail. | 10 | 20 | 10-20 | 80 | 20 |
| Sertraline (Zoloft)  FDA ind. OCD age 6+  Liquid avail. | 25 | 50 | 25-50 | 200 | 50 |
| Citalopram (Celexa) | 10 | 20 | 10-20 | 40 | 10-20 |
| Escitalopram (Lexapro)  FDA ind MDD age 12+  Liquid avail. | 5 | 10 | 5-10 | 20 (30) | 10 |

Author Owned

**Cross-taper-titration:**

Method 1 (bridge) = as you begin the taper of first agent, begin the second agent at the starting dose, titrate second agent as above without fully tapering the first agent until expected therapeutic response from the second agent. Advantage: less of a nadir in treatment, less serotonin discontinuation. Disadvantage: potential for increased SE and AE including risks for serotonin syndrome

Method 2 (true cross-taper-titration) = begin with taper of first agent and bring down to the start or min effective dose, then begin second agent at start dose. Don’t reach minimally effective dose until the first agent is d/c’d fully. Advantage: better tolerated from a SE/AE standpoint, avoid the trap of dual agent treatment (stopping in the middle because it seems to work best). Disadvantage: nadir in treatment, guaranteed period of lack of efficacy.

There are not necessarily “dose equivalents.” Fluoxetine 80 does not necessarily = sertraline 200, so follow the chart once on the second agent and give each dose a fair trial.

**Making the environment safe (consider these interventions):**

**1. Parents secure prescription/OTC pills, poisons, sharps, weapons, items which could be tied around the neck**

**2. Parents can search room/backpack for unsafe objects**

**3. Parental monitoring/restricting of social media/inappropriate peer relations**

**4. Parental monitoring to include increased time spent with child/teen, limiting isolation**

5. __________

6.

Adapted from Stanley, B. & Brown, G.K. (2011). Safety planning intervention: A brief intervention to mitigate suicide risk. Cognitive and Behavioral Practice. 19, 256–264

**Step 1: Warning signs (ways I know I am feeling distressed):**

**Step 2: Internal coping strategies (things I can do to take my mind off my problems without contacting another person):**

**Step 3: People and social settings that provide healthy distraction:**

**Step 4: People who I can ask for help:**

**Step 5: Professionals or agencies I can contact during a crisis:**

**SAFETY PLAN**

1.

2.

3.

1.

2.

3.

1. Name Phone
2. Name Phone
3. Place
4. Place
5. Name Phone
6. Name Phone
7. Name Phone
8. Clinician Name Phone Clinician Pager or Emergency Contact #
9. Clinician Name Phone Clinician Pager or Emergency Contact #
10. Text Crisis Line: 741741
11. Suicide Prevention Lifeline: 1-800-273-TALK (8255)
12. Local Emergency Service: Crisis Care 224-4646; Dayton Children’s Hospital (937) 641-3000

Emergency Services Address: report to nearest ED, Dayton Children’s Hospital

Emergency Services Phone:

**WPAFB Mental Health Clinic**

88^th^ Medical Group

Wright Patterson AFB, OH 45433-5529
