# Supplementary material for: A Case-Based, Longitudinal Curriculum in Pediatric Behavioral and Mental Health
Source: MedEdPORTAL. 2024 Apr 29;20:11400. doi: 10.15766/mep_2374-8265.11400 (PMC11056487; doi:10.15766/mep_2374-8265.11400)
Supplement: Supplementary file 1 — Preteen Anxiety Case - Residents.docxPreteen Anxiety Case - Faculty Guide.docxPreteen Anxiety Case - SCARED Forms.pdfAnxiety Resources Handout.docxASD Delays Case - Residents.docxASD Delays Case - Faculty Guide.docxAutism Summary Handout and Resources.docxDepression Case - Residents.docxDepression Case - Faculty Guide.docxDepression Resources Handout.docxSchool-age ADHD Case - Residents.docxSchool-age ADHD Case - Faculty Guide.docxSchool-age ADHD Case - Vanderbilts.pdfADHD Handout.docxYoung ADHD and Behavior Case - Residents.docxYoung ADHD and Behavior Case - Faculty Guide.docxParenting Handout and Resource Sheet.docxBehavioral and Mental Health Curriculum Survey.docxBehavioral and Mental Health Pre-Post Test.docx [file mep_2374-8265.11400-s001.zip › A. Preteen Anxiety Case - Residents.docx]

**Case 1**

**Initial Visit**

CC: trouble falling asleep

Stephanie is a 12-year-old girl previously diagnosed with ADHD who is on a stable dose of long-acting methylphenidate (Concerta). Mother brings Stephanie in because she is having a lot of trouble falling asleep at night. Because she’s having trouble sleeping, neither Stephanie nor her parents are getting enough sleep. Stephanie is difficult to rouse in the mornings and has missed some school as a result.

1) What additional information would you like to know?

2) What is the differential diagnosis of anxiety?

3) What screening tools can you use for anxiety?

4) What are the best treatment options for Stephanie?

5) What type of counseling is best?

6) What can we recommend/do in the office for the patient and their parents?

7) Should you start medication, and if yes, what medication?

**Case 1: Preteen Anxiety**

**Follow-up Visit #1 (Virtual/Phone Visit)**

Recap: Stephanie is a 12-year-old female previously diagnosed with ADHD on a stable dose of long-acting methylphenidate (Concerta). At the last visit 2 weeks ago, she was having a lot of trouble falling asleep at night. You discovered some significant underlying generalized anxiety. You provided the family with education about anxiety and some brief interventions that might be helpful. You prescribed sertraline 25 mg daily, continued her long-acting methylphenidate (Concerta) 36 mg daily, and referred Stephanie for outpatient counseling.

1) What do you want to know at this time?

2) What will you do with her medication regimen?

3) What are the tenets of cognitive-behavioral therapy?

**Case 1: Preteen Anxiety**

**Follow-up Visit #2 (Clinic Visit)**

Recap: Stephanie is a 12-year-old female previously diagnosed with ADHD on a stable dose of long-acting methylphenidate (Concerta). At the first visit, she was having a lot of trouble falling asleep at night. You discovered some significant underlying generalized anxiety. You provided the family with education about anxiety and some brief interventions that might be helpful. You prescribed sertraline 25 mg daily, continued her long-acting methylphenidate (Concerta) 36 mg daily, and referred Stephanie for outpatient counseling. At phone follow up 1-2 weeks later, her status was unchanged, you increased her sertraline to 50 mg daily, emphasized the importance of counseling, and provided education on what to expect with CBT.

You are now seeing her 6 weeks after the dose increase to 50mg daily.

1) What do you want to know at this time?

2) What is your next course of action regarding medication?

3) What can Stephanie do in the moment when she’s experiencing panic?
