# Supplementary material for: A Case-Based, Longitudinal Curriculum in Pediatric Behavioral and Mental Health
Source: MedEdPORTAL. 2024 Apr 29;20:11400. doi: 10.15766/mep_2374-8265.11400 (PMC11056487; doi:10.15766/mep_2374-8265.11400)
Supplement: Supplementary file 1 — Preteen Anxiety Case - Residents.docxPreteen Anxiety Case - Faculty Guide.docxPreteen Anxiety Case - SCARED Forms.pdfAnxiety Resources Handout.docxASD Delays Case - Residents.docxASD Delays Case - Faculty Guide.docxAutism Summary Handout and Resources.docxDepression Case - Residents.docxDepression Case - Faculty Guide.docxDepression Resources Handout.docxSchool-age ADHD Case - Residents.docxSchool-age ADHD Case - Faculty Guide.docxSchool-age ADHD Case - Vanderbilts.pdfADHD Handout.docxYoung ADHD and Behavior Case - Residents.docxYoung ADHD and Behavior Case - Faculty Guide.docxParenting Handout and Resource Sheet.docxBehavioral and Mental Health Curriculum Survey.docxBehavioral and Mental Health Pre-Post Test.docx [file mep_2374-8265.11400-s001.zip › B. Preteen Anxiety Case - Faculty Guide.docx]

**Case 1: Preteen Anxiety**

**Julie Stucke PhD and John Duby, MD**

**Learning Objectives:**

By the end of the initial and follow-up cases, learners will be able to:

1. Identify signs and symptoms of anxiety in youth based on DSM-V criteria.
2. Identify at least two screening tools for anxiety and apply them to diagnose and classify anxiety.
3. Develop recommendations for non-pharmacologic and pharmacologic management of anxiety.
4. List and manage adverse effects of medication for ADHD and anxiety.

**Initial Visit**

Chief complaint: Trouble falling asleep for 3 months

Stephanie is a 12-year-old girl previously diagnosed with ADHD who is on a stable dose of long-acting methylphenidate (Concerta). Mother brings Stephanie in because she is having a lot of trouble falling asleep at night. Because she’s having trouble sleeping, neither Stephanie nor her parents are getting enough sleep. Stephanie is difficult to rouse in the mornings and has missed some school as a result.

1. **What additional history would you like?**
   - ***Medication Regimen***
     - *She takes long-acting methylphenidate (Concerta) 36 mg at 730 AM on the days she gets up on time, but it can be an hour later if she gets up late.*
     - *Review of the OARRS report indicates prescriptions have been filled as prescribed*
   - ***Bedtime routines and sleep hygiene***
     - *Stephanie has a decent bedtime routine. She has a snack around 8:00PM; watches something on her phone until 8:30; takes a shower; gets her clothes out for the next day; reads or listens to music; lights out around 9:30; needs music or the sound of the television to fall asleep.*
   - ***What she is thinking about at night when she’s lying in bed? Are you worrying about things at night?***
     - *Stephanie reveals she thinks about a lot of things at night. She often thinks about all the things that happened with her friends that day. Sometimes she imagines things she should have said or done. She worries her friends might be mad at her. Stephanie also thinks about what is happening the next day. She worries a lot about tests and quizzes because she wants good grades so she can get into college. Often, Stephanie worries she has forgotten to do all of her homework, so she gets up and checks to make sure she has finished everything. Sometimes her stomach hurts when she’s lying in bed.*
   - ***Other worries and somatic complaints***
     - *Stephanie sometimes has headaches and stomachaches at school. Parents have had to pick her up from the nurse’s office at least 5 times this year. She worries about her peers liking her and about being as smart as other kids. Sometimes Stephanie worries about her parents being in a car accident, so she insists her parents’ text her when they leave work so she knows when they will arrive home. Stephanie worries before her soccer games and often has significant stomach pain before games. She missed the final game in a tournament recently because she was up much of the night the night before.*
   - ***Common Physical Symptoms of Anxiety***
     - *Tension headache*
     - *Dizziness*
     - *Perioral and fingertip tingling associated with hyperventilation*
     - *“Lump in the throat”*
     - *Can’t swallow pills*
     - *Worry about gagging, choking, swallowing, and vomiting*
     - *Chest pain*
     - *Abdominal pain*
     - *Bowel and bladder urgency*
   - ***How much are symptoms interfering with her functioning?***
     - *Stephanie is missing school or goes in late to school at least once a week because of her trouble falling asleep. Stephanie has not been able to enjoy playing soccer as much this year.*
   - ***Family mental health history for anxiety***

- *Mother, Maternal grandmother and maternal aunt have anxiety disorders. Mother takes sertraline with good results.*
- *There is a strong genetic component to anxiety.*
- *Anxiety is “contagious”*

***Physical Examination:***

*She appears anxious, a bit guarded, but is cooperative and engaged. She has damaged fingernails and cuticles. Otherwise normal PE.*

1. **What is the differential diagnosis of anxiety?**

***Separation anxiety disorder***

- *Developmentally inappropriate and excessive fear or anxiety concerning separation from those to whom the individual is attached, as evidenced by at least 3 of the following:*
  - *Recurrent excessive distress when anticipating or experiencing separation from home or from major*
  - *attachment figures.*
  - *Persistent and excessive worry about losing major attachment figures or about possible harm to them,*
  - *such as illness, injury, disasters, or death.*
  - *Persistent and excessive worry about experiencing an untoward event (e.g., getting lost, being*
  - *kidnapped, having an accident, becoming ill) that causes separation from a major attachment figure.*
  - *Persistent reluctance or refusal to go out, away from home, to school, to work, or elsewhere because of fear of separation.*
  - *Persistent and excessive fear of or reluctance about being alone or without major attachment figures at home or in other settings.*
  - *Persistent reluctance or refusal to sleep away from home or to go to sleep without being near a major attachment figure.*
  - *Repeated complaints of physical symptoms (such as headaches, stomachaches, nausea, or vomiting) when separation from major attachment figures occurs or is anticipated*
- *Causes significant distress for at least 4 weeks*

| ***Generalized Anxiety Disorder***   - *Excessive anxiety and worry, occurring more days than not for at least 6 months, concerning a number of events.* - *The individual finds it difficult to control the worry* - *The anxiety and worry are associated with at least 1 of the following six symptoms:* - *Restlessness, feeling keyed up or on edge.* - *Being easily fatigued* - *Difficulty concentrating* - *Irritability* - *Muscle tension* - *Sleep disturbance*   ***Social Anxiety Disorder*** |
| --- |

- *A persistent fear (6 or more months) of one or more social or performance situations in which the person is exposed to unfamiliar people or to possible scrutiny by others. The individual fears that he or she will act in a way (or show anxiety symptoms) that will be embarrassing and humiliating.*
- *Exposure to the feared situation almost invariably provokes anxiety, which may take the form of situationally bound or situationally pre-disposed Panic Attack.*
- *The person recognizes that this fear is unreasonable or excessive.*
- *The feared situations are avoided or else are endured with intense anxiety and distress.*

***ADHD vs Anxiety***

| ***ADHD*** | ***Anxiety*** |
| --- | --- |
| *Distractibility/inattention for no reason* | *Distractibility/inattention because worrying or nervous* |
| *No physical complaints when not on stimulants* | *Physical complaints* |
| *Difficulty sleeping due to hyperarousal* | *Difficulty sleeping due to anxious preoccupation* |
| *Stimulants decrease difficulties* | *Stimulants may worsen difficulties* |
| *More in boys* | *Children: Boys = Girls Adolescents: Girls > Boys* |

1. **What screening tools can you use for anxiety?**

***Pediatric Symptom Checklist 17 and 35***

***RCMAS-2*** *(Revised Childhood Manifest Anxiety Scale); Gives a total anxiety score along with scores for Worry and Oversensitivity, Physical Symptoms of Anxiety, and Social Concerns (this form is not free and is only for the child to complete)*

***SCARED*** ***(Screen for Childhood Anxiety Related Disorders)***

*Free and online;* [*https://www.midss.org/content/screen-child-anxiety-related-disorders-scared*](https://www.midss.org/content/screen-child-anxiety-related-disorders-scared)

*Provides an overall anxiety score along with scores in the following categories:*

*Panic Disorder or Significant Somatic Symptoms*

*Generalized Anxiety Disorder*

*Separation Anxiety*

*Social Anxiety Disorder,*

*Significant School Avoidance*

*Can be used as early as 4 years old*

**Provide Learners with these SCARED Scoring Forms**

Child Form:


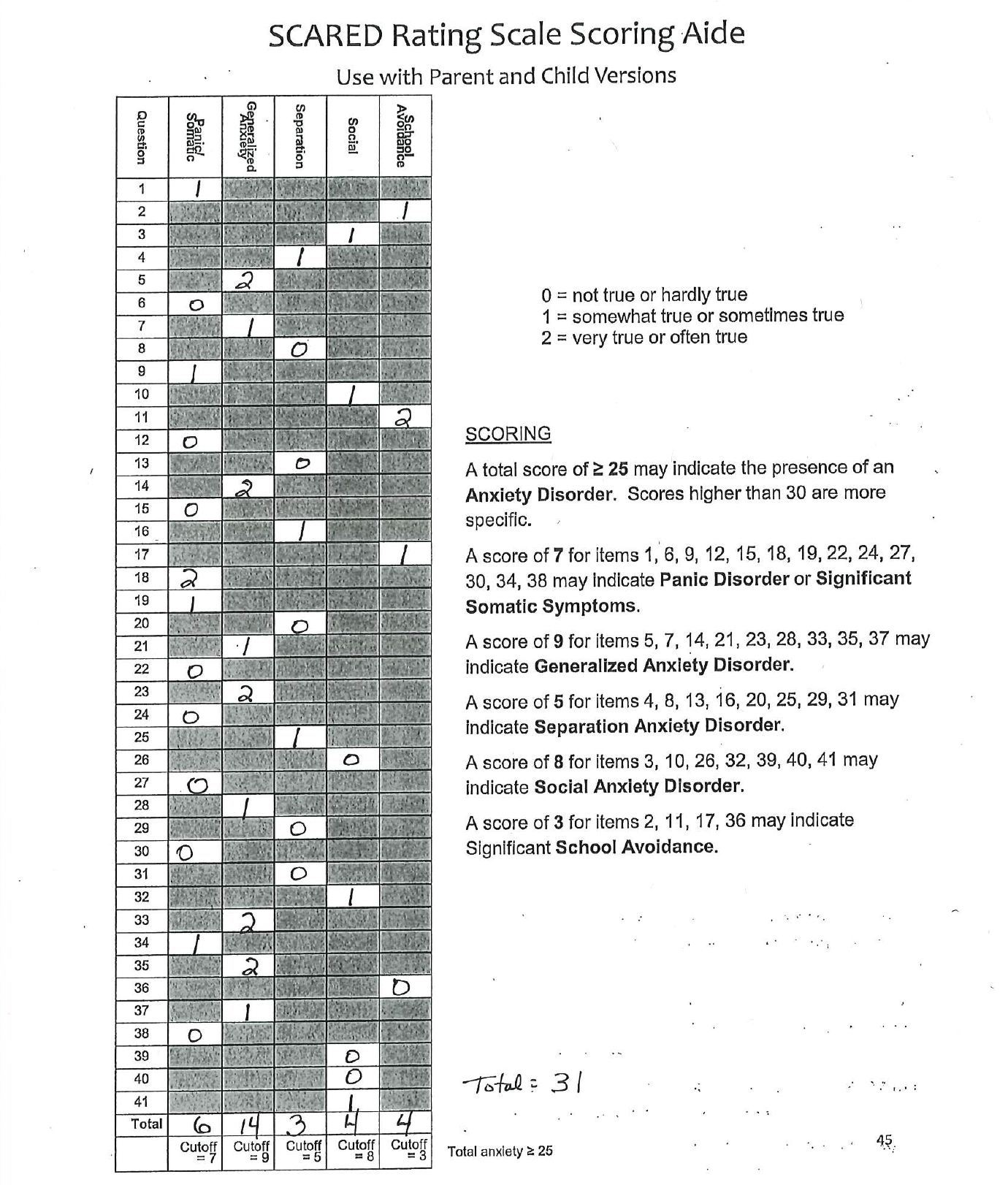


SCARED form copyrighted and owned by Dr. Boris Birmaher. Permission received from Dr. Boris Birmaher on March 5, 2023.

Parent Form:


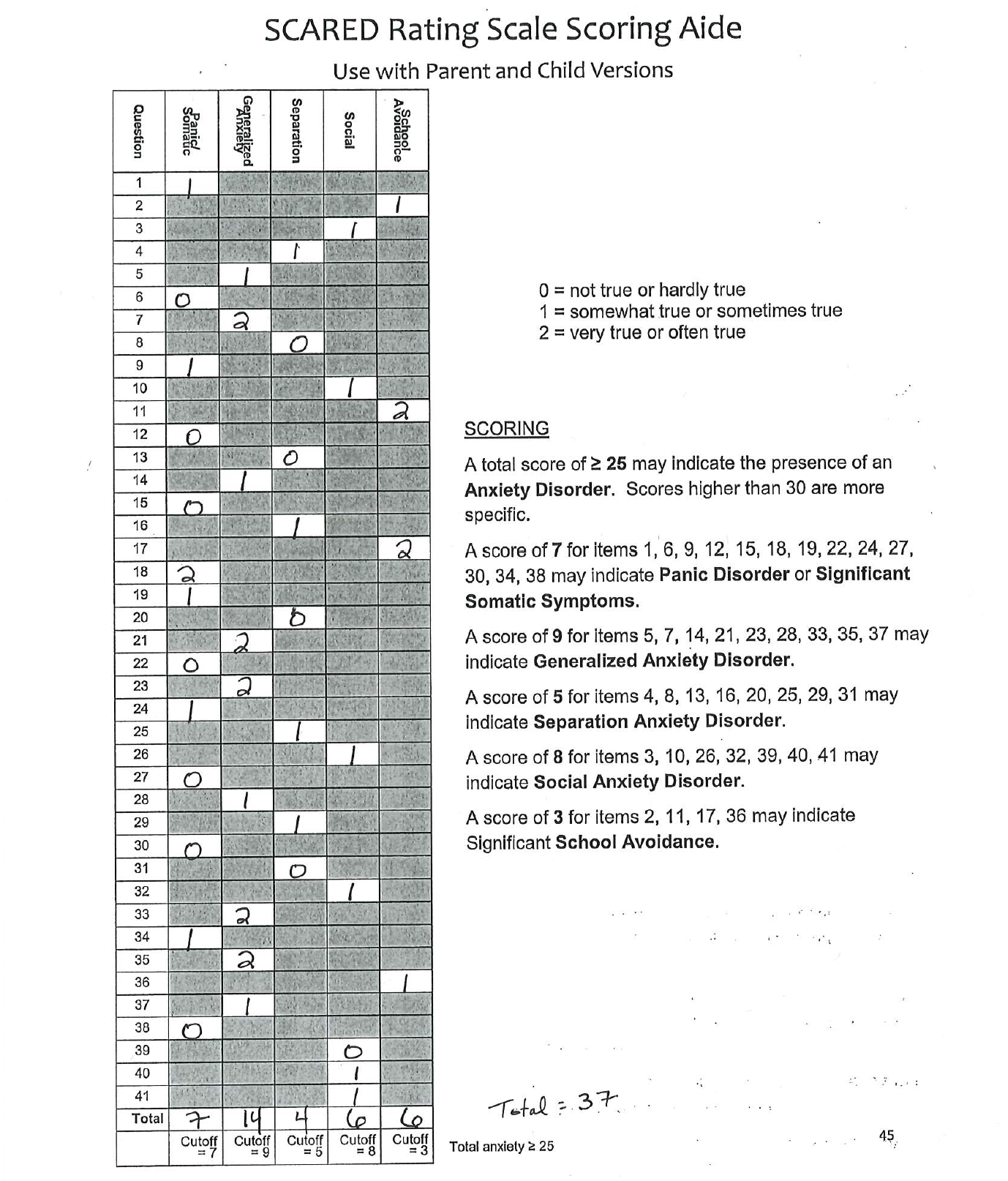


SCARED form copyrighted and owned by Dr. Boris Birmaher. Permission received from Dr. Boris Birmaher on March 5, 2023.

1. **What are the best treatment options for Stephanie?**

- *Counseling is the first line of treatment if the anxiety is mild. The length of time the child has had the anxiety, frequency of the anxiety, and the degree to which the anxiety is impairing the child will help determine whether medication is started along with counseling.*
- *Combination treatment with medication and counseling is preferred for most cases.*
  - *80% response rate*
  - *SSRI’s or CBT alone: 55-60% response*
  - *Placebo response less than 25%*

1. **What type of counseling is best?**

*Cognitive-Behavioral Therapy*

- *Challenge negative thoughts*
- *Develop positive actions*
- *Develop coping mechanisms*

1. **What can we recommend/do in the office for the patient and their parents?**

- ***Educate*** *the patient and family about anxiety – this includes normalizing anxiety and reassuring the patient that some anxiety is a good thing. We can talk about how small amounts of anxiety stop us from doing dangerous things or from getting in trouble at school. It can help motivate us to study so we do better in school. However, anxiety is not a good thing when it makes our bodies feel badly and when it stops us from doing things we want to do or need to do.*
- ***Book or online resources for parents and child***
- ***Exposure****: Remind mother and Stephanie it is not a good idea to avoid the things Stephanie feels anxious about, as this will only make her anxiety stronger.*
- *Mention* ***relaxation*** *apps available on mobile phones (Mindfulness for Children; Thrive Global); Encourage parent to practice these with their child.*
- ***Worry Box:*** *Suggest the child and parent choose a specific time each day for the child to talk to her parent about her worries. Any other time when a worry pops into her mind, the child is supposed to imagine a box with a large lock on it. She is to put the worry into the box and leave it there until it is time to talk to her parent about it. Parents are to listen to and acknowledge their child’s worries and provide reassurance, comfort, and even problem-solving strategies if appropriate.*

1. **Should you start medication, and if yes, what medication**

- *SSRI’s are first line treatment*
  - *Start sertraline 25 mg daily, especially since mother does well with it*
  - *Continue long-acting methylphenidate (Concerta) 36 mg daily*
- *FDA approved based on studies of obsessive-compulsive disorder*
  - *Sertraline*
  - *Fluoxetine*
  - *Fluvoxamine*
  - *Duloxetine is the only FDA approved medication for anxiety, but not as effective as SSRIs*
- *Takes 4-6 weeks to see benefits*
- *Likely to need dose adjusted to be effective*
- *Side-effects of SSRIs: Headache, gastric distress, insomnia*

**FDA Approved Medications**

| 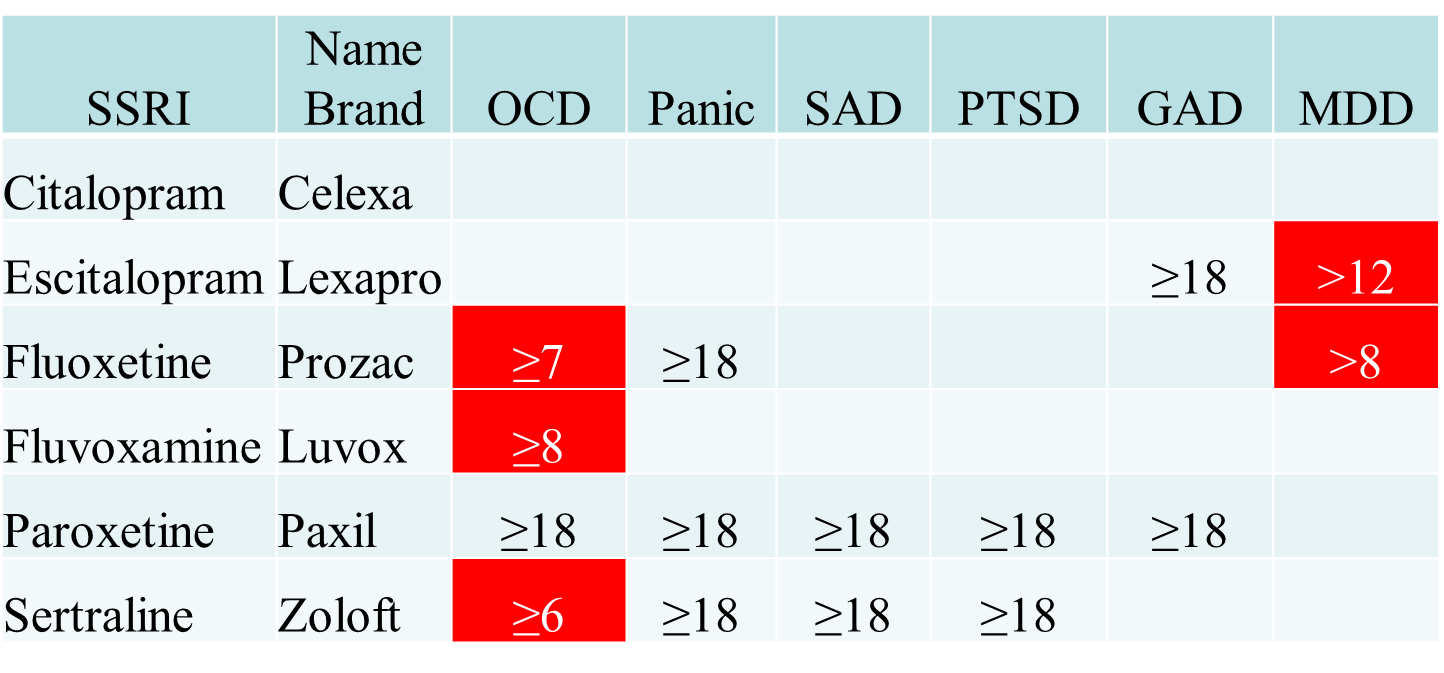 | Name Brand | OCD | Panic | SAD | PTSD | GAD | MDD |
| --- | --- | --- | --- | --- | --- | --- | --- |

Author Owned

**Dosing Chart:**

- Begin at starting dose, then increase to minimum effective dose if tolerated within 1-2 weeks

| **SSRI** | **Starting Dose (mg)** | **Increments (mg)** | **Effective Dose (mg)** | **Maximum Dose, (mg)** |
| --- | --- | --- | --- | --- |
| Fluoxetine  (Prozac®) | 10 | 10-20 | 20 | 60 |
| Citalopram  (Celexa®) | 10 | 10 | 20 | 40* |
| Fluvoxamine  (Luvox®) | 50 | 50 | 150 | 300 |
| Sertraline  (Zoloft®) | 25 | 12.5-25 | 50 | 200 |
| Escitalopram  (Lexapro®) | 5 | 5 | 10 | 20 |

- Each dose adjustment after that should take at least 4-6 weeks to see full effect

Author Owned

**Case 1: Preteen Anxiety**

**Follow-up Visit #1 (Virtual/Phone Visit)**

Recap: Stephanie is a 12-year-old female previously diagnosed with ADHD on a stable dose of long-acting methylphenidate (Concerta). At the last visit 2 weeks ago, she was having a lot of trouble falling asleep at night. You discovered some significant underlying generalized anxiety. You provided the family with education about anxiety and some brief interventions that might be helpful. You prescribed sertraline 25 mg daily, continued her long-acting methylphenidate (Concerta) 36 mg daily, and referred Stephanie for outpatient counseling.

1. **What do you want to know at this time?**

- Medication benefits or side effects
- You find out Stephanie is not having any side effects from the SSRI, but there hasn’t been any change since starting the SSRI 2 weeks ago.

1. **What will you do with her medication regimen?**

- Increase sertraline to 50 mg daily.
- Continue long-acting methylphenidate (Concerta) 36 mg daily.

1. **What are the tenets of cognitive-behavioral therapy?**

- Stephanie’s mother has made an appointment to meet with a child psychologist and asks you what they can expect when they see the psychologist. What will you tell her?
- Inform mother that all therapists work a little differently, but she can expect that at some point the psychologist will want to spend some time alone with both mother and Stephanie, since most children/teens tend to be a little more open when they aren’t speaking in front of their parents.
- Explain that the psychologist will ask both mother and Stephanie a lot of questions, as he/she is trying to figure out exactly what is going on with Stephanie and what type of treatment will be most helpful. Mother and Stephanie may be asked to complete some questionnaires.
- Explain that mother and child should both feel comfortable with the therapist, since a good client-therapist relationship is important to the success of therapy.
- **Describe the tenets of Cognitive-Behavioral Therapy:**
  - CBT is goal-oriented and problem focused and focuses on what is happening currently.
  - CBT emphasizes collaboration and active participation, so children may get homework assignments to complete at home and parents may need to help with the homework at times.
  - CBT teaches children to identify, evaluate, and respond to the “dysfunctional” thoughts they are having that are contributing to their problems.
  - CBT uses a variety of techniques to change thinking, mood, and behavior. Children are often taught specific strategies for coping with their difficulties.

**Case 1: Preteen Anxiety**

**Follow-up Visit #2 (Clinic Visit)**

Recap: Stephanie is a 12-year-old female previously diagnosed with ADHD on a stable dose of long-acting methylphenidate (Concerta). At the first visit, she was having a lot of trouble falling asleep at night. You discovered some significant underlying generalized anxiety. You provided the family with education about anxiety and some brief interventions that might be helpful. You prescribed sertraline 25 mg daily, continued her long-acting methylphenidate (Concerta) 36 mg daily, and referred Stephanie for outpatient counseling. At phone follow up 1-2 weeks later, her status was unchanged, you increased her sertraline to 50 mg daily, emphasized the importance of counseling, and provided education on what to expect with CBT.

You are now seeing her 6 weeks after the dose increase to 50mg daily.

1. **What do you want to know at this time?**

*Mother reports Stephanie continues to have problems with anxiety. She might be sleeping a little better but still complains of stomach pain quite often and has been late to school a time or two. In addition, Stephanie now seems to be having what mother refers to as “anxiety attacks.” Recently, when Stephanie realized she forgot to complete a homework assignment for that day, she began sweating and crying, her heart was racing, she reported she felt like she couldn’t breathe, and she thought she might vomit.*

1. **What is your next course of action with regard to medication?**

- *Continue to up-titrate the sertraline to maximum dose of 200 mg*
- *Medication treatment options to address the panic:*
  - *Hydroxyzine*
  - *Propranolol*
  - *Benzodiazepines (generally avoid)*

1. **What can Stephanie do in the moment when she’s experiencing panic?**

- *Recommend Stephanie focus on her breathing by breathing in slowly through her nose (count to 5) and blowing the air out through her mouth (count to 5) (“Smell the roses and blow out the candle”)*
- *Once she has found her breath, Stephanie can go through the following steps to help ground herself: (Mother can help with this activity if she is present)*
  - *5: Acknowledge FIVE things she sees around her.*
  - *4: Acknowledge FOUR things she can touch around her.*
  - *3: Acknowledge THREE things she can hear.*
  - *2: Acknowledge TWO things she can smell.*
  - *1: Acknowledge ONE thing she can taste*

References

Creswell, C., Waite, P. and Hudson, J. (2020), Practitioner Review: Anxiety disorders in children and young people – assessment and treatment. J Child Psychol Psychiatr, 61: 628-643. <https://doi.org/10.1111/jcpp.13186>

Strawn JR, Lu L, Peris TS, Levine A, Walkup JT. Research Review: Pediatric anxiety disorders - what have we learnt in the last 10 years? J Child Psychol Psychiatry. 2021 Feb;62(2):114-139. doi: 10.1111/jcpp.13262. Epub 2020 Jun 5. PMID: 32500537; PMCID: PMC7718323.

Walkup, John T. Anxiety: Pediatric Mental Health Minute Series. American Academy of Pediatrics, 2020

American Psychiatric Association. (2013). Anxiety Disorders. In Diagnostic and statistical manual of mental disorders (5th ed.).

The Screen for Child Anxiety Related Disorders[. https://www.midss.org/content/screen-child-anxiety-related-disorders-scared](https://www.midss.org/content/screen-child-anxiety-related-disorders-scared)

Birmaher, B., Brent, D. A., Chiappetta, L., Bridge, J., Monga, S., & Baugher, M. (1999). Psychometric properties of the Screen for Child Anxiety Related Emotional Disorders (SCARED): A replication study. Journal of the American Academy of Child and Adolescent Psychiatry, 38(10), 1230–6.
