# Supplementary material for: A Case-Based, Longitudinal Curriculum in Pediatric Behavioral and Mental Health
Source: MedEdPORTAL. 2024 Apr 29;20:11400. doi: 10.15766/mep_2374-8265.11400 (PMC11056487; doi:10.15766/mep_2374-8265.11400)
Supplement: Supplementary file 1 — Preteen Anxiety Case - Residents.docxPreteen Anxiety Case - Faculty Guide.docxPreteen Anxiety Case - SCARED Forms.pdfAnxiety Resources Handout.docxASD Delays Case - Residents.docxASD Delays Case - Faculty Guide.docxAutism Summary Handout and Resources.docxDepression Case - Residents.docxDepression Case - Faculty Guide.docxDepression Resources Handout.docxSchool-age ADHD Case - Residents.docxSchool-age ADHD Case - Faculty Guide.docxSchool-age ADHD Case - Vanderbilts.pdfADHD Handout.docxYoung ADHD and Behavior Case - Residents.docxYoung ADHD and Behavior Case - Faculty Guide.docxParenting Handout and Resource Sheet.docxBehavioral and Mental Health Curriculum Survey.docxBehavioral and Mental Health Pre-Post Test.docx [file mep_2374-8265.11400-s001.zip › C. Preteen Anxiety Case - SCARED Forms.pdf]

Completed SCARED For Stephanie to be scored

Screen for Child Anxiety Related Disorders (SCARED)

Child Version - Page 1 of 2 (To be filled out by the CHILD)

Name: Stephanie **Date:** ____________

Directions:
Below is a list of sentences that describe how people feel. Read each phrase and decide if it is "Not True or Hardly Ever True" or "Somewhat True or Sometimes True" or "Very True or Often True" for you. Then for each sentence, fill in one circle that corresponds to the response that seems to describe you for the last 3 months.

		0 Not True or Hardly Ever True	1 Somewhat True or Sometimes True	2 Very True or Often True
1.	When I feel frightened, it is hard for me to breathe	O	●	O
2.	I get headaches when I am at school	O	●	O
3.	I don't like to be with people I don't know well	O	●	O
4.	I get scared if I sleep away from home	O	●	O
5.	I worry about other people liking me	O	O	●
6.	When I get frightened, I feel like passing out	●	O	O
7.	I am nervous	O	●	O
8.	I follow my mother or father wherever they go	●	O	O
9.	People tell me that I look nervous	O	●	O
10.	I feel nervous with people I don't know well	O	●	O
11.	[redacted] I get stomachaches at school	O	O	●
12.	When I get frightened, I feel like I am going crazy	●	O	O
13.	I worry about sleeping alone	●	O	O
14.	I worry about being as good as other kids	O	O	●
15.	When I get frightened, I feel like things are not real	●	O	O
16.	I have nightmares about something bad happening to my parents	O	●	O
17.	I worry about going to school	O	●	O
18.	When I get frightened, my heart beats fast	O	O	●
19.	I get shaky	O	●	O
20.	I have nightmares about something bad happening to me	●	O	O

Screen for Child Anxiety Related Disorders (SCARED)

Child Version - Page 2 of 2 (To be filled out by the CHILD)

		0 Not True or Hardly Ever True	1 Somewhat True or Sometimes True	2 Very True or Often True
21.	I worry about things working out for me	○	●	○
22.	When I get frightened, I sweat a lot	●	○	○
23.	I am a worrier	○	○	●
24.	I get really frightened for no reason at all	●	○	○
25.	I am afraid to be alone in the house	○	●	○
26.	It is hard for me to talk with people I don't know well	●	○	○
27.	When I get frightened, I feel like I am choking	●	○	○
28.	People tell me that I worry too much	○	●	○
29.	I don't like to be away from my family	●	○	○
30.	I am afraid of having anxiety (or panic) attacks	●	○	○
31.	I worry that something bad might happen to my parents	●	○	○
32.	I feel shy with people I don't know well	○	●	○
33.	I worry about what is going to happen in the future	○	○	●
34.	When I get frightened, I feel like throwing up	○	●	○
35.	I worry about how well I do things	○	○	●
36.	I am scared to go to school	●	○	○
37.	I worry about things that have already happened	○	●	○
38.	When I get frightened, I feel dizzy	●	○	○
39.	I feel nervous when I am with other children or adults and I have to do something while they watch me (for example: read aloud, speak, play a game, play a sport)	●	○	○
40.	I feel nervous when I am going to parties, dances, or any place where there will be people that I don't know well	●	○	○
41.	I am shy	○	●	○

For children ages 8 to 11, it is recommended that the clinician explain all questions, or have the child answer the questionnaire sitting with an adult in case they have any questions.

Developed by Boris Birmaher, MD, Suneeta Khetarpal, MD, Marlane Cully, MEd, David Brent, MD, and Sandra McKenzie, PhD. Western Psychiatric Institute and Clinic, University of Pgh. (10/95). Email: birmaherb@msx.upmc.edu

SCARED Rating Scale Scoring Aide

Use with Parent and Child Versions

Question	Panic/ Somatic	Generalized Anxiety	Separation	Social	School Avoidance
1					
2					
3					
4					
5					
6					
7					
8					
9					
10					
11					
12					
13					
14					
15					
16					
17					
18					
19					
20					
21					
22					
23					
24					
25					
26					
27					
28					
29					
30					
31					
32					
33					
34					
35					
36					
37					
38					
39					
40					
41					
Total					
	Cutoff = 7	Cutoff = 9	Cutoff = 5	Cutoff = 8	Cutoff = 3

Total anxiety ≥ 25

0 = not true or hardly true
1 = somewhat true or sometimes true
2 = very true or often true

SCORING

A total score of **≥ 25** may indicate the presence of an **Anxiety Disorder.** Scores higher than 30 are more specific.

A score of **7** for items 1, 6, 9, 12, 15, 18, 19, 22, 24, 27, 30, 34, 38 may indicate **Panic Disorder** or **Significant Somatic Symptoms.**

A score of **9** for items 5, 7, 14, 21, 23, 28, 33, 35, 37 may indicate **Generalized Anxiety Disorder.**

A score of **5** for items 4, 8, 13, 16, 20, 25, 29, 31 may indicate **Separation Anxiety Disorder.**

A score of **8** for items 3, 10, 26, 32, 39, 40, 41 may indicate **Social Anxiety Disorder.**

A score of **3** for items 2, 11, 17, 36 may indicate Significant **School Avoidance.**

Screen for Child Anxiety Related Disorders (SCARED)

Parent Version - Page 1 of 2 (To be filled out by the PARENT)

Name: Mary (Stephanie's Mom) **Date:** ____________________

Directions:
Below is a list of statements that describe how people feel. Read each statement carefully and decide if it is "Not True or Hardly Ever True" or "Somewhat True or Sometimes True" or "Very True or Often True" for your child. Then for each statement, fill in one circle that corresponds to the response that seems to describe your child for the last 3 months. Please respond to all statements as well as you can, even if some do not seem to concern your child.

		0 Not True or Hardly Ever True	1 Somewhat True or Sometimes True	2 Very True or Often True
1.	When my child feels frightened, it is hard for him/her to breathe	○	●	○
2.	My child gets headaches when he/she is at school	○	●	○
3.	My child doesn't like to be with people he/she doesn't know well	○	●	○
4.	My child gets scared if he/she sleeps away from home	○	●	○
5.	My child worries about other people liking him/her	○	●	○
6.	When my child gets frightened, he/she feels like passing out	●	○	○
7.	My child is nervous	○	○	●
8.	My child follows me wherever I go	●	○	○
9.	People tell me that my child looks nervous	○	●	○
10.	My child feels nervous with people he/she doesn't know well	○	●	○
11.	My child gets stomachaches at school	○	○	●
12.	When my child gets frightened, he/she feels like he/she is going crazy	●	○	○
13.	My child worries about sleeping alone	●	○	○
14.	My child worries about being as good as other kids	○	●	○
15.	When he/she gets frightened, he/she feels like things are not real	●	○	○
16.	My child has nightmares about something bad happening to his/her parents	○	●	○
17.	My child worries about going to school	○	○	●
18.	When my child gets frightened, his/her heart beats fast	○	○	●
19.	He/she gets shaky	○	●	○
20.	My child has nightmares about something bad happening to him/her	●	○	○

Screen for Child Anxiety Related Disorders (SCARED)

Parent Version - Page 2 of 2 (To be filled out by the PARENT)

		0<br>Not True or Hardly Ever True	1<br>Somewhat True or Sometimes True	2<br>Very True or Often True
21.	My child worries about things working out for him/her	o	o	●
22.	When my child gets frightened, he/she sweats a lot	●	o	o
23.	My child is a worrier	o	o	●
24.	My child gets really frightened for no reason at all	o	●	o
25.	My child is afraid to be alone in the house	o	●	o
26.	It is hard for my child to talk with people he/she doesn't know well	o	●	o
27.	When my child gets frightened, he/she feels like he/she is choking	●	o	o
28.	People tell me that my child worries too much	o	●	o
29.	My child doesn't like to be away from his/her family	o	●	o
30.	My child is afraid of having anxiety (or panic) attacks	●	o	o
31.	My child worries that something bad might happen to his/her parents	●	o	o
32.	My child feels shy with people he/she doesn't know well	o	●	o
33.	My child worries about what is going to happen in the future	o	o	●
34.	When my child gets frightened, he/she feels like throwing up	o	●	o
35.	My child worries about how well he/she does things	o	o	●
36.	My child is scared to go to school	o	●	o
37.	My child worries about things that have already happened	o	●	o
38.	When my child gets frightened, he/she feels dizzy	●	o	o
39.	My child feels nervous when he/she is with other children or adults and he/she has to do something while they watch him/her (for example: read aloud, speak, play a game, play a sport)	●	o	o
40.	My child feels nervous when he/she is going to parties, dances, or any place where there will be people that he/she doesn't know well	o	●	o
41.	My child is shy	o	●	o

Developed by Boris Birmaher, MD, Suneeta Khetarpal, MD, Marlane Cully, MEd, David Brent, MD, and Sandra McKenzie, PhD. Western Psychiatric Institute and Clinic, University of Pgh. (10/95). Email: birmaherb@msx.upmc.edu

SCARED Rating Scale Scoring Aide

Use with Parent and Child Versions

Question	Panic/ Somatic	Generalized Anxiety	Separation	Social	School Avoidance
1					
2					
3					
4					
5					
6					
7					
8					
9					
10					
11					
12					
13					
14					
15					
16					
17					
18					
19					
20					
21					
22					
23					
24					
25					
26					
27					
28					
29					
30					
31					
32					
33					
34					
35					
36					
37					
38					
39					
40					
41					
Total					
	Cutoff = 7	Cutoff = 9	Cutoff = 5	Cutoff = 8	Cutoff = 3

Total anxiety ≥ 25

0 = not true or hardly true
1 = somewhat true or sometimes true
2 = very true or often true

SCORING

A total score of ≥ **25** may indicate the presence of an **Anxiety Disorder.** Scores higher than 30 are more specific.

A score of **7** for items 1, 6, 9, 12, 15, 18, 19, 22, 24, 27, 30, 34, 38 may indicate **Panic Disorder** or **Significant Somatic Symptoms.**

A score of **9** for items 5, 7, 14, 21, 23, 28, 33, 35, 37 may indicate **Generalized Anxiety Disorder.**

A score of **5** for items 4, 8, 13, 16, 20, 25, 29, 31 may indicate **Separation Anxiety Disorder.**

A score of **8** for items 3, 10, 26, 32, 39, 40, 41 may indicate **Social Anxiety Disorder.**

A score of **3** for items 2, 11, 17, 36 may indicate Significant **School Avoidance.**

Blank SCARED forms for future USE

Screen for Child Anxiety Related Disorders (SCARED)

Parent Version - Page 1 of 2 (To be filled out by the PARENT)

Name: ______________________ **Date:** __________________________

Directions:
Below is a list of statements that describe how people feel. Read each statement carefully and decide if it is "Not True or Hardly Ever True" or "Somewhat True or Sometimes True" or "Very True or Often True" for your child. Then for each statement, fill in one circle that corresponds to the response that seems to describe your child for the last 3 months. Please respond to all statements as well as you can, even if some do not seem to concern your child.

		0<br>Not True or Hardly Ever True	1<br>Somewhat True or Sometimes True	2<br>Very True or Often True
1.	When my child feels frightened, it is hard for him/her to breathe	O	O	O
2.	My child gets headaches when he/she is at school	O	O	O
3.	My child doesn't like to be with people he/she doesn't know well	O	O	O
4.	My child gets scared if he/she sleeps away from home	O	O	O
5.	My child worries about other people liking him/her	O	O	O
6.	When my child gets frightened, he/she feels like passing out	O	O	O
7.	My child is nervous	O	O	O
8.	My child follows me wherever I go	O	O	O
9.	People tell me that my child looks nervous	O	O	O
10.	My child feels nervous with people he/she doesn't know well	O	O	O
11.	My child gets stomachaches at school	O	O	O
12.	When my child gets frightened, he/she feels like he/she is going crazy	O	O	O
13.	My child worries about sleeping alone	O	O	O
14.	My child worries about being as good as other kids	O	O	O
15.	When he/she gets frightened, he/she feels like things are not real	O	O	O
16.	My child has nightmares about something bad happening to his/her parents	O	O	O
17.	My child worries about going to school	O	O	O
18.	When my child gets frightened, his/her heart beats fast	O	O	O
19.	He/she gets shaky	O	O	O
20.	My child has nightmares about something bad happening to him/her	O	O	O

SCARED Rating Scale Scoring Aide

Use with Parent and Child Versions

Question	Panic/ Somatic	Generalized Anxiety	Separation	Social	School Avoidance
1					
2					
3					
4					
5					
6					
7					
8					
9					
10					
11					
12					
13					
14					
15					
16					
17					
18					
19					
20					
21					
22					
23					
24					
25					
26					
27					
28					
29					
30					
31					
32					
33					
34					
35					
36					
37					
38					
39					
40					
41					
Total					
	Cutoff = 7	Cutoff = 9	Cutoff = 5	Cutoff = 8	Cutoff = 3

Total anxiety ≥ 25

0 = not true or hardly true
1 = somewhat true or sometimes true
2 = very true or often true

SCORING

A total score of ≥ **25** may indicate the presence of an **Anxiety Disorder.** Scores higher than 30 are more specific.

A score of **7** for items 1, 6, 9, 12, 15, 18, 19, 22, 24, 27, 30, 34, 38 may indicate **Panic Disorder** or **Significant Somatic Symptoms.**

A score of **9** for items 5, 7, 14, 21, 23, 28, 33, 35, 37 may indicate **Generalized Anxiety Disorder.**

A score of **5** for items 4, 8, 13, 16, 20, 25, 29, 31 may indicate **Separation Anxiety Disorder.**

A score of **8** for items 3, 10, 26, 32, 39, 40, 41 may indicate **Social Anxiety Disorder.**

A score of **3** for items 2, 11, 17, 36 may indicate Significant **School Avoidance.**

Screen for Child Anxiety Related Disorders (SCARED)

<u>Child Version</u> - Page 1 of 2 (To be filled out by the CHILD)

Name: ______________________ **Date:** ___________________________

Directions:
Below is a list of sentences that describe how people feel. Read each phrase and decide if it is "Not True or Hardly Ever True" or "Somewhat True or Sometimes True" or "Very True or Often True" for you. Then for each sentence, fill in one circle that corresponds to the response that seems to describe you <u>for the last 3 months</u>.

		**0 Not True or Hardly Ever True**	**1 Somewhat True or Sometimes True**	**2 Very True or Often True**
1.	When I feel frightened, it is hard for me to breathe	O	O	O
2.	I get headaches when I am at school	O	O	O
3.	I don't like to be with people I don't know well	O	O	O
4.	I get scared if I sleep away from home	O	O	O
5.	I worry about other people liking me	O	O	O
6.	When I get frightened, I feel like passing out	O	O	O
7.	I am nervous	O	O	O
8.	I follow my mother or father wherever they go	O	O	O
9.	People tell me that I look nervous	O	O	O
10.	I feel nervous with people I don't know well	O	O	O
11.	My I get stomachaches at school	O	O	O
12.	When I get frightened, I feel like I am going crazy	O	O	O
13.	I worry about sleeping alone	O	O	O
14.	I worry about being as good as other kids	O	O	O
15.	When I get frightened, I feel like things are not real	O	O	O
16.	I have nightmares about something bad happening to my parents	O	O	O
17.	I worry about going to school	O	O	O
18.	When I get frightened, my heart beats fast	O	O	O
19.	I get shaky	O	O	O
20.	I have nightmares about something bad happening to me	O	O	O

SCARED Rating Scale Scoring Aide

Use with Parent and Child Versions

Question	Panic/ Somatic	Generalized Anxiety	Separation	Social	School Avoidance
1					
2					
3					
4					
5					
6					
7					
8					
9					
10					
11					
12					
13					
14					
15					
16					
17					
18					
19					
20					
21					
22					
23					
24					
25					
26					
27					
28					
29					
30					
31					
32					
33					
34					
35					
36					
37					
38					
39					
40					
41					
Total					
	Cutoff = 7	Cutoff = 9	Cutoff = 5	Cutoff = 8	Cutoff = 3

Total anxiety ≥ 25

0 = not true or hardly true
1 = somewhat true or sometimes true
2 = very true or often true

SCORING

A total score of **≥ 25** may indicate the presence of an **Anxiety Disorder.** Scores higher than 30 are more specific.

A score of **7** for items 1, 6, 9, 12, 15, 18, 19, 22, 24, 27, 30, 34, 38 may indicate **Panic Disorder** or **Significant Somatic Symptoms.**

A score of **9** for items 5, 7, 14, 21, 23, 28, 33, 35, 37 may indicate **Generalized Anxiety Disorder.**

A score of **5** for items 4, 8, 13, 16, 20, 25, 29, 31 may indicate **Separation Anxiety Disorder.**

A score of **8** for items 3, 10, 26, 32, 39, 40, 41 may indicate **Social Anxiety Disorder.**

A score of **3** for items 2, 11, 17, 36 may indicate Significant **School Avoidance.**
