# Supplementary material for: A Case-Based, Longitudinal Curriculum in Pediatric Behavioral and Mental Health
Source: MedEdPORTAL. 2024 Apr 29;20:11400. doi: 10.15766/mep_2374-8265.11400 (PMC11056487; doi:10.15766/mep_2374-8265.11400)
Supplement: Supplementary file 1 — Preteen Anxiety Case - Residents.docxPreteen Anxiety Case - Faculty Guide.docxPreteen Anxiety Case - SCARED Forms.pdfAnxiety Resources Handout.docxASD Delays Case - Residents.docxASD Delays Case - Faculty Guide.docxAutism Summary Handout and Resources.docxDepression Case - Residents.docxDepression Case - Faculty Guide.docxDepression Resources Handout.docxSchool-age ADHD Case - Residents.docxSchool-age ADHD Case - Faculty Guide.docxSchool-age ADHD Case - Vanderbilts.pdfADHD Handout.docxYoung ADHD and Behavior Case - Residents.docxYoung ADHD and Behavior Case - Faculty Guide.docxParenting Handout and Resource Sheet.docxBehavioral and Mental Health Curriculum Survey.docxBehavioral and Mental Health Pre-Post Test.docx [file mep_2374-8265.11400-s001.zip › E. ASD Delays Case - Residents.docx]

**Case 2**

**Initial Visit**

CC: speech delay, wellness check

Jackson is a 24-month-old boy who presents with his mother for a 2-year wellness check and to discuss concerns about his speech and language development. He has generally been healthy, and per his mother he seems to be developing appropriately with the exception of his expressive language skills. She thinks he understands language better than he is able to speak it, and she says that he will sometimes gesture to his nose and head when you ask him to locate those body parts. Still, he does not always respond to his name and his ability to carry out commands (even simple one-step commands) is variable. Physically, he appears to be meeting all of his gross motor milestones on time, and he is learning to do more things independently.

1) What more information from the history would you like?

2) How would you go about evaluating development in this 2-year-old?

3) Please look at the ASQ and MCHAT-R forms provided. What do you think about the scores on the screening tools?

4) Your physical exam is normal with the exception of his head appearing rather large but symmetric (when you measure it, it is 51cm – the 95^th^ percentile). What findings might you look for during your developmental surveillance to help you better understand this child’s development?

5) What is your differential diagnosis for this child?

6) What features in this child are concerning for autism? Is there anything that reassures you against autism? How would you delineate autism versus a developmental delay?

7) Would you recommend any referrals or further evaluations?

**Case 2: Developmental Delays**

**Follow-up Visit #1 (Clinic Visit)**

Recap: Jackson saw you 6 months ago for his 24mo well visit, at which time his family had concern for a speech delay. You noted some social concerns as well, and he failed the ASQ communication section, scored in the gray for the personal/social and fine motor sections, and missed 5 questions on the MCHAT-R/F. You referred him to developmental and behavioral pediatrics and Early Interventions, and you discussed referrals to speech and occupational therapy although family wanted to hold off.

Jackson is now 30 months old, and he is presenting for a follow-up. He is waiting to be seen by Developmental-Behavioral Pediatrics but should have an initial evaluation in about 5 weeks.

Jackson has been enrolled in Early Interventions, and he has made some mild progress with his language development (he now has about 15 words with specific meaning). However, his mother is still concerned about his social skills. He still does not often respond to his name, and he still tends to get very fixated on his play. He is not lining up toys as much, but he still will spin the wheels on trucks and cars. He also does not seem interested in broadening his play to other toys or objects. He is now in day care but does not seem very interested in the other children. He is generally well-behaved, but there are times at day care when he throws large tantrums when he does not get what he wants, and he can sometimes get physical with others, mostly hitting with his hands.

1. What else would you like to know from the history?

On your exam, Jackson again seems aloof. He does not respond to his name and he does not make good eye contact. He keeps to himself and is playing with his toy cars on the floor, lining them up and crashing them together. His facial expression seems flat and every once in a while he flaps his hands and bounces up and down after crashing his cars. You do not hear any clear language, but you do hear frequent guttural sounds and repetitive throaty clicking noises as he plays. Jackson’s mother is interested in the evaluation with Developmental-Behavioral Pediatrics next month, but she wants to know if there is anything else she could be doing. She has heard of ABA and play-based treatments for children with autism, and she wants to know if you think these are things that would be available to Jackson.

1. What do you know about ABA and other treatment modalities for autism? How comfortable would you feel discussing these with Jackson’s mother?
2. What other supports or services that may be beneficial for Jackson?
3. If Jackson does end up being diagnosed with autism next month, what are some other considerations you may think about in the future with this child and family?
