# Supplementary material for: A Case-Based, Longitudinal Curriculum in Pediatric Behavioral and Mental Health
Source: MedEdPORTAL. 2024 Apr 29;20:11400. doi: 10.15766/mep_2374-8265.11400 (PMC11056487; doi:10.15766/mep_2374-8265.11400)
Supplement: Supplementary file 1 — Preteen Anxiety Case - Residents.docxPreteen Anxiety Case - Faculty Guide.docxPreteen Anxiety Case - SCARED Forms.pdfAnxiety Resources Handout.docxASD Delays Case - Residents.docxASD Delays Case - Faculty Guide.docxAutism Summary Handout and Resources.docxDepression Case - Residents.docxDepression Case - Faculty Guide.docxDepression Resources Handout.docxSchool-age ADHD Case - Residents.docxSchool-age ADHD Case - Faculty Guide.docxSchool-age ADHD Case - Vanderbilts.pdfADHD Handout.docxYoung ADHD and Behavior Case - Residents.docxYoung ADHD and Behavior Case - Faculty Guide.docxParenting Handout and Resource Sheet.docxBehavioral and Mental Health Curriculum Survey.docxBehavioral and Mental Health Pre-Post Test.docx [file mep_2374-8265.11400-s001.zip › F. ASD Delays Case - Faculty Guide.docx]

**Case 2: Developmental Delay and Autism**

**Learning Objectives**

By the end of the initial and follow-up cases, learners will be able to:

1. Explain the difference between developmental screening and surveillance
2. Score and interpret common screening tools for developmental delays and autism, including the ASQ and MCHAT-R/F
3. Identify historical and clinical features consistent with autism spectrum disorders in accordance with DSM-V criteria
4. Explain services and supports that you would recommend for a child who has delayed milestones
5. Identify when to refer a young child with developmental concerns for further evaluation

**Initial Visit**

CC: speech delay; wellness check

Jackson is a 24-month-old boy who presents with his mother for a 2-year wellness check and to discuss concerns about his speech and language development. He has generally been healthy, and per his mother he seems to be developing appropriately with the exception of his expressive language skills. She thinks he understands language better than he is able to speak it, and she says that he will sometimes gesture to his nose and head when you ask him to locate those body parts. Still, he does not always respond to his name and his ability to carry out commands (even simple one-step commands) is variable. Physically, he appears to be meeting all of his gross motor milestones on time, and he is learning to do more things independently.

1) What more information from the history would you like?

*Residents may ask about specific words that Jackson uses, size of vocabulary, and understandability. They may ask if he uses signs or other gestures.*

- *He only has about 5-10 words with specific meaning, and even then the words aren’t clear to people who don’t know him well.*
- *Examples of words he uses are “ma” for mom, “da” for dad (though he also uses “da” indiscriminately at other times too), “ba” for cup/bottle, and “tuh” for truck or car.*
- *He does not have any giant words and he struggles with common words and phrases such as please and thank you.*
- *He will wave bye-bye when prompted, but he does not always do this reliably. Otherwise, he does not use any signs and gestures are limited.*

*Residents may also ask about play skills and behaviors.*

- *Seems to prefer keeping to himself; doesn’t interact much with peers in any environment though parents note that he has not had too much exposure to other children*
- *Will play with preferred toys, but likes to line up toys and objects. He also sometimes plays with toys unusually (like spinning the wheels of a car close to his eyes or banging/stacking toys instead of playing with them). He will get upset if his line ups of toys are interrupted*
- *Generally does okay with transitions; he may occasionally get upset when something is taken from him but this is not a major issue*
- *Seems to get easily fixated on certain toys or objects; loves cars and trucks and won’t play with many other toys or objects*
- *Often flaps his hands, jumps, or wiggles his fingers close to his eyes if he is excited. He doesn’t often share excitement with others*
- *He does not seem to have many sensory differences; he does not like to have his teeth brushed, but it’s not too much of a battle. Loud noises and textures don’t bother him too much, though he is a very picky eater who seems to strongly prefer crunchy and bland foods.*

*Other historical data that may be discussed if asked (don’t take too much time here):*

*PREGNANCY/BIRTH HISTORY:*

*Jackson was born at 41+5 weeks EGA, and he was average for gestational age. The pregnancy was complicated by gestational diabetes, which was well-managed. The delivery was by C-section due to failure to progress and non-reassuring fetal heart tones. APGARs were 8 and 9, and he was vigorous after birth. He was a little sluggish with feeding at first, but he learned how to breastfeed quickly. He is described as being a very calm and relaxed baby. His newborn hearing screen and PKU were normal.*

*PAST MEDICAL HISTORY:*

*Jackson is generally healthy. He had croup last winter but otherwise has not been sick.*

*PAST SURGICAL HISTORY:*

*None reported.*

*DEVELOPMENTAL HISTORY:*

*18-month ASQ:*

*Communication: 20 (gray)*

*Gross Motor: 50*

*Fine Motor: 45*

*Problem Solving: 45*

*Personal/Social: 35 (gray)*

*18-month MCHAT:*

*1 question failed (#7)*

*Diet History: Jackson is a picky eater, and he has strong preferences for “brown and crunchy” foods like chips, French fries, crackers, and chicken nuggets. He eats some fruit (mostly bananas and apples, though he prefers apple sauce to apple slices), and he does not like vegetables unless they are pureed and mixed with something else. He likes milk and will drink quite a bit of it every day. He drinks juice and other sugary drinks a few times per week. He also drinks water.*

*Elimination History: Jackson takes Miralax as needed for bowel regularity. Currently, he takes it about 2-3 times per week, and he has a bowel movement most days.*

*FAMILY HISTORY:*

*Jackson’s father was a “late talker.” No other significant family history is reported.*

*SOCIAL HISTORY:*

*Jackson is an only child. His mother stays at home and his father is an active duty mechanic. They do not have any family in this area, and they moved here 6 months ago from Washington, D.C. The family denies any significant stressors in their household.*

2) How would you go about evaluating development in this 2-year-old?

*Screening tools and developmental surveillance. Think about ASQ and MCHAT-R.*

*Results included here; will have visual ASQ and MCHAT scores in the printout.*

*24-month ASQ:*

*Communication: 10 (black)*

*Gross Motor: 50*

*Fine Motor: 40 (gray)*

*Problem Solving: 40*

*Personal/Social: 35 (gray)*

*24-month MCHAT:*

*6 missed questions (#s 3, 6, 7, 8, 11, 14)*

3) What do you think about the scores on the screening tools above?

*Concerns for communication, fine motor, and personal-social skills based on ASQ.*

*They should comment on the 6 missed MCHAT questions. The next step is to administer the follow-up questions. Review that the follow-up questions are designed to be administered in person or over the phone, not by the family on their own as a handout.*

*Pull up the MCHAT-R and MCHAT-R_F (follow up algorithms) at:*

[*https://mchatscreen.com/wp-content/uploads/2015/09/M-CHAT-R_F_Rev_Aug2018.pdf*](https://mchatscreen.com/wp-content/uploads/2015/09/M-CHAT-R_F_Rev_Aug2018.pdf)

*We cannot reprint them due to copyrighting concerns, but they are free and open access for use in pediatric offices from the website above.*

*Run through 1-2 follow up question algorithms with the learners. After the follow-up questions are administered, 5 questions are still failed (only passes #3).*

4) Your physical exam is normal with the exception of his head appearing rather large but symmetric (when you measure it, it is 51cm – the 95^th^ percentile). What findings might you look for during your developmental surveillance to help you better understand this child’s development?

*Provide the information below when the residents prompt you to:*

- *Minimal eye contact present*
- *Response to name not present*
- *Child seemed to be “in his own world,” and would keep to himself*
- *No clear language heard, but some repetitive monosyllabic/guttural noises heard*
- *No scripting or immediate echolalia*
- *Playing with toy cars and trucks that he brought from home. Would roll them around but would also pick them up, turn them upside-down, and spin the wheels. Also had a line of objects from the exam room lined up on the exam table*
- *Cried and seemed agitated when you tried to perform your physical exam, and did not appear to respond to mom’s comforting*
- *Did not point or gesture at anything, and did not follow your point (even when you blew bubbles for him)*

5) What is your differential diagnosis for this child?

*Broad differential includes:*

- *Isolated speech and language delay*
- *Global developmental delay (including language delay)/intellectual disability*
- *Autism Spectrum Disorder*
- *Hearing loss*
- *Genetic syndrome associated with delayed development*

6) What features in this child are concerning for autism? Is there anything that reassures you against autism? How would you delineate autism versus a developmental delay?

*Encourage residents to think about screening tools if they don’t bring these up and only mention the physical findings.*

*Concerning?*

- *Poor eye contact*
- *Poor response to name*
- *Repetitive language/guttural noises; no clear language with communicative intent*
- *Deficient nonverbal skills (poor pointing, etc.)*
- *Restricted play skills (lining up toys, unusual play, etc.)*
- *Poor joint attention and interaction with others*
- *Does not interact much with peers*
- *Decreased emotional reciprocity (including empathy)*
- *MCHAT-R/F score of 5 after follow-up questions*
- *Communication in black on the 24-month ASQ, Personal/Social and Fine Motor in gray on ASQ*

*Reassuring?*

- *Not much; does not have strong sensory differences, generally does well with transitions… ‘B’ criteria are not the strongest in him*

7) Would you recommend any referrals or further evaluations?

- *Early Interventions*
- *Referral to Developmental-Behavioral Pediatrics for further evaluation*
- *Discussing ST or OT for this patient – in this case, we will say family would prefer to hold off for now*
- *Consider discussing parenting support groups*

**Case 2: Developmental Delay and Autism**

**Follow-up Visit #1 (Clinic Visit)**

Recap: Jackson saw you 6 months ago for his 24mo well visit, at which time his family had concern for a speech delay. You noted some social concerns as well, and he failed the ASQ communication section, scored in the gray for the personal/social and fine motor sections, and missed 5 questions on the MCHAT-R/F. You referred him to developmental and behavioral pediatrics and Early Interventions, and you discussed referrals to speech and occupational therapy although family wanted to hold off.

Jackson is now 30 months old, and he is presenting for a follow-up. He is waiting to be seen by Developmental-Behavioral Pediatrics but should have an initial evaluation in about 5 weeks.

Jackson has been enrolled in Early Interventions, and he has made some mild progress with his language development (he now has about 15 words with specific meaning). However, his mother is still concerned about his social skills. He still does not often respond to his name, and he still tends to get very fixated on his play. He is not lining up toys as much, but he still will spin the wheels on trucks and cars. He also does not seem interested in broadening his play to other toys or objects. He is now in day care but does not seem very interested in the other children. He is generally well-behaved, but there are times at day care when he throws large tantrums when he does not get what he wants, and he can sometimes get physical with others, mostly hitting with his hands.

1. What else would you like to know from the history?

- *He does not initiate play with other children*
- *Eye contact is still minimal*
- *He still is not pointing to indicate requests or interests, and other gestures are still lacking*
- *Sometimes his tantrums with transitions are severe and can last more than 10-15 minutes*
- *He still makes repetitive guttural noises*
- *He sometimes will immediately echo things said to him*
- *He still does not appear to have many sensory differences; he does not like lots of textures of food, but otherwise his parents can’t think of any*
- *He will still flap his hands and play with his fingers by his face when he gets excited*

On your exam, Jackson again seems aloof. He does not respond to his name and he does not make good eye contact. He keeps to himself and is playing with his toy cars on the floor, lining them up and crashing them together. His facial expression seems flat and every once in a while he flaps his hands and bounces up and down after crashing his cars. You do not hear any clear language, but you do hear frequent guttural sounds and repetitive throaty clicking noises as he plays. Jackson’s mother is interested in the evaluation with Developmental-Behavioral Pediatrics next month, but she wants to know if there is anything else she could be doing. She has heard of ABA and play-based treatments for children with autism, and she wants to know if you think these are things that would be available to Jackson.

1. What do you know about ABA and other treatment modalities for autism? How comfortable would you feel discussing these with Jackson’s mother?

*Ask residents to discuss what they know about ABA, the PLAY Project, and other autism-specific therapies. The goal is to help the residents have a baseline understanding of what ABA is, the rationale behind early and intensive treatment, and the strengths/weaknesses of different kinds of therapy approaches.*

1. What other supports or services that may be beneficial for Jackson?

*We want them to be thinking about educational resources – IEP evaluation when he turns 3 years old, in particular. Also, it is very reasonable for them to be thinking about speech therapy, occupational therapy, or other medically-based therapies if they haven’t discussed these already.*

1. If Jackson does end up being diagnosed with autism next month, what are some other considerations you may think about in the future with this child and family?

*We would want them to be thinking about not only ABA and other therapies, but also school-based services and supports. Additionally, we would want them to be able to discuss services and support networks like Autism Speaks. Genetic testing is also a recommendation for children diagnosed with autism, particularly testing for Fragile X Syndrome and either exome sequencing or a microarray. Consider screening for co-morbidities such as ADHD, learning disability/intellectual disability and other behavioral problems such as feeding difficulties, sleep problems, aggression, elopement, etc.*

**Additional Resources:**

Applied Behavioral Analysis: Autism Speaks. Available at: <https://www.autismspeaks.org/applied-behavior-analysis>. Accessed 03 Sep 22.

Autism Speaks. Available at: <https://www.autismspeaks.org/>. Accessed 03 Sep 22.

Centers for Disease Control and Prevention. Autism Spectrum Disorder. Available at: <https://www.cdc.gov/ncbddd/autism/index.html>. Accessed at 03 Sep 22.

Centers for Disease Control and Prevention. Autism Case Training Landing Page. Available at: <https://www.cdc.gov/ncbddd/actearly/act.html>. Accessed at 03 Sep 22.

Hyman SL, Levy SE, Myers SM. Identification, evaluation, and management of children with autism spectrum disorder. American Academy of Pediatric Council on Children with Disabilities, Section on Developmental and Behavioral Pediatrics. *Pediatrics*. 2020;145(1):e20193447.
