# Supplementary material for: A Case-Based, Longitudinal Curriculum in Pediatric Behavioral and Mental Health
Source: MedEdPORTAL. 2024 Apr 29;20:11400. doi: 10.15766/mep_2374-8265.11400 (PMC11056487; doi:10.15766/mep_2374-8265.11400)
Supplement: Supplementary file 1 — Preteen Anxiety Case - Residents.docxPreteen Anxiety Case - Faculty Guide.docxPreteen Anxiety Case - SCARED Forms.pdfAnxiety Resources Handout.docxASD Delays Case - Residents.docxASD Delays Case - Faculty Guide.docxAutism Summary Handout and Resources.docxDepression Case - Residents.docxDepression Case - Faculty Guide.docxDepression Resources Handout.docxSchool-age ADHD Case - Residents.docxSchool-age ADHD Case - Faculty Guide.docxSchool-age ADHD Case - Vanderbilts.pdfADHD Handout.docxYoung ADHD and Behavior Case - Residents.docxYoung ADHD and Behavior Case - Faculty Guide.docxParenting Handout and Resource Sheet.docxBehavioral and Mental Health Curriculum Survey.docxBehavioral and Mental Health Pre-Post Test.docx [file mep_2374-8265.11400-s001.zip › H. Depression Case - Residents.docx]

**Case 4**

**Initial Visit:**

Chris is a 15-year-old boy presenting with his parents for sad mood and declining school performance. His parents feel like he has not been himself the past few months. Despite their attempts to help, it seems like things are getting worse. They are frustrated that his grades have been declining and he is not completing his chores, resulting in frequent arguments at home. Chris admits that he has felt more down and irritable the past few months without any clear reasons. He reports decreased interest in playing basketball and isn’t as interested in spending time with his friends. He also reports trouble sleeping, with difficulty in falling asleep and staying asleep most nights.

1. What is your differential diagnosis thus far?
2. What are some potential screening tools you could use?
3. What additional information would you like from the family and/or patient?
4. What elements of your physical/mental status exam will you conduct?
5. What labs might you order and why? What are some Potential Medical Causes of Depression Sx’s?
6. What is your current assessment and treatment plan?
7. Which SSRI might you choose, and why?
8. What degree of safety assessment and possible safety plan might you use in this situation?

SSRI Chart for Reference:


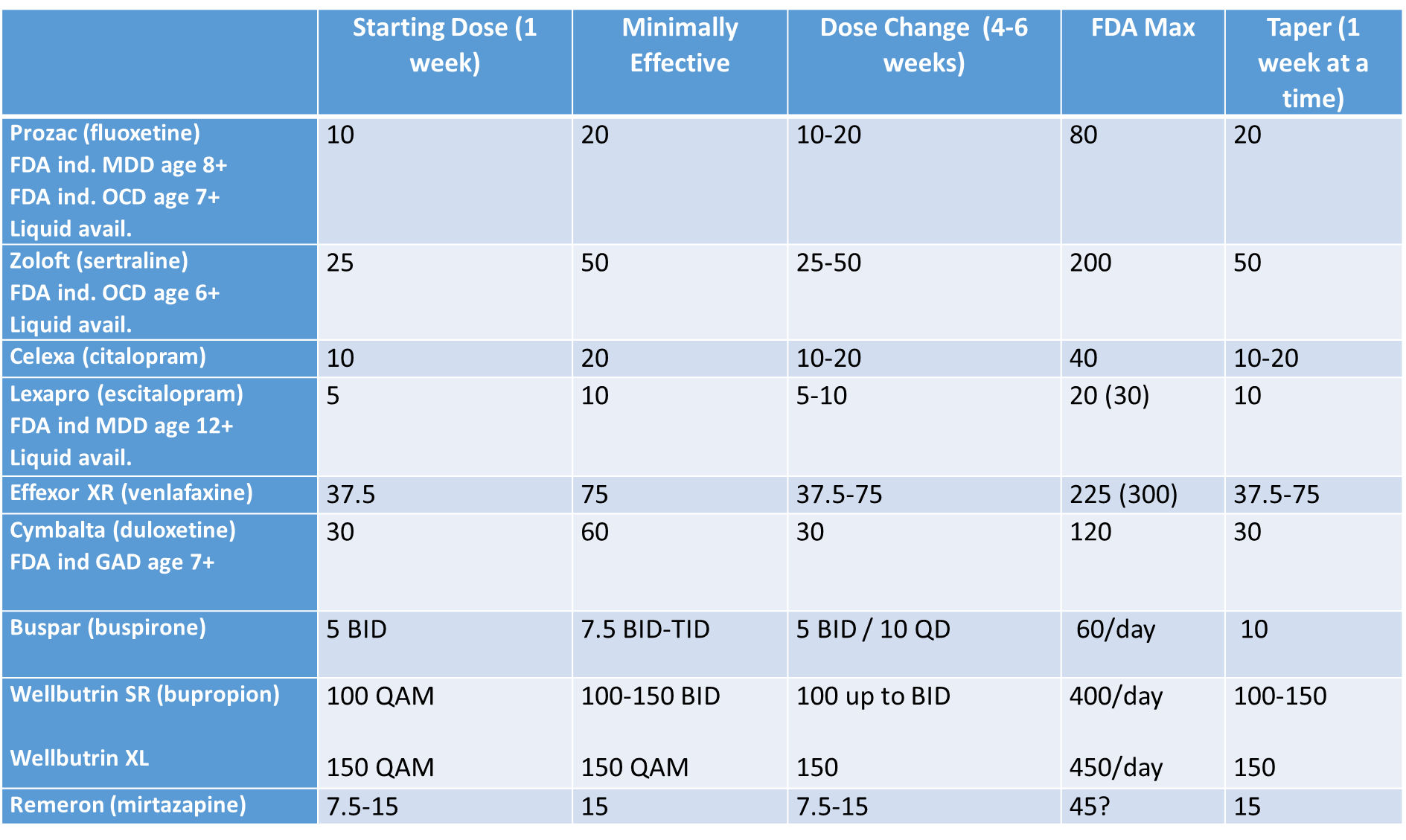


Author owned

SSRI: R/B/A’s

- **Benefits:** modulates serotonin in the brain/body, expected to improve mood in approximately 4-6wks at a particular dose.
- **Common side effects:** including GI, HA, drowsiness or activation, sexual SEs
- **Serious adverse effects:** including agitation, mania, suicidal thoughts and behaviors.
- **Black box warning (<24yo):** the potential for increased anxiety, energy, activation, agitation, suicidal ideations and suicidal behaviors, and close monitoring guidelines based on AACAP & FDA recommendations, to ensure the patient’s safety.
- **Alternatives:** multiple other SSRI’s, atypical antidepressants, individual psychotherapy

**Case 4: Depression**

**Follow-up Visit #1 (Phone Visit)**

Recap: Chris is a 15yo boy seen two weeks ago in clinic and diagnosed with major depressive disorder, single episode, moderate, without psychotic features. You referred him for counseling and started him on Zoloft 25mg daily. There were no safety concerns at that visit.

Chris’ parents report some improvement in his attitude after about a week of taking the medication. Chris doesn’t feel like the medication is working, but he denies current SI plan or intent. There have been no safety concerns from the family’s perspective. He initially had an upset stomach with mild nausea and loose stools that has improved the past few days. Chris still has some slight residual nausea in the morning for a couple hours after taking the medication. His parents are unsure if they should try a different medication given the GI side effects. They have scheduled an appointment for therapy in a few weeks.

1. What additional information you would like to request?
2. What is your plan given the patient’s complaints and concerns that the medication isn’t working?

**Case 4: Depression**

**Follow-up Visit #2 (Clinic Visit)**

Recap: Chris is a 15yoM with MDD who was started on Zoloft and increased from 25mg to 50mg PO daily and switched to bedtime dosing at a phone check-in 4wks ago.

History with parents in room:

Chris is reportedly adherent with current dose of Zoloft 50mg po daily with some mild improvements in mood. His parents claim that he has a better attitude towards them and seems to be make a better effort at trying to improve his grades. Since switching to evening dosing, Chris denies any medication side effects. He is still not sure if it is helping him much. He recently had an intake appointment for individual psychotherapy that he says went “OK.”

1. What additional information would you like?
2. What do you feel is necessary to disclose to his parents? Why or why not? How might you approach this conversation with the patient and his parents?
3. What are some of the factors relevant for evaluating patient safety regarding self-harm/suicide?
4. What are some important clinical steps for addressing the patient’s reported substance use?
5. What are important safety measures relevant for developing a patient safety plan?
6. What is your treatment plan at this time?

**Case 4: Depression**

**Follow-up Visit #3 (Clinic Visit)**

Recap: Chris is a 15yoM with MDD, who was started on Zoloft and increased from 50mg to 100mg po qhs at last appointment with you six months ago, as well as encouraged to stop cannabis/nicotine/caffeine intake and continue individual psychotherapy.

Chart Review:

Chris and his parents have presented to a colleague in the clinic for a couple times and has been titrated up to max dose of Zoloft 200mg po qhs for MDD to attempt to address residual symptoms. He has reportedly been adherent with medication, cut back on substances, and still participating in individual psychotherapy.

Patient and Parent Report:

Chris and his parents confirm that he has been adherent with Zoloft 200mg po qhs for over the past month without side effects. He admits that the medication has improved his overall mood and no longer has frequent thoughts about wishing he was dead, but his motivation and fatigue remain low and he still has low mood more days than not that causes issues with his school performance and social life. He is still participating in individual psychotherapy and likes his therapist.

When interviewed alone, Chris confirms what he has already shared, and denies any MI/SI/HI/AVH/self-harm. He is proud to report he has stopped smoking marijuana.

1. What additional information would you like?
2. What are your next steps for treating this patient’s residual depression?
