# Supplementary material for: A Case-Based, Longitudinal Curriculum in Pediatric Behavioral and Mental Health
Source: MedEdPORTAL. 2024 Apr 29;20:11400. doi: 10.15766/mep_2374-8265.11400 (PMC11056487; doi:10.15766/mep_2374-8265.11400)
Supplement: Supplementary file 1 — Preteen Anxiety Case - Residents.docxPreteen Anxiety Case - Faculty Guide.docxPreteen Anxiety Case - SCARED Forms.pdfAnxiety Resources Handout.docxASD Delays Case - Residents.docxASD Delays Case - Faculty Guide.docxAutism Summary Handout and Resources.docxDepression Case - Residents.docxDepression Case - Faculty Guide.docxDepression Resources Handout.docxSchool-age ADHD Case - Residents.docxSchool-age ADHD Case - Faculty Guide.docxSchool-age ADHD Case - Vanderbilts.pdfADHD Handout.docxYoung ADHD and Behavior Case - Residents.docxYoung ADHD and Behavior Case - Faculty Guide.docxParenting Handout and Resource Sheet.docxBehavioral and Mental Health Curriculum Survey.docxBehavioral and Mental Health Pre-Post Test.docx [file mep_2374-8265.11400-s001.zip › I. Depression Case - Faculty Guide.docx]

**Case 4: Depression**

**Learning Objectives:**

By the end of the initial and follow-up cases, learners will be able to:

1. Identify symptoms of major depressive disorder in adolescents in accordance with DSM-V criteria
2. Develop a comprehensive differential diagnosis for low mood in adolescents
3. Apply evidence-based best practices for managing depression with antidepressants to simulated scenarios
4. Develop a comprehensive patient safety assessment and plan for patients at risk for suicidality

**Initial Visit:**

Chris is a 15yo boy presenting with his parents for sad mood and declining school performance. His parents feel like he has not been himself the past few months. Despite their attempts to help, it seems like things are getting worse. They are frustrated that his grades have been declining and he is not completing his chores, resulting in frequent arguments at home. Chris admits that he has felt more down and irritable the past few months without any clear reasons. He reports decreased interest in playing basketball and isn’t as interested in spending time with his friends. He also reports trouble sleeping, with difficulty in falling asleep and staying asleep most nights.

1. **What is your differential diagnosis thus far?**

- *Major Depressive Disorder*
- *Generalized Anxiety disorder*
- *Adjustment Disorder*
- *Trauma-related disorder*
- *Insomnia*
- *Substance use disorder*
- *Bipolar disorder*
- *Prodromal Psychotic disorder*
- *Other medical conditions*

1. **What are some potential screening tools you could use?**

- *PHQ-9A: Depression*
- *CSSRS: Suicide Risk Assessment*
- *GAD-7: Anxiety*
- *CYBOC: OCD*
- *CRAFFT: adolescent substance use*

*For this case, you administer the PHQ-9A, and Chris scores 17 (0 on #9).*

*Ask how the learners interpret this finding*

*Answer: 0-4 normal/mild, 5-9 mild depression, 10-14 moderate depression, 15+ severe depression.*

1. **What additional information would you like from the family and/or patient?**

*Chris’ parents are worried about his change in mood/behavior but are not currently concerned about his safety. They deny any other mental health or medical concerns on ROS. They deny history of persistently elevated or irritable moods. They deny any past attempts at self-harm or suicide. Patient has no significant medical history; NKDA; no past psychiatric treatment (no medications, hospitalizations, therapy, etc.). There is a maternal family history of Major depressive disorder; possible history of bipolar disorder and substance use disorder in mother’s uncle.*

*Discussion with patient alone:*

*Chris and his parents agree to individual interview time without his parents present. Limits of confidentiality were reviewed with patient and his parents. Chris is slow to open up, mostly giving only one-word answers at first. He denied current romantic relationships or sexual activity, denied any substance use other than one can of soda per day, and he denied self-harm or past suicide attempts. Chris is open to trying medications if others think it could help. He is reluctant to talk with someone about his feelings in individual psychotherapy because he claims he has nothing to talk about.*

1. **What elements of your physical/mental status exam will you conduct?**

*Vitals:*

*BP: 104/66*

*HR: 95*

*RR: 12*

*T: 98 °F*

*HT: 65’’*

*WT: 125lbs*

*BMI 20.8*

*Neurologic exam is normal. No thyromegaly/goiter. No pallor or other skin changes/rashes.*

*Mental Status Exam:*

*Appearance: Appears grossly alert and oriented, appears stated age, casually dressed, some below average hygiene, messy hair doesn’t appear to have showered in past 1-2 days.*

*Behavior: Guarded, slow to warm. Slightly decreased eye contact. Patient/caregiver interaction: developmentally appropriate*

*Motor: no motor abnormalities, gait normal, no PMR/PMA*

*Speech: Normal rate, rhythm, volume; fairly monotone. Non-pressured, fluent.*

*Mood: “I don’t know”*

*Affect: Constricted, dysphoric and irritable at times, mood mostly congruent, non-labile.*

*Thought Process: Coherent, linear, logical and goal-directed. No flight of ideas or loose associations.*

*Thought Content: Denies current SI/HI/AVH. Reports frequent MI and has had several periods of SI with plan but without intent or access. No delusions/paranoia. No RTIS. +Self-deprecating thoughts.*

*Intellect/Memory: Estimated as average based on interview. Immediate, recent, and remote all intact on gross examination. Attention/concentration: fair*

*Judgment/Insight: Fair, age-appropriate*

1. **What labs might you order and why? What are some Potential Medical Causes of Depression Sx’s?**

***Labs:***

- *Limited evidence for lab screening in absence of other physical symptoms or medical co-morbidities*
- *Routine labs: CBC, BMP, TSH, urinalysis, urine pregnancy test, urine drug screen*
- *Evidence with specific symptoms:*
  - *Iron/ferritin*
  - *Vitamin B12/folate*
  - *Vitamin D*
- *Limited clinical evidence for pharmacogenetic testing outside of complicated medical co-morbidities or polypharmacy*

***Potential medical causes:***

- *Anemia (Iron deficiency anemia)*
- *Possible influence from Vitamin D deficiency*
- *Vitamin B12 insufficiency*
- *Diabetes*
- *Cancer*
- *Adrenal insufficiency*
- *Hypercortisolism*
- *Hypothyroidism*
- *Mononucleosis*
- *Systemic lupus erythematosus*
- *Obstructive sleep apnea*
- *Traumatic brain injury*
- *Stroke*
- *Neurodegenerative diseases*

1. **What is your current assessment and treatment plan?**

*Chris is a 15yo boys with symptoms c/w major depressive disorder, single episode, moderate, without psychotic features. Due to the severity of this symptoms, he would benefit from starting an SSRI while simultaneously seeking individual psychotherapy for depression.*

1. **Which SSRI might you choose, and why?**

*Considerations for selection:*

- *Patient’s past meds*
  - *Beneficial and harmful medication reactions*
  - *Adherence Hx*
- *Family history of treatment*
  - *MDD with response to a particular medication*
  - *Bipolar disorder with SSRI induced mania*
- *Prominent depressive symptoms*
  - *Insomnia*
  - *Poor appetite*
  - *Avolition*
  - *Fatigue*
- *Comorbid mental health issues*
  - *ADHD*
  - *Anxiety*
  - *Bipolar disorder*
  - *Etc.*
- *Complicated medical conditions*


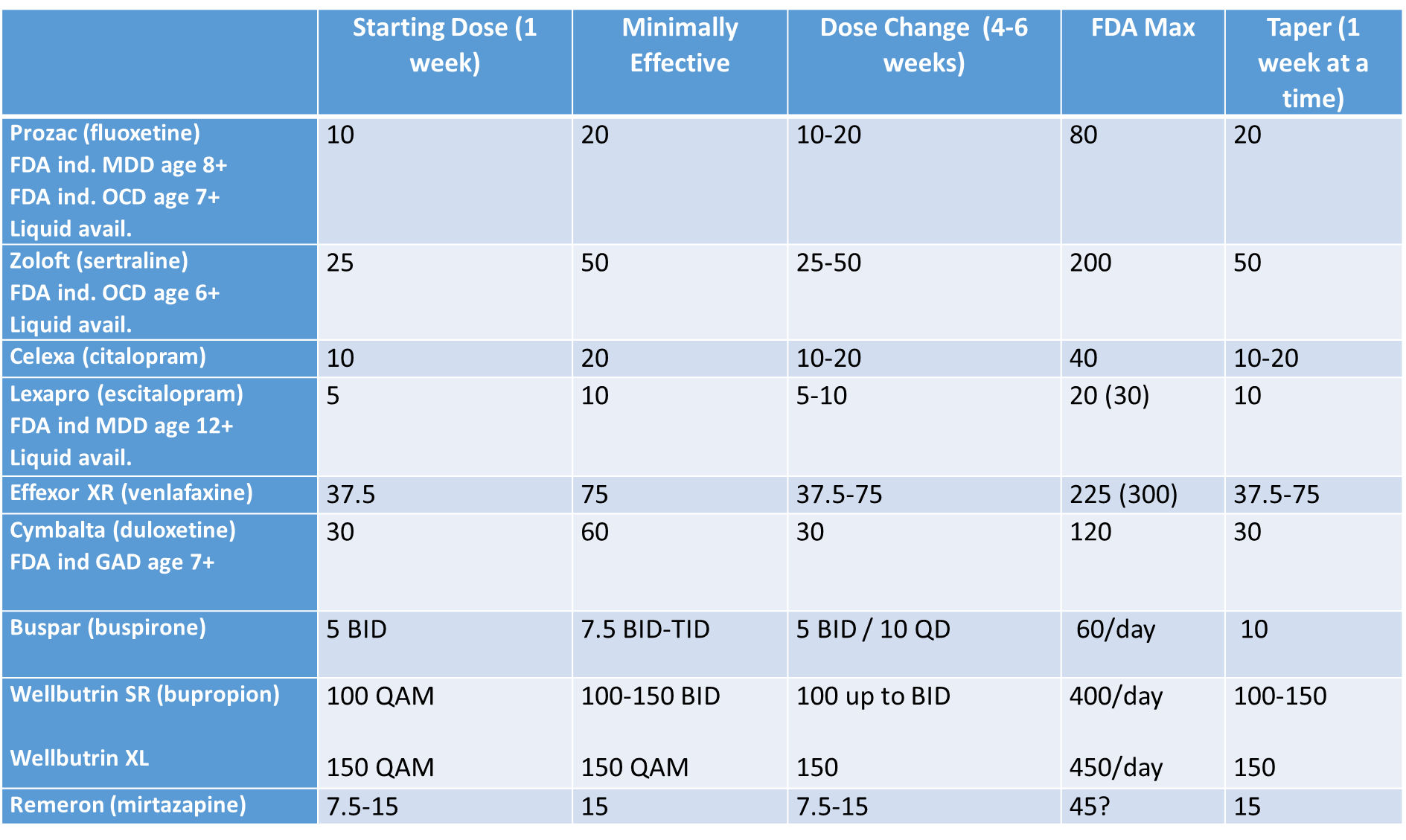


Author Owned

SSRI: R/B/A’s (this section does appear on resident handout)

- **Benefits:** modulates serotonin in the brain/body, expected to improve mood in approximately 4-6wks at a particular dose.
- **Common side effects:** including GI, HA, drowsiness or activation, sexual SEs
- **Serious adverse effects:** including agitation, mania, suicidal thoughts and behaviors.
- **Black box warning (<24yo):** the potential for increased anxiety, energy, activation, agitation, suicidal ideations and suicidal behaviors, and close monitoring guidelines based on AACAP & FDA recommendations, to ensure the patient’s safety.
- **Alternatives:** multiple other SSRI’s, atypical antidepressants, individual psychotherapy

Plan:

- Start Zoloft 25mg po daily for MDD
- Referral to individual psychotherapy
- Labs ordered: CBC, BMP, TSH
- Repeat PHQ-9A prior to follow-up
- Follow-up in 1-2wks via telephone regarding response and tolerance of potential side effects

1. **What degree of safety assessment and possible safety plan might you use in this situation?**

*No acute safety plan is formally established because he is not endorsing any suicidality or safety concerns, but due to current mood issues and starting SSRI, the black box warning for SI along with directions on how to connect with crisis hotline, EMS/911, or nearest ER if safety concerns develop remains important counseling.*

**Case 4: Depression**

**Follow-up Visit #1 (Phone Visit)**

Recap: Chris is a 15yo boy seen two weeks ago in clinic and diagnosed with major depressive disorder, single episode, moderate, without psychotic features. You referred him for counseling and started him on Zoloft 25mg daily. There were no safety concerns at that visit.

Chris’ parents report some improvement in his attitude after about a week of taking the medication. Chris doesn’t feel like the medication is working, but he denies current SI plan or intent. There have been no safety concerns from the family’s perspective. He initially had an upset stomach with mild nausea and loose stools that has improved the past few days. Chris still has some slight residual nausea in the morning for a couple hours after taking the medication. His parents are unsure if they should try a different medication given the GI side effects. They have scheduled an appointment for therapy in a few weeks.

1. **What additional information you would like to request?**

*PHQ-9A:*

*Initial Score: 17 (0 on #9)*

*Current Score: 15 (0 on #9)*

*Lab Results:*

*CBC: WBC 8.3, HGB 12.9, HCT 39.3, PLT 360*

*BMP: Na 140, K 4.1, Cl 106, CO2 27, BUN 8, Cr 0.6, Glu 108*

*TSH: 1.04*

*All these labs are normal and do not change your treatment plan. No further labs indicated today.*

1. **What is your plan given the patient’s complaints and concerns the medication isn’t working?**

- *Recommend Chris continue the medication, switch to evening dosing if tolerated, and increase Zoloft 25mg to 50mg po daily for MDD.*
  - *Reviewed R/B/A/SE with parents without new questions/concerns.*
- *Confirm individual psychotherapy has been established at next appointment.*
- *Follow-up scheduled in-person in one month*

**Case 4: Depression**

**Follow-up Visit #2 (Clinic Visit)**

Recap: Chris is a 15yoM with MDD who was started on Zoloft and increased from 25mg to 50mg PO daily and switched to bedtime dosing at a phone check-in 4wks ago.

History with parents in room:

Chris is reportedly adherent with current dose of Zoloft 50mg po daily with some mild improvements in mood. His parents claim that he has a better attitude towards them and seems to be make a better effort at trying to improve his grades. Since switching to evening dosing, Chris denies any medication side effects. He is still not sure if it is helping him much. He recently had an intake appointment for individual psychotherapy that he says went “OK.”

1. **What additional information would you like?**

*PHQ-9A:*

*Initial Score: 17 (0 on #9)*

*Current Score: 16 (0 on #9)*

*Discussion with patient alone:*

*Chris admits to having frequent thoughts of wishing he wouldn’t wake up or that he might be better off dead, occurring several days per week and lasting at least an hour before bed, that have been decreasing in frequency since starting mental health treatment. He has never tried to kill himself, but he has had fleeting thoughts about shooting himself in the past. Generally these thoughts concern him and he denies any intent to act on them. He doesn’t want to hurt his family and denies knowing where he would even get a gun as there are none in the home.*

*He reports his primary coping mechanism for dealing with these uncomfortable feelings has been smoking marijuana. Chris admits to smoking marijuana a couple times per week after school with a friend. He has a caffeinated soda 1-2x per day and vapes nicotine a few times per month. He denies all other substance use, denies any negative consequences to substance use, and denies any desire to change. He doesn’t want you to tell his parents about his substance use.*

1. **What do you feel is necessary to disclose to his parents? Why or why not? How might you approach this conversation with the patient and his parents?**

*Encourage patient to open a discussion with his parents about his suicidal thoughts and his concerns about these thoughts. You may help facilitate this discussion with him, but encourage him to take the lead. Also encourage the patient to consider discussing his substance use with his parents or imagine the discussion if they were to find out. It probably wouldn’t be required to break confidentiality for either the suicidal thoughts or substance use (depending on the level of risk you assign to the suicidal thoughts), but continue to encourage communication between patient and parents.*

1. **What are some of the factors relevant for evaluating patient safety regarding self-harm/suicide?**

*Protective factors that may point away from needing to break confidentiality at this time:*

- *Mostly morbid ideations without intent or plan to harm self*
- *Only fleeting thoughts of suicide with a plan, but no access or intent*
- *No past self-harm or suicide attempts*
- *Patient doesn’t want to emotionally hurt his family by attempting suicide*
- *Ego-dystonic thoughts (patient is disturbed by SI and doesn’t want to die)*

*In general:*

- *Static Factors:*
  - *Family history of suicide, prior personal suicide attempts, history of abuse*
  - *Male gender, specific age ranges (18-25)*
- *Dynamic Factors:*
  - *Mental illness especially depression, substance misuse/abuse, chronic medical condition*
  - *Hopelessness, impulsivity, isolation, loss*
  - *Access to lethal methods, barriers to accessing mental health treatment, *acute changes in treatment*
- *Protective Factors:*
  - *Access to/Adherence to evidence-based treatment*
  - *Help-seeking, Supportive relationships*

1. **What are some important clinical steps for addressing the patient’s reported substance use?**

*Substance use history in teenagers should be conducted without the parent/guardian present whenever possible. Substance use behaviors should be screened with an evidence-based tool for adolescents, such as the CRAFFT Screen. CRAFFT is an acronym for Car, Relax, Alone, Forget, Friends, Trouble (see full CRAFFT 2.1+N version attached below). A non-judgmental screening should include asking about an array of specific substances (alcohol, caffeine, tobacco/nicotine/vaping, THC/cannabinoids, hallucinogens, stimulants/cocaine/meth, opioids, etc) as well as “huffing” household items/chemicals and misuse of old household Rx’s. Motivational Interviewing techniques should be utilized to assess appropriate stage of change for any active reported substance use (Stages include Pre-contemplation, Contemplation, Preparation, Action, Maintenance). When there is clinical suspicion of substance use impacting mental or physical health, a urine drug screen (UDS) may be warranted.*


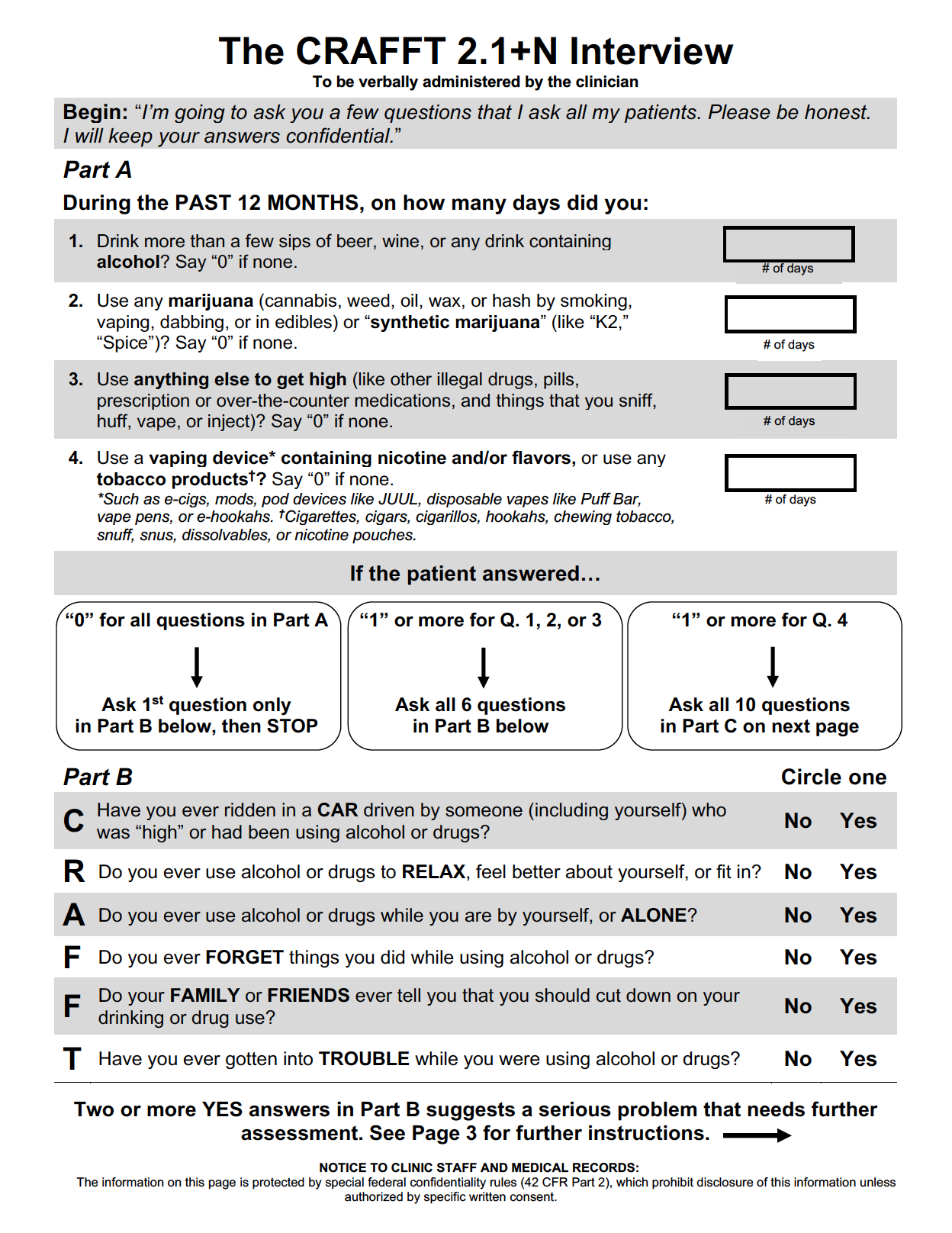


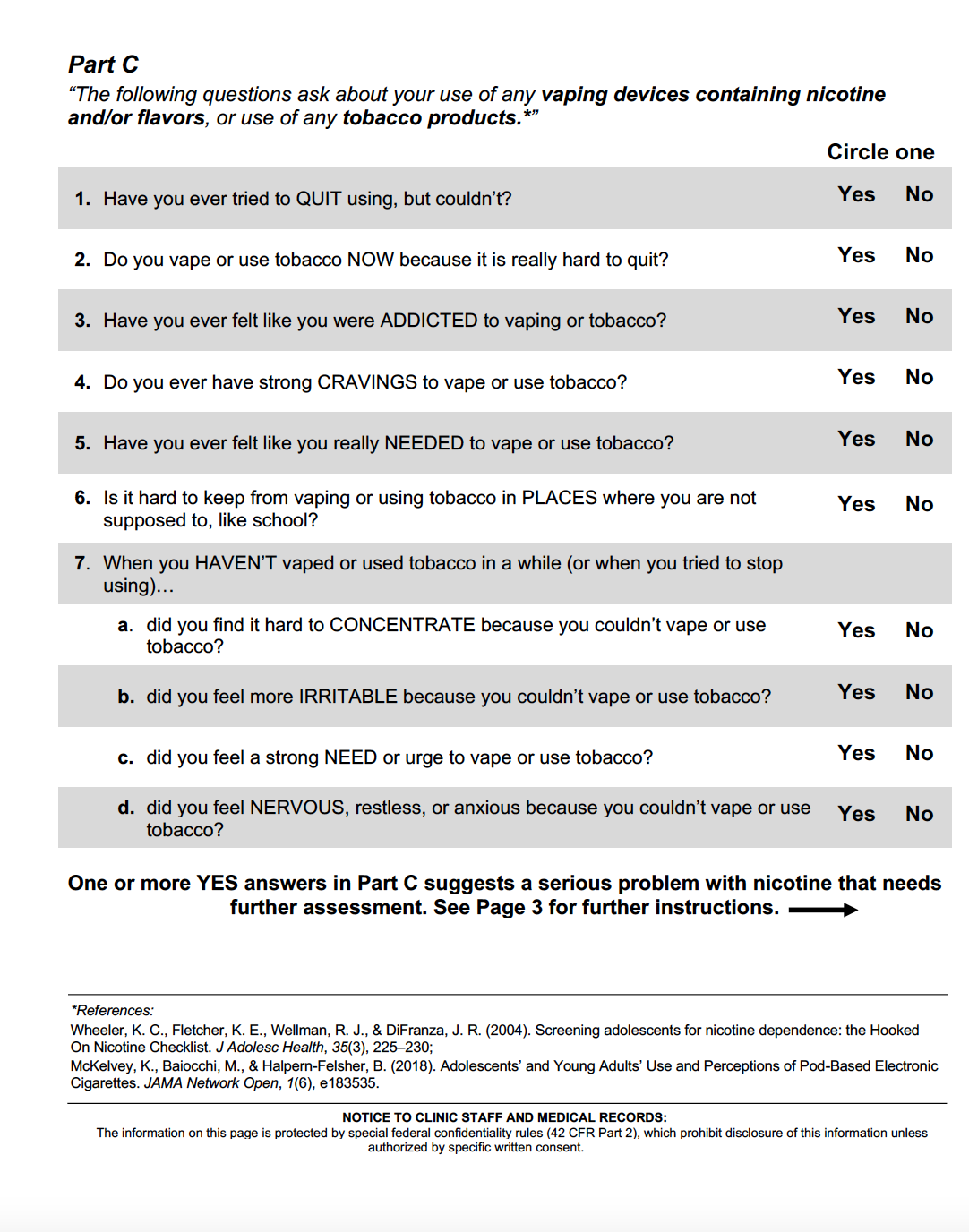


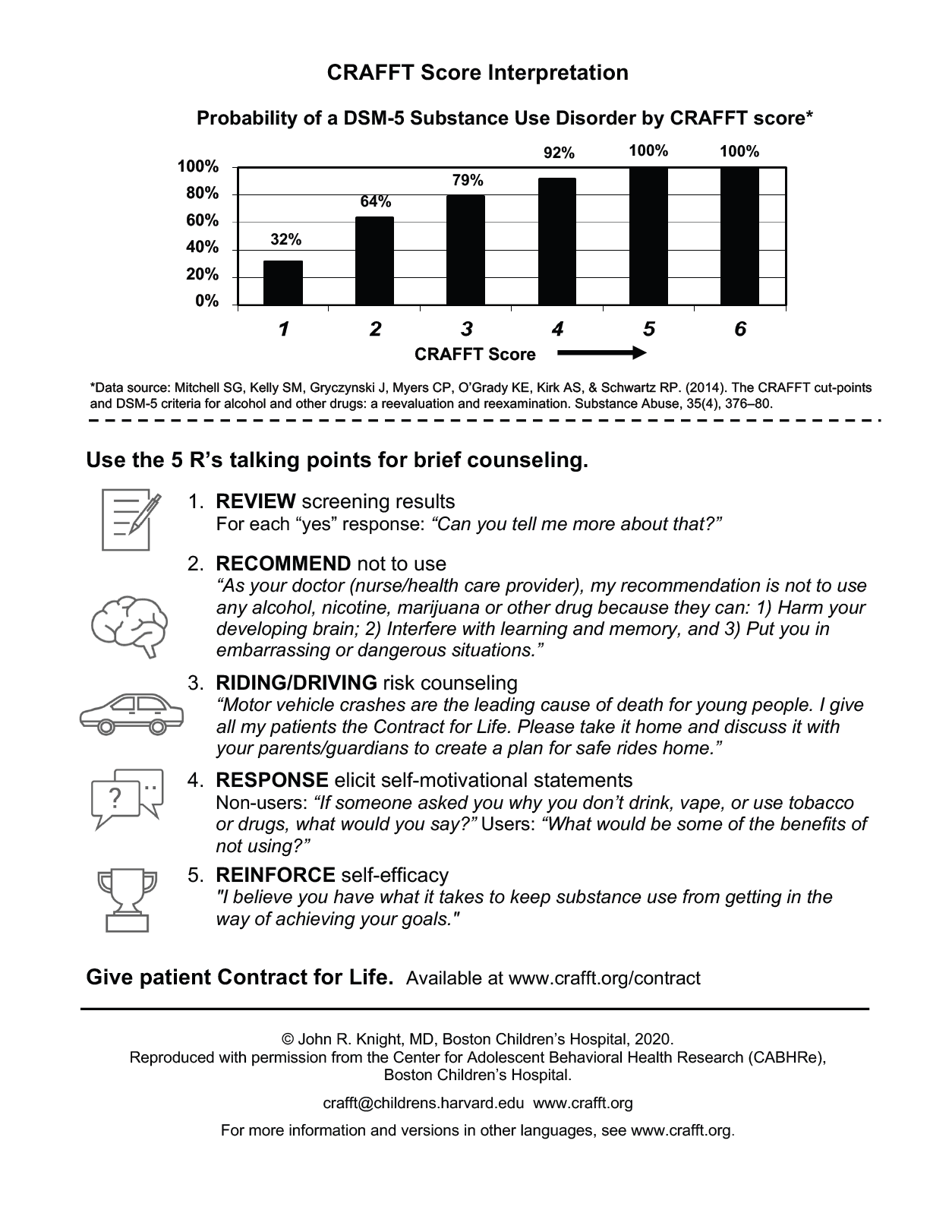


CRAFFT Copyrighted. Permission received from Julia Plumb on Mar 10, 2023.

*Vitals: within normal limits; weight is stable*

*Mental Status Exam: remarkable for the patient more open, engaged, and less irritable with interview. Affect remains dysphoric, constricted, non-labile. He admits to morbid ideations a few times per week, but denies current MI/SI/HI/AVH/self-harm.*

1. **What are important safety measures to take when developing a patient safety plan?**

*Removal of lethal means is probably one of the more critical immediate steps to take in safety planning*

- *Recommend removal of firearms*
- *Lock away ALL medications, including over-the-counters*
- *Lock away or remove sharps*
- *Consider checking for other means such as ligatures, dangerous chemicals*

*Reduction of significant and imminent stressors, or finding ways to manage these stressors*

- *Is social media supportive or are specific contacts detrimental?*
- *Management of academic stress*
- *Healthy distractions/coping when feeling distressed*

*Increased monitoring, avoid isolation*

- *Reduction or elimination of time spent alone*
- *Increasing time spent with supportive others, identify helpful friends/family to contact*

*Available help: treatment team, text 741741, call 1-800-273-8255, local ER*

*Safety Plan: A collaborative safety plan was developed with the patient and his parents. We have confirmed there is limited access to lethal means at home, and family is comfortable with continued outpatient management at this time.*

1. **What is your treatment plan at this time?**

- *Increase Zoloft 50mg to 100mg po qhs for MDD*
  - *Reviewed R/B/A/SE with parents without new questions/concerns.*
- *Encourage cessation from cannabis, nicotine, and slowly minimize caffeine intake*
  - *Use motivational interviewing techniques*
  - *Patient at pre-contemplative stage of change*
  - *Order UDS to screen for other substance use*
- *Continue weekly individual psychotherapy*
  - *Encouraged pt to discuss substance use with therapist*
- *Complete safety plan and review with patient/family*
- *Schedule for follow-up in one month*

**Case 4: Depression**

**Follow-up Visit #3 (Clinic Visit)**

Recap: Chris is a 15yoM with MDD, who was started on Zoloft and increased from 50mg to 100mg po qhs at last appointment with you six months ago, as well as encouraged to stop cannabis/nicotine/caffeine intake and continue individual psychotherapy.

Chart Review:

Chris and his parents have presented to a colleague in the clinic for a couple times and has been titrated up to max dose of Zoloft 200mg po qhs for MDD to attempt to address residual symptoms. He has reportedly been adherent with medication, cut back on substances, and still participating in individual psychotherapy.

Patient and Parent Report:

Chris and his parents confirm that he has been adherent with Zoloft 200mg po qhs for over the past month without side effects. He admits that the medication has improved his overall mood and no longer has frequent thoughts about wishing he was dead, but his motivation and fatigue remain low and he still has low mood more days than not that causes issues with his school performance and social life. He is still participating in individual psychotherapy and likes his therapist.

When interviewed alone, Chris confirms what he has already shared, and denies any MI/SI/HI/AVH/self-harm. He is proud to report he has stopped smoking marijuana.

1. **What additional information would you like?**

*PHQ-9A:*

*Initial Score: 17 (0 on #9)*

*Current Score: 10 (0 on #9)*

*Vitals: within normal limits; growth curves are unremarkable*

*Mental Status Exam: patient is polite and engaged in interview. Affect is mildly dysphoric, full-range, non-labile. He denies recent or current MI/SI/HI/AVH/self-harm. He is more future oriented for treatment and improving his grades to get into college. No self-deprecating thought content at this time.*

*Labs: prior UDS obtained just before this appointment was negative*

1. **What are your next steps for treating this patient’s residual depression?**

- *Treat to full remission of symptoms****
- *Try switching to another antidepressant:*
  - *Switching to a different SSRI can be effective for achieving remission (TORDIA) and usually best tolerated*
  - *Bupropion might help more specifically for anhedonia, poor motivation, fatigue*
- *Behavioral activation techniques:*
  - *Increasing activity levels to combat the use of unhealthy behaviors to avoid negative feelings*
  - *Examples include taking a walk, waking up at a reasonable time and scheduling an enjoyable activity in the morning, playing on a sports team, or hanging out with a friend – even if they don’t feel like it at the time!*
  - *Includes problem solving strategies to reduce barriers*
- *Confirm adherence to evidence-based treatments*
- *Reevaluate other potential causes of depression/stress/trauma*
