# Supplementary material for: A Case-Based, Longitudinal Curriculum in Pediatric Behavioral and Mental Health
Source: MedEdPORTAL. 2024 Apr 29;20:11400. doi: 10.15766/mep_2374-8265.11400 (PMC11056487; doi:10.15766/mep_2374-8265.11400)
Supplement: Supplementary file 1 — Preteen Anxiety Case - Residents.docxPreteen Anxiety Case - Faculty Guide.docxPreteen Anxiety Case - SCARED Forms.pdfAnxiety Resources Handout.docxASD Delays Case - Residents.docxASD Delays Case - Faculty Guide.docxAutism Summary Handout and Resources.docxDepression Case - Residents.docxDepression Case - Faculty Guide.docxDepression Resources Handout.docxSchool-age ADHD Case - Residents.docxSchool-age ADHD Case - Faculty Guide.docxSchool-age ADHD Case - Vanderbilts.pdfADHD Handout.docxYoung ADHD and Behavior Case - Residents.docxYoung ADHD and Behavior Case - Faculty Guide.docxParenting Handout and Resource Sheet.docxBehavioral and Mental Health Curriculum Survey.docxBehavioral and Mental Health Pre-Post Test.docx [file mep_2374-8265.11400-s001.zip › K. School-age ADHD Case - Residents.docx]

**Case 3**

**Initial Visit**

CC: school difficulty and inattention

Maria is a 7-year-old girl who presents with her parents due to concerns about school performance and focus. Maria is currently in the first grade and she seems to be behind her peers in most subjects. She is struggling with learning to read, and based on recent school standardized testing she was well below average in reading and writing. She has always done better at math, but she is still below average in that subject as well. Her parents report that her Kindergarten teacher had concerns about Maria’s learning, but she felt that she was making gradual progress. Now that she is in first grade, her teacher is more concerned. She has told her parents that Maria really seems to struggle with focus, and that it is hard for her to stay on task and complete assignments. She seems to work really slowly and “space out” when doing work independently. She also does not appear to listen to directions well, and she seems forgetful at school. She is generally calm and quiet, and she does not have any behavioral issues at school. Her parents have not been previously as concerned as her teachers, as they figured she was just a little bit behind and that she would catch up over time. But, seeing her recent standardized testing scores concerned them. They wonder if further evaluation would be helpful.

1) What more information from the history would you like?

Your physical examination is normal. You note that she is very quiet, but she will answer your questions appropriately. She makes good eye contact when speaking with you and she seems to be listening when you and her parents are talking. When you ask her how she likes school she says “I like it,” and when you ask her what her favorite part of school is she says, “Playing outside with my friends.” Neurologic exam is normal.

2) Are there any other physical findings/observations you would like to know about?

3) What is your differential diagnosis?

4) What is your plan for today? What can you recommend to her family?

**Case 3: School-Age ADHD**

**Follow-up Visit #1 (Clinic Visit)**

Recap: Maria is a 7yo girl who presented for inattention and learning difficulties. She has a family history of ADHD in her father, and maternal uncles. She is overall a happy and social child with good peer relationships. She does struggle with sleep initiation. At the last visit, you sent family home with Vanderbilt forms and discussed sleep hygiene.

One month after your initial visit with Maria and her parents, you have a follow-up appointment with them. During this visit, you discuss the Vanderbilt screen results, which showed the following (see handouts):

1) What do these results mean to you? How would you explain them to Maria’s parents?

2) What do you do with this information? Are there other screening tools you can use?

After discussing the results in detail with her parents, you inform them that you are diagnosing Maria with ADHD, primarily inattentive type. Her parents want to know more about this diagnosis and what it means for Maria going forward, specifically what the treatment options are.

3) Can you describe a treatment approach for Maria’s ADHD?

4) What are the medication options for Maria? What counseling would you provide regarding medication initiation?

5) Are there other non-medicinal options you would recommend for Maria?

6) What is your plan for Maria? If you start a medication which one would you choose?

**Case 3: School-Age ADHD**

**Follow-up Visit #2 (Virtual/Phone Visit)**

Recap: Maria is a 7yo girl you have diagnosed with ADHD and started on long-acting methylphenidate (Concerta) 18mg daily at her last visit.

Today is a one-month virtual follow-up since you last saw her. In the interim, Maria’s mother called the clinic because while the 18mg of long-acting methylphenidate (Concerta) was working somewhat, she was still having issues with focus and inattention at school, especially in the afternoon. Since she was not having significant side effects (only mild appetite suppression), one of your colleagues increased her from 18mg to 27mg daily of long-acting methylphenidate (Concerta) based on your previous titration plan (you were on leave when Maria’s mother called). Maria’s mother reports that Maria has been more focused and attentive at school since she has been on the 27mg of long-acting methylphenidate (Concerta).

1) What questions do you have for Maria’s mother regarding her stimulant therapy?

2) How do you respond to what Maria’s mother told you about the efficacy of the medication and the side effects that Maria is experiencing?

3) What is your approach to managing her stimulant regimen?

Maria’s mother agrees with you that even with the side effects experienced with long-acting methylphenidate (Concerta), it has been helpful for Maria. She was pleased with the benefit she experienced and hopeful that another stimulant might be a better fit for her. In spite of this, she remains concerned about Maria’s learning: “Even if we manage her ADHD symptoms, I am worried that she won’t be able to do well in school, especially with reading. Are there any other supports or interventions that could help Maria in school?”

4) What is your response to Maria’s mother? Are there other supports, educational or otherwise, that you may recommend to Maria’s mother?

5) What is your follow-up plan for Maria? How will you manage her ADHD going forward?
