# Supplementary material for: A Case-Based, Longitudinal Curriculum in Pediatric Behavioral and Mental Health
Source: MedEdPORTAL. 2024 Apr 29;20:11400. doi: 10.15766/mep_2374-8265.11400 (PMC11056487; doi:10.15766/mep_2374-8265.11400)
Supplement: Supplementary file 1 — Preteen Anxiety Case - Residents.docxPreteen Anxiety Case - Faculty Guide.docxPreteen Anxiety Case - SCARED Forms.pdfAnxiety Resources Handout.docxASD Delays Case - Residents.docxASD Delays Case - Faculty Guide.docxAutism Summary Handout and Resources.docxDepression Case - Residents.docxDepression Case - Faculty Guide.docxDepression Resources Handout.docxSchool-age ADHD Case - Residents.docxSchool-age ADHD Case - Faculty Guide.docxSchool-age ADHD Case - Vanderbilts.pdfADHD Handout.docxYoung ADHD and Behavior Case - Residents.docxYoung ADHD and Behavior Case - Faculty Guide.docxParenting Handout and Resource Sheet.docxBehavioral and Mental Health Curriculum Survey.docxBehavioral and Mental Health Pre-Post Test.docx [file mep_2374-8265.11400-s001.zip › L. School-age ADHD Case - Faculty Guide.docx]

**Case 3: Uncomplicated ADHD**

**Learning Objectives**

By the end of the initial and follow-up cases, learners will be able to:

1. Diagnose ADHD in accordance with DSM-V criteria in a school-age child
2. Build a differential diagnosis of at least five diagnoses for inattention and school difficulties in children
3. Describe behavioral and medicinal management approaches for children with ADHD, to include stimulant and non-stimulant medications
4. Explain at least six common side effects commonly associated with stimulant medication and how to counsel families about these side effects
5. Discuss school-based supports and services for children with ADHD and/or a learning disability, including IEPs and 504 plans

**Initial Visit**

CC: school difficulty and inattention

Maria is a 7-year-old girl who presents with her parents due to concerns about school performance and focus. Maria is currently in the first grade, and she seems to be behind her peers in most subjects. She is struggling with learning to read, and, based on recent school standardized testing, she was well below average in reading and writing. She has always done better at math, but she is still below average in that subject as well. Her parents report that her Kindergarten teacher had concerns about Maria’s learning, but she felt that she was making gradual progress. Now that she is in first grade, her teacher is more concerned. She has told her parents that Maria really seems to struggle with focus, and that it is hard for her to stay on task and complete assignments. She seems to work slowly and “space out” when doing work independently. She also does not appear to listen to directions well, and she seems forgetful at school. She is generally calm and quiet, and she does not have any behavioral issues at school. Her parents have not been previously as concerned as her teachers, as they figured she was just a little bit behind and that she would catch up over time. But her recent standardized testing scores concerned them. They wonder if further evaluation would be helpful.

1) What more information from the history would you like?

*- Did she have any developmental delays early in childhood? Maria was slightly behind in a few early milestones: she learned to walk at 15 months, and she was a bit of a slow talker. But she never received any early intervention services and she largely caught up with her milestones. At the age of 4, she was referred for medically based speech therapy in the community due to a mild language delay/problems with articulation, and she “graduated” from speech therapy before she turned 6. Her parents do not have any concerns about her development otherwise, but she is a bit clumsy and uncoordinated, and her handwriting is quite messy. She does not have an IEP/504 plan.*

*- Birth/past medical history? Normal (full-term, no risk factors, no other medical history)*

*- Family history? Maria is an only child. Her father thinks he had ADHD, and he struggled in school, but he was never treated, and he did not receive any interventions or services. Her mother has two older brothers who had ADHD and were treated with medications, and she has a nephew from one of her brothers who has ADHD and who gets good benefit with Concerta.*

*-Social History: Maria’s mother graduated from high school, and she works part time as an administrator at a local law office. Her father graduated from high school and went to trade school – he now works as a heating/ventilation/air-conditioning technician.*

*-How is she socially? She has multiple friends and she seems to love playing with other kids. She is a bit slow-to-warm-up, but once she knows someone she will happily play with them. She is able to name 3 friends from her school when you ask her.*

*- Sleep: some concerns. She has occasional nightmares and sometimes she has difficulty falling asleep at night. Parents admit that sleep hygiene is not perfect; she usually goes to bed at 8:00pm, but sometimes she will stay up past 9:00pm (even on school nights) if the family is watching something together. She does not have electronic media in her room, and she sleeps in her own room. Sometimes she eats a sugary snack before bed. She has to wake up at 6:30am for school, but on weekends her family lets her sleep in, which can be as late as 8:30am (she sometimes stays up extra late on weekends). She does not snore, and other than the occasional nightmares she does not seem to wake up in the middle of the night. They have never tried melatonin or other sleep aides for her.*

*-Diet: no concerns – she eats a generally healthy diet. She is not constipated.*

*-Any unusual or restrictive behaviors? None. She does not play unusually, fixate, engage in any repetitive movements or mannerisms, or have any impairing sensory differences.*

*-Concerns about anxiety? None. Parents note she is shy with people at first and in novel situations (like a doctor’s appointment), but she generally seems happy and playful. She does seem to shut down a bit at school though, especially when she is put on the spot.*

*-Education: she is 7 and in first grade… if anyone asks about this, she didn’t miss the cutoff for kindergarten, but her parents opted to wait until the next year before starting Kindergarten because they thought she needed some time to mature. She was never held back a grade.*

Your physical examination is normal. You note that she is very quiet, but she will answer your questions appropriately. She makes good eye contact when speaking with you and she seems to be listening when you and her parents are talking. When you ask her how she likes school she says, “I like it,” and when you ask her what her favorite part of school is she says, “Playing outside with my friends.” Neurologic exam is normal.

2) Are there any other physical findings/observations you would like to know about?

*-Neurologic exam is normal, including tone and reflexes.*

*-She does not have any dysmorphic features on her face and body, and her skin and extremity exams are normal.*

*-Growth is normal for age. She is at the 50^th^percentile for weight and the 60^th^ percentile for height.*

*-When you engage in back-and-forth play and conversation with her, she is very appropriate. She smiles and laughs at you when you play a funny face game with her. When you have her draw a picture or herself, it is basic for what you would expect for her age. You ask her to write her name on the paper and it is quite messy with a mix of uppercase and lowercase letters. You ask her to read a few lines from a Dr. Seuss book, and she stops on the first page because she seems embarrassed to read.*

3) What is your differential diagnosis?

*- ADHD (most likely primarily inattentive type)*

*- Delayed development/cognitive impairment (may have borderline cognitive function)*

*- Learning disability*

*- Anxiety*

*- Sleep problems*

*- Typical development with difficult/slow-to-warm-up temperament*

4) What is your plan for today? What can you recommend to her family?

*- Evaluate for ADHD: provide screening tools to her family. Specifically, would provide Vanderbilt screens to parents and teacher(s). Remember, you want to see symptoms and impairment in more than one environment*

*-May consider anxiety evaluation; could provide family with SCARED screens*

*-Sleep hygiene: family has a lot to work on here… encourage residents to discuss what sleep hygiene recommendations they would make. Would they think about melatonin in this patient?*

*-School evaluation: this may be a downstream step, but if they bring it up it is worth discussing. Consider IEP/504 plan evaluation and how it might be helpful for this child (will be discussed in more detail at a follow-up visit)*

*-Follow-up plan: return to clinic after Vanderbilt screens completed*

**Case 3: Uncomplicated ADHD**

**Follow-up Visit #1 (Clinic Visit)**

Recap: Maria is a 7yo girl who presented for inattention and learning difficulties. She has a family history of ADHD in her father, and maternal uncles. She is overall a happy and social child with good peer relationships. She does struggle with sleep initiation. At the last visit, you sent family home with Vanderbilt forms and discussed sleep hygiene.

One month after your initial visit with Maria and her parents, you have a follow-up appointment with them. During this visit, you discuss the Vanderbilt screen results, which showed the following (see handouts):

***Will have printouts of the filled-out forms for the residents; scores are as follows:

Mother:

Questions 1-9: 7/9

Questions 10-18: 3/9

Questions 19-26: 0/8

Questions 27-40: 0/14

Questions 48-55: 1/8

Performance: 4 scores of 4, 0 scores of 5

Father:

Questions 1-9: 5/9

Questions 10-18: 2/9

Questions 19-26: 0/8

Questions 27-40: 0/14

Questions 48-55: 0/8

Performance: 2 scores of 4, 0 scores of 5

Teacher:

Questions 1-9: 8/9

Questions 10-18: 2/9

Questions 19-28: 0/8

Questions 29-35: 0/14

Questions 36-43: 0/8

Performance: 4 scores of 4, 1 score of 5

1) What do these results mean to you? How would you explain them to Maria’s parents?

*The Vanderbilt screens from both home and school show that Maria met criteria for ADHD, primarily inattentive type (cutoff is at least 6/9 criteria), although it is worth noting that her father’s rating scale narrowly did not meet criteria for symptoms (though her mother’s did). How do you reconcile the difference? (Discussion point)*

2) What do you do with this information? Are there other screening tools you can use?

*Encourage residents to discuss if they would consider treating this patient given the scores on the Vanderbilts and the description of her struggles by her parents. Even if the father’s Vanderbilt narrowly misses the symptom cutoff, would you still consider this to be an ADHD diagnosis? Would you want to pursue further evaluation? You could consider other rating scales like the Conners if you wanted to. You could also consider using other behavioral rating scales like the Child Behavior Checklist, which could support your evaluation. Additionally, it is worth considering other causes for her symptoms and impairment like a learning disability, anxiety, etc. In the end, the discussion can be led to the point where we discuss that there are multiple different approaches here, but we will focus on the diagnosis of ADHD and its management: diagnosing ADHD with the information at hand and discussing treatment options is a very reasonable and appropriate choice given the information we have.*

After discussing in detail with her parents, you inform them that you are diagnosing Maria with ADHD, primarily inattentive type. Her parents want to know more about this diagnosis and what it means for Maria going forward, specifically what the treatment options are.

3) Can you describe a treatment approach for Maria’s ADHD?

*There are multiple different treatment approaches one can consider, usually centering on two different principles: medication and behavioral approaches. The combination of medication and behavioral approaches is thought to be superior to any one treatment approach alone. In a situation in which a patient cannot easily access behavioral approaches, treatment with a stimulant alone can be efficacious.*

*If the residents bring this up, it can be a good time to talk about educational supports briefly. This will be covered in more detail at the next follow-up, but a discussion of how accommodations/supports in the classroom can benefit Maria would be appropriate here.*

4) What are the medication options for Maria? What counseling would you provide regarding medication initiation?

*Broadly: stimulants vs. non-stimulants. Evidence supports that stimulants are superior to non-stimulants for treating the symptoms of ADHD. Some non-stimulants (e.g., guanfacine) have been shown to be effective adjuncts to stimulant therapy but are not as beneficial when used as monotherapy. If a parent is opposed to stimulant therapy, non-stimulants like guanfacine and atomoxetine can be discussed.*

*If a parent is agreeable to stimulant therapy, this would be a good place to start. There is nuance to this choice, and many pediatricians may differ on how they approach treatment with stimulants. Discuss with them the different types of stimulants (methylphenidates vs. amphetamines), long-acting vs. short-acting stimulants, delivery methods of stimulants (including what can/cannot be mixed in with something like applesauce or yogurt), and side effects of stimulants.*

*Regarding side effects: encourage residents to come up with these on their own. They should be able to discuss the most common side effects: decreased appetite (with possible plateau/loss of weight), insomnia, headaches, worsening tics (if tics already present; does not cause new tics), and upset stomach. Also cardiovascular side effects like increased HR and BP should be discussed (make sure they know to take a good cardiac history as part of the evaluation for stimulant treatment; for the purposes of this case, her family cardiac history is negative). Note that the HR and BP differences observed with stimulants are usually clinically insignificant but should still be monitored. In general, the risk of sudden cardiac death with stimulants is extremely rare, and the risk is not elevated over children who are not taking stimulants. Still, if any cardiac risk factors exist, an additional evaluation (EKG, cardiology consultation) is warranted prior to starting stimulants (AAP recommendations).*

*Also discuss strategies to mitigate certain side effects (such as for appetite suppression: encourage giving medication after breakfast, giving some preferred foods for lunch, especially at school, and providing healthy snacks after school when the medication is likely wearing off).*

*Side effects of non-stimulants:*

*-Atomoxetine: increased HR and BP, somnolence, GI symptoms. Also has black box warning for increased suicidal thinking in pediatric patients*

*-Guanfacine: decreased BP and HR, somnolence, dry mouth, headache, abdominal pain.*

5) Are there other non-medicinal options you would recommend for Maria?

*Behavioral therapy: Can be very helpful, especially when combined with medication. Also discussing behavior management strategies with her parents can be helpful. Sometimes even check-ins with a school counselor can be helpful. Ideally a child can work on learning strategies to help with their lack of focus (i.e. how can they refocus themselves) and on how to control their impulses and bodies (for the more hyperactive types).*

*This can also be a place to discuss dietary/supplemental considerations: In general, dietary changes (such as gluten-free diets or removing food dyes or additives) have limited and conflicting research on how well they work for ADHD symptoms. Polyunsaturated fatty acids (PUFAs), particularly PUFAs with a high omega-3 level (or a high ratio of omega-3 to omega-6) have some evidence supporting that they are beneficial in decreasing ADHD symptoms in children and adolescents and possibly improving cognitive function. However, this is not strong evidence, and there are also studies that show they are no better than placebo. There are some studies that have shown that certain subgroups of children with ADHD tend to have more benefit from PUFA supplementation (particularly those with other co-morbidities or developmental disorders). There are no significant side effects of these supplements, and the most frequently reported ones include mild dyspepsia and incidental nosebleeds. Many parents may ask about these supplements for their children, and it is helpful to be able to discuss this with them. In general, they are safe, but it is difficult to know if they will be efficacious or not.*

*Pharmacogenetic tools (e.g. GeneSight Testing): lack of evidence to support such testing to determine which stimulants/non-stimulants may be best for a given child. Such tools are not recommended by the AAP.*

1. What is your plan for Maria? If you start a medication which one would you choose?

*Let the residents discuss and decide what medication they would use. In the next visit, we will default to using long-acting methylphenidate (Concerta) for this case, but they can discuss what they would use and why. Also focus on follow-up plans: how long would they take before they checked in with her parents about the efficacy/side effects of the medication they choose? What is their plan for titration? A lot of flexibility here, but in general they should plan on having a phone follow-up within the first few weeks of starting the medication and then should bring the patient in in-person within the first few months to check vitals and weight and to perform a physical exam. Titration of doses can take place usually over a 7-day basis, but it can be even shorter if indicated.*

**Case 3: Uncomplicated ADHD**

**Follow-up Visit #2 (Virtual/Phone Visit)**

Recap: Maria is a 7yo girl you have diagnosed with ADHD and started on long-acting methylphenidate (Concerta) 18mg daily at her last visit.

Today is a one-month virtual follow-up since you last saw her. In the interim, Maria’s mother called the clinic because while the 18mg of long-acting methylphenidate (Concerta) was working somewhat, she was still having issues with focus and inattention at school, especially in the afternoon. Since she was not having significant side effects (only mild appetite suppression), one of your colleagues increased her from 18mg to 27mg daily of long-acting methylphenidate (Concerta) based on your previous titration plan (you were on leave when Maria’s mother called). Maria’s mother reports that Maria has been more focused and attentive at school since she has been on the 27mg of long-acting methylphenidate (Concerta).

1) What questions do you have for Maria’s mother regarding her stimulant therapy?

*- Efficacy: home and school*

*- Side effects*

*- Other Concerns?*

*When you ask her about how she is doing, she tells you the following: “Maria is more attentive and focused through much of the day at school, but she still has challenges staying on task and completing assignments. She is still struggling with reading, too. At home, she is kind and pleasant, but when the medication wears off in the afternoon she is much less focused and a little emotional. It is hard to get her to complete chores or homework after school.”*

*When you ask her about side effects, she tells you: “Maria’s appetite has continued to go down, and it is worse on the 27mg dose. She has never been much of an eater at breakfast, but we make sure she eats something before taking the long-acting methylphenidate (Concerta). Her teachers say she doesn’t seem to eat much at lunch or snack when she is at school, and she often comes home with food in her lunch box. At around 4:00pm, she seems hungry and wants a small snack, and then she usually eats a good dinner. But we are worried about how low her appetite is – she usually would eat her lunch and snack at school. She is sleeping well overall. But there are some nights when it takes her a little longer than usual to fall asleep. It’s not a big change, but it is different. She is not having any of the other side effects that you told me about.”*

2) How do you respond to what Maria’s mother told you about the efficacy of the medication and the side effects that Maria is experiencing?

*It seems that the long-acting methylphenidate (Concerta) is offering some benefit at this dose, but it also has caused multiple side effects. It seems that they are working on managing the side effects, but it may be hard to justify titrating the dose upwards given the side effects.*

3) What is your approach to managing her stimulant regimen?

*There are multiple different approaches to this problem.*

*-You could titrate the long-acting methylphenidate (Concerta) up and assess/manage the side effects better: consider strict sleep hygiene and possibly melatonin for sleep, focus strongly on increasing calories during meals to combat the appetite suppression (or even consider an appetite stimulant like Periactin), and behavioral/educational approaches for the challenges experienced at school and at home.*

*-Another option would be to consider adjunctive therapy. A non-stimulant like Intuniv would be a reasonable option here if you are ok with the efficacy from the stimulant but would like better symptom control and want to avoid the negative side effects of stimulants. In other words, you can improve symptom control without worrying about worse appetite, insomnia, etc. This is not always effective, and it can take time to titrate to effect with a non-stimulant, but it is an option. Remember to counsel parents about the side effects of non-stimulants if you do decide to go this route.*

*-Still, it may be that long-acting methylphenidate (Concerta) is not the right fit for her. While many studies have shown that stimulant trials are often stopped prematurely (i.e. before titrating to an appropriate dose), in this case the long-acting methylphenidate (Concerta) seems to be only modestly helpful with some difficult-to-manage side effects. Considering changing her stimulant is a very reasonable option here. You could try another stimulant within the same class [i.e. another methylphenidate such as other formulations of methylphenidate (e.g., Ritalin) or dexmethylphenidate], or you could switch classes to an amphetamine. Studies have shown that 60-70% of children and adolescents with ADHD will respond well to a methylphenidate, and about 90% will receive benefit from a stimulant of either class. So, regardless of what you switch to you have a good chance of getting good symptom control.*

Maria’s mother agrees with you that even with the side effects experienced with long-acting methylphenidate (Concerta), it has been helpful for Maria. She was pleased with the benefit she experienced and hopeful that another stimulant might be a better fit for her. In spite of this, she remains concerned about Maria’s learning: “Even if we manage her ADHD symptoms, I am worried that she won’t be able to do well in school, especially with reading. Are there any other supports or interventions that could help Maria in school?”

4) What is your response to Maria’s mother? Are there other supports, educational or otherwise, that you may recommend to Maria’s mother?

*We want them to be thinking about educational supports, so as a follow up question:* *How would you counsel Maria’s mother about having Maria evaluated for special education? What special education services might be beneficial for Maria going forward?*

*Maria likely would benefit from school supports, especially given her learning challenges. Children and adolescents with ADHD may be eligible for services as part of a 504 plan or an IEP (designation of “other health impairment”). Note that not all ADHD qualifies as a disability under an IEP – it needs to severely impair a child’s ability to learn. But, even if a child does not qualify for an IEP, they may qualify for a 504 plan. Regardless of the plan put into place, a child may receive both interventions and accommodations to help them in the learning environment. For Maria, an IEP evaluation may be helpful in that it may also be able to help clarify if anything else, such as a specific learning disability, could explain her problems learning in school.*

*It is important to counsel families on how to approach the school system for an evaluation (provide handout here). Families seeking an evaluation for their child should contact their child’s school or school district and speak to the special education administrator or IEP coordinator, letting them know that they would like their child evaluated for special education. They should ask about the process and get more information. Then, they should put* ***in writing*** *that they would like their child assessed for an IEP or a 504 plan, and they should explain their concerns. Once they request the evaluation in writing, the school has 30 days to create a plan to evaluate and to obtain consent from the parents. Note that the parents have the right to consent to the school’s plan or not. Once consent is obtained, within the next 60 days the evaluation (including all assessments of cognition, function, etc.) takes place. Also within this time frame, a meeting to discuss the evaluation and plan is set with the family. If it is determined that a child qualifies for an IEP, then they write the IEP and the family has an opportunity to review and consent. Note that just because a particular child may not qualify for an IEP, it does not mean that they do not or should not qualify as having a disability that may impact their learning. In this case, they can still qualify for a 504 plan, which can provide accommodations. For a child with ADHD, this may include things such as preferential seating away from distractions, visual or auditory aids for understanding directions, providing frequent movement breaks, and providing extra assistance in organization of time and materials.*

5) What is your follow-up plan for Maria? How will you manage her ADHD going forward?

*Make sure the learners commit to a plan (any of the 3 options above from question 3 are reasonable). At some point in the next 2-4 months, you will likely want to see Maria in-person to check her weight and her vitals, especially given the concerns about her appetite. It will also be beneficial in the next 3-4 months to follow-up on how the family is doing with the special education process. If you made medication changes, it may be a good idea to have a virtual follow up in the next 1-2 weeks to discuss how she is doing.*

**Additional Resources:**

Attention-Deficit/Hyperactivity Disorder (ADHD): Parent Training. Available at: <https://www.cdc.gov/ncbddd/adhd/behavior-therapy.html>. Accessed 03 Sep 22.

Center for Parent Information and Resources. Available at: [www.parentcenterhub.org](http://www.parentcenterhub.org/) Accessed 03 Sep 22.

Children and Adults with Attention-Deficit/Hyperactivity Disorder (CHADD) National Resource Center for ADHD: For Professionals. Available at: <https://chadd.org/for-professionals/overview/> Accessed 03 Sep 22.

Lipkin, PH, Okamato J. The individuals with disabilities education act (IDEA) for children with special educational needs. Council on Children with Disabilities and Council on School Health. Pediatrics. 2015;136(6):e1650-e1662.

National Center for Learning Disabilities. Available at: <https://www.ncld.org/research/> Accessed 03 Sep 22.

Wolrach ML, Hagan JF, Allan C, et al; Subcommittee on children and adolescents with attention-deficit/hyperactivity disorder. Clinical practice guideline for the diagnosis, evaluation, and treatment of attention-deficit/hyperactivity disorder in children and adolescents. Pediatrics. 2019;144(4):e20192528.
