# Supplementary material for: A Case-Based, Longitudinal Curriculum in Pediatric Behavioral and Mental Health
Source: MedEdPORTAL. 2024 Apr 29;20:11400. doi: 10.15766/mep_2374-8265.11400 (PMC11056487; doi:10.15766/mep_2374-8265.11400)
Supplement: Supplementary file 1 — Preteen Anxiety Case - Residents.docxPreteen Anxiety Case - Faculty Guide.docxPreteen Anxiety Case - SCARED Forms.pdfAnxiety Resources Handout.docxASD Delays Case - Residents.docxASD Delays Case - Faculty Guide.docxAutism Summary Handout and Resources.docxDepression Case - Residents.docxDepression Case - Faculty Guide.docxDepression Resources Handout.docxSchool-age ADHD Case - Residents.docxSchool-age ADHD Case - Faculty Guide.docxSchool-age ADHD Case - Vanderbilts.pdfADHD Handout.docxYoung ADHD and Behavior Case - Residents.docxYoung ADHD and Behavior Case - Faculty Guide.docxParenting Handout and Resource Sheet.docxBehavioral and Mental Health Curriculum Survey.docxBehavioral and Mental Health Pre-Post Test.docx [file mep_2374-8265.11400-s001.zip › N. ADHD Handout.docx]

**ADHD Summary Sheet and Resources Guide**

***DSM-5 Criteria for Attention Deficit Hyperactivity Disorder***

Symptoms and/or behaviors that have persisted greater than or equal to 6 months in at least 2 settings (e.g. school, home, church). Symptoms have negatively impacted academic, social, and/or occupational functioning. In patients age less than 17 years, at least 6 symptoms are necessary; in those age 17 years or older, at least 5 symptoms are necessary.

1. Inattentive type diagnostic criteria
   1. Displays poor listening skills
   2. Loses and/or misplaces items needed to complete activities or tasks
   3. Sidetracked by external or unimportant stimuli
   4. Forgets daily activities
   5. Diminished attention span
   6. Lacks ability to complete schoolwork and other assignments to follow instructions
   7. Avoids or is disinclined to begin homework or activities requiring concertation
   8. Fails to focus on details and/or makes thoughtless mistakes in schoolwork or assignments
2. Hyperactive/Impulsive type diagnostic criteria
   1. Squirms in seated or fidgets with feet/hands
   2. Marked restlessness that is difficult to control
   3. Appears to be driven by “a motor” or is often “on the go”
   4. Lacks ability to play and engage in leisure activities in a quiet manner
   5. Incapable of staying seated in class
   6. Overly talkative
   7. Difficulty waiting turn
   8. Interrupts or intrudes into conversations and activities of others
   9. Impulsively blurts out answers before questions completed
3. Additional requirements for diagnosis
   1. Symptoms present prior to age 12 years
   2. Symptoms not better accounted for by a different psychiatric disorder (e.g. mood disorder, anxiety disorder) and do not exclusively occur during a psychiatric disorder (e.g. schizophrenia)
   3. Symptoms not exclusively a manifestation of oppositional behavior
4. Classification
   1. Combined type: patient meets both inattentive and hyperactive/impulsive criteria for the past 6 months
   2. Predominantly inattentive type: patient meets inattentive criterion, but not hyperactive/impulse criterion, for the past 6 months
   3. Predominantly hyperactive/Impulsive type: patient meets hyperactive/impulse criterion, but not inattentive criterion, for the past 6 months
5. Diagnostic Tools: The NICHQ Vanderbilt Assessment Scale and the Conners Rating Scale are commonly used clinical assessments for ADHD

***Treatment approach for ADHD***

1. Behavioral approaches are often considered first-line, especially for mild symptoms. That said, accessing behavioral health supports for ADHD may be difficult, depending on the situation/location.
2. Medication is often considered in most moderate-to-severe cases, either alone or in combination with behavioral approaches.
3. Medication Treatment Algorithm
   1. Stimulants vs. Nonstimulants
      1. Stimulants are considered first-line therapy for ADHD
      2. In some cases, nonstimulants are considered as first-line (family preference against stimulants, concern about certain side effects caused by stimulants, etc.)
   2. Stimulants: decision tree: methylphenidate vs. amphetamine stimulants; short-acting vs. long-acting
      1. Methylphenidate stimulants
         1. Brand names: Ritalin, Metadate, Focalin (dexmethylphenidate), Concerta, Quillivant, Daytrana, and others
      2. Amphetamine stimulants
         1. Brand names: Adderall, Vyvanse, Dexedrine, and others
      3. Short-acting vs. Long-acting
         1. Short-acting stimulants have a duration of action of 3-4 hours
         2. Long-acting stimulants have a much longer duration of action, typically 8-12 hours depending on the medication
      4. Treatment strategy: typically, providers choose a starting class of stimulant based on clinical experience
         1. Up to 70% of individuals with ADHD will respond to a methylphenidate, and up to 90% will respond to either a methylphenidate or amphetamine
         2. Many providers choose to start with long-acting medication, though in some cases (especially with much younger children), short-acting stimulants may be used first
         3. Typically, the approach is to start with the lowest dose and titrate to effect
      5. Specific stimulant notes
         1. Long acting methylphenidate (specifically Concerta): a long-acting methylphenidate; uses osmotic release system with three release points during the day; tends to last long (up to 12 hours)
         2. Lisdexamfetamine (Vyvanse): a long-acting amphetamine; a prodrug that is metabolized into an active form; lasts up to 12-14 hours
         3. Dexmethylphenidate (Focalin): the dex-enantiomer of methylphenidate; the extended release form can last up to 8-10 hours; also has a short-acting form
         4. Ritalin brand name: a common methylphenidate with both short and long-acting forms; the long-acting form can last up to 8 hours
         5. Adderall: a common amphetamine with both short and long-acting forms; the long-acting form can last up to 10 hours
         6. Quillivant: an extended release liquid methylphenidate; may last up to 8 hours
         7. Daytrana: a methylphenidate patch; can last up to 10 hours
      6. Other treatment considerations
         1. Route of administration: Concerta is a tablet that must be swallowed whole; most other extended release stimulants are capsules that can be sprinkled into yogurt or applesauce (Vyvanse can also be sprinkled into orange juice or water)
         2. Pharmacokinetics: it usually takes about 60 minutes for stimulants to peak in the bloodstream, but sometimes it can take up to 2 hours; because stimulants are out of the body by the end of the day, they do not need to build up to steady state to be effective and thus do not need to be tapered when discontinued
      7. Side effects:
         1. Common stimulant side effects include: appetite suppression, insomnia, headaches, tics (not caused by stimulants, but can be exacerbated by them), stomachaches
         2. Young children can also have emotional lability, especially when the stimulant wears off
         3. Stimulants can also rarely cause palpitations; in children with a personal or family history of significant cardiac disease (notably, arrhythmias), consultation and evaluation by a cardiologist is recommended
   3. Nonstimulants: not often used first-line; can be used as adjunctive therapy with stimulants in some cases
      1. Guanfacine: Intuniv (Extended Release Guanfacine): a common adjunct with stimulants; rarely used as monotherapy; is a tablet that must be swallowed whole; has age and weight-based dosing; lasts up to 24 hours and typically will build up to steady state; needs to be tapered off if discontinuing
         1. Side effects: sedation, headaches, constipation; generally well-tolerated
      2. Atomoxetine (Strattera): rarely used as monotherapy for ADHD. Comes in a capsule that can be sprinkled; has age and weight-based dosing; last 10-12 hours
         1. Side effects: black-box warning for suicidal thinking; weight loss, abdominal pain, decreased appetite, headaches are most common. May also cause cardiac problems, so should consult with specialist before starting if there is a personal or family cardiac history

***School-Based Supports/Recommendations for ADHD***

Both IEPs and 504 plans fall under federal laws, but each state and local school has its own interpretation.

| IEP | 504 |
| --- | --- |
| - Funding provided through Individuals with Disabilities Education Act (IDEA) - Ages 3-21 - Federal law that focuses on ensuring that the school meets the needs of children with specific kinds of disabilities so that they make adequate academic progress based on what they know and can learn - Measurable annual goals and objectives, statements regarding current performance and disability, plan for informing parents, plan for achievement testing, need for extended school year, transition requirements - Have identified disability that impedes learning to point needs specialized instruction to ensure meaningful educational progress - 14 defined diagnoses (autism, deaf-blindness, deafness, developmental delay, emotional disturbance, hearing impairment, intellectual disability, multiple disabilities, orthopedic impairment, other health impairment, specific learning disability, speech or language impairment, traumatic brain injury, visual impairment including blindness) | - Part of rehabilitation act of 1973 - Protects people with disabilities by eliminating barriers and allowing full participation - Ensuring that the school provides children with disabilities or impairments that services and accommodations necessary in order to access everything that other children can access at school, including curriculum - School can provide accommodations without a formal evaluation |

Author Owned

Timeline:

One request made in writing and consent obtained school has 60 days to complete evaluation.

Once evaluation complete school has 30 days to create IEP.

*References:*

1. American Psychiatric Association. (2013). Diagnostic and statistical manual of mental disorders (5th ed.). <https://doi.org/10.1176/appi.books.9780890425596>
2. Wolraich ML, Hagan JF, Allan C, et al. Clinical Practice Guideline for the Diagnosis, Evaluation, and Treatment of Attention-Deficit/Hyperactivity Disorder in Children and Adolescents. Pediatrics (2019) 144 (4): e20192528. <https://doi.org/10.1542/peds.2019-2528>
3. Morin, A. (2014). *The Everything Parent’s Guide to Special Education*. Simon & Schuster, Inc.
