# Supplementary material for: A Case-Based, Longitudinal Curriculum in Pediatric Behavioral and Mental Health
Source: MedEdPORTAL. 2024 Apr 29;20:11400. doi: 10.15766/mep_2374-8265.11400 (PMC11056487; doi:10.15766/mep_2374-8265.11400)
Supplement: Supplementary file 1 — Preteen Anxiety Case - Residents.docxPreteen Anxiety Case - Faculty Guide.docxPreteen Anxiety Case - SCARED Forms.pdfAnxiety Resources Handout.docxASD Delays Case - Residents.docxASD Delays Case - Faculty Guide.docxAutism Summary Handout and Resources.docxDepression Case - Residents.docxDepression Case - Faculty Guide.docxDepression Resources Handout.docxSchool-age ADHD Case - Residents.docxSchool-age ADHD Case - Faculty Guide.docxSchool-age ADHD Case - Vanderbilts.pdfADHD Handout.docxYoung ADHD and Behavior Case - Residents.docxYoung ADHD and Behavior Case - Faculty Guide.docxParenting Handout and Resource Sheet.docxBehavioral and Mental Health Curriculum Survey.docxBehavioral and Mental Health Pre-Post Test.docx [file mep_2374-8265.11400-s001.zip › O. Young ADHD and Behavior Case - Residents.docx]

**Case 5**

**Initial Visit**

CC: behavior and school concerns

Miles is 4-year-old African American boy who presents with his mother due to behavioral concerns. He was just kicked out of his second preschool, and mom is very concerned because her husband is deployed and she works full time, so she needs him to be in preschool or some other childcare during the day. They just moved to Ohio from Florida, where he was also recently kicked out of a daycare for disruptive behavior. She thinks Miles has always been an “on-the-go” and strong-willed child, but his behaviors seem to be escalating recently. She is looking for advice on what to do next and how to handle his behaviors.

1) What more information from the history would you like?

Your physical examination is normal, aside from noting that he frequently runs around the room and is opening and closing cabinets as mom is telling him to stop and sit down. Neurologic exam is normal.

2) What is your differential diagnosis?

3) What is your plan for today? What can you recommend to family?

4) What other medical concerns do you want to rule out or other testing would you like to order?

**Case 5: Younger Child Behavioral Concerns/ADHD Case**

**Follow-up Visit #1 (Virtual/Phone Visit)**

Recap: Miles is a 4yo boy who presented one month ago for behavioral concerns after being kicked out of preschool. You learned of some social stressors including frequent moves and parental deployment. You discussed some behavioral techniques for family to try at home following the A-B-C model and referred them to counseling for ongoing support.

One month after your initial visit with Miles and his mother, you have a follow-up virtual/phone appointment with her. On the phone, she tells you that she was able to establish care with a child psychologist, who is concerned for ADHD. The mother is worried about making this diagnosis in such a young child and wants to know what is different in how we diagnose and treat ADHD in younger children. Do you agree a diagnosis can be made this early? She wants to know whether you think we should do medication or some other treatment.

1) How do you respond?

2) If you were conducting this visit in a remote location (e.g., rural Kentucky) with little or no access to behavioral therapy, what would your next step be?

**Case 5: Younger Child Behavioral Concerns/ADHD Case**

**Follow-up Visit #2 (Clinic Visit)**

Recap: Miles is a 4yo boy recently diagnosed with ADHD. At your last visit, you discussed parent treatment in behavioral management as first-line treatment, which the family was happy to pursue.

Two months later, Miles and his mother return again. They have been working on behavioral therapy and have noticed some improvements, but his mother notes that he is still struggling with hyperactivity both at school and home. While he is having less aggression since starting the counseling, he is still running around his class, having trouble staying on task when they do circle time, and even sitting down to listen to a book or stay in his seat at the dinner table at home. Mom wants to know what to do next.

1) How do you respond?

2) Mom states she is open to medication, but she is worried about starting medication in a child this young. What is different for starting medication in children this age? Are there long-term side effects of stimulants in young children? Decide which medication you would recommend.

3) A final concern brought up today is that he is having trouble going to bed at night. She is worried that the stimulants will make this worse. How do you counsel the family on this concern?
