# Supplementary material for: A Case-Based, Longitudinal Curriculum in Pediatric Behavioral and Mental Health
Source: MedEdPORTAL. 2024 Apr 29;20:11400. doi: 10.15766/mep_2374-8265.11400 (PMC11056487; doi:10.15766/mep_2374-8265.11400)
Supplement: Supplementary file 1 — Preteen Anxiety Case - Residents.docxPreteen Anxiety Case - Faculty Guide.docxPreteen Anxiety Case - SCARED Forms.pdfAnxiety Resources Handout.docxASD Delays Case - Residents.docxASD Delays Case - Faculty Guide.docxAutism Summary Handout and Resources.docxDepression Case - Residents.docxDepression Case - Faculty Guide.docxDepression Resources Handout.docxSchool-age ADHD Case - Residents.docxSchool-age ADHD Case - Faculty Guide.docxSchool-age ADHD Case - Vanderbilts.pdfADHD Handout.docxYoung ADHD and Behavior Case - Residents.docxYoung ADHD and Behavior Case - Faculty Guide.docxParenting Handout and Resource Sheet.docxBehavioral and Mental Health Curriculum Survey.docxBehavioral and Mental Health Pre-Post Test.docx [file mep_2374-8265.11400-s001.zip › P. Young ADHD and Behavior Case - Faculty Guide.docx]

**Case 5: Younger Child Behavioral Concerns/ADHD Case**

**Learning Objectives**

By the end of the initial and follow-up cases, learners will be able to:

1. Build a comprehensive differential for behavioral difficulties in young children of at least four diagnoses
2. Recommend counseling and in-office behavioral techniques to families
3. Explain differences in ADHD diagnosis and management in young children (4-5 years old) as compared to older children
4. Discuss healthy sleep strategies for school-age children

**Initial Visit**

CC: behavior and school concerns

Miles is 4-year-old African American boy who presents with his mother due to behavioral concerns. He was just kicked out of his second preschool, and mom is very concerned because her husband is deployed and she works full time, so she needs him to be in preschool or some other childcare during the day. They just moved to Ohio from Florida, where he was also recently kicked out of a daycare for disruptive behavior. She thinks Miles has always been an “on-the-go” and strong-willed child, but his behaviors seem to be escalating recently. She is looking for advice on what to do next and how to handle his behaviors.

1) What more information from the history would you like?

- *Past medical history: allergic rhinitis; born at 35+6 weeks EGA due to PPROM, otherwise uncomplicated pregnancy; passed newborn and hearing screens at birth*
- *Medications: Zyrtec PRN for allergies*
- *Past surgical history: none*
- *Family history: ADHD in father and uncle*
- *Social: Parents have been married for 7 years. His father works in logistics as an Active Duty Air Force officer, and his mother is an administrative assistant. They have moved moved twice within the past four years. FOC travels about four times per year, which is stressful for MOC. If asked, can mention that there has been marital stress recently – many arguments focus on how to discipline Miles and has led to yelling. MOC feels safe, no physical violence between parents. They have never been to counseling.*
- *Safety concerns – running into the road, other escape behaviors? No.*
- *A/B/C model questions*
  - *Antecedents: what happens before the behaviors? What seems to trigger them?*
  - *Behavior: what are the actual behaviors?*
  - *Consequences: What happens after/consequences both at school and home? How are they handled by his caregivers?*
- *Answers to above:*
  - *A: Being told “no,” transitions between activities, needing to sit in circle time, having something taken from him*
  - *B: Yelling, running around, grabbing other children’s toys, not sitting still, hitting other children when upset/others take his toys. At home, he is aggressive toward his siblings (6yo sister and 2yo brother). Throws tantrums.*
  - *C: school – mom not sure, thinks he goes to time out; home – tell him no, does some time outs, sometimes yells but tries not to, screen time will calm him down; they have also tried spanking, unsure if helpful. MOC is stricter than FOC with discipline, which frustrates her. He sometimes tries to hit back when spanked, although they think it does stop the behavior immediately.*
- *Developmental history*
  - *Concern he was behind in speech (not many words at 18 months) and he received some services from early intervention in FL, but he seemed to catch up to his peers so did not continue services after age 3. No private ST, OT, or PT.*
  - *18mo ASQ was gray zone for communication and personal/social; passed 18mo. He passed audiology eval at 18mo.*
  - *24mo ASQ was gray for communication, problem solving, and personal/social; passed MCHAT again.*
- *Ongoing developmental concerns? No motor concerns, still thinks he speech is hard for some people to understand but vocabulary is much improved.*
  - *Autism-type symptoms? Restrictive interests and/or repetitive behaviors? Social interactions with other children – does he have interest in children his age? Joint attention? Answer: no specific concerns, no repetitive behaviors or restrictive interests, has interest in children his age, does display joint attention (note – ensure learners know what joint attention means)*
  - *Anxiety screening questions – does he express fear about school or other activities? Separation anxiety from father? Did these behaviors worsen around the time of deployment and/or move? Answer: MOC does note some general irritability, which did worsen around dad’s deployment and their move. He does not specifically state he is worried about school and does not resist going to school/drop-offs.*

Your physical examination is normal, aside from noting that he frequently runs around the room and is opening and closing cabinets as mom is telling him to stop and sit down. Neurologic exam is normal.

2) What is your differential diagnosis?

- *ADHD*
- *ODD*
- *Autism*
- *Adjustment disorder/family stressors*
- *Anxiety*
- *Parent-child interaction concerns*
- *Systemic racism – was he more likely to be kicked out b/c he is African American? While certainly not the only issue at hand, it is worth acknowledging this could be a factor in how he has been treated at school.*

3) What is your plan for today? What can you recommend to family?

- *Referral to counseling – family is on board but wants to know what they can do in the meanwhile*
- *Paying attention to A/B/Cs*
  - *Is there a way to avoid some triggers? Visual chart to organize the day, anticipate transitions*
  - *How to address behaviors? Incentive charts for good behaviors, being specific about expectations. When making verbal corrections, get down on eye level with them, tell them what you want him to do (instead of just telling him “no” or “stop” or what you don’t want him doing). Praising and reinforcing good behavior – catch him doing something good. Avoiding spanking or other physical discipline, particularly for children who are displaying aggression toward other children – not a long-term effective solution, can model undesirable behavior.*

4) What other medical concerns do you want to rule out or other testing would you like to order?

- *Hearing and vision assessments*
- *Speech evaluation*
- *Lead testing/risk factors assessment*
- *Double check no other medications (e.g., Benadryl at night to help with sleep)*

**Case 5: Younger Child Behavioral Concerns/ADHD Case**

**Follow-up Visit #1 (Virtual/Phone Visit)**

Recap: Miles is a 4yo boy who presented one month ago for behavioral concerns after being kicked out of preschool. You learned of some social stressors including frequent moves and parental deployment. You discussed some behavioral techniques for family to try at home following the A-B-C model and referred them to counseling for ongoing support.

One month after your initial visit with Miles and his mother, you have a follow-up virtual/phone appointment with her. On the phone, she tells you that she was able to establish care with a child psychologist, who is concerned for ADHD. The mother is worried about making this diagnosis in such a young child and wants to know what is different in how we diagnose and treat ADHD in younger children. Do you agree a diagnosis can be made this early? She wants to know whether you think we should do medication or some other treatment.

1) How do you respond?

- *ADHD can be diagnosed in younger children 4-5 years old. The most recent AAP guidelines are clear that the same DSM-5 criteria to diagnose older children can be used in preschool-age children also.*
- *Any DSM-based rating scale can be used in children this age and should be obtained from caregivers in multiple settings (just like in older children).*
- *Parent training in behavior management (PTBM) is the first-line treatment. Although we do not advocate for any particular program, examples of PTBM programs include parent-child interaction therapy (PCIT), Triple P Parenting courses, and the Incredible Years.*

2) If you were conducting this visit in a remote location (e.g., rural Kentucky) with little or no access to behavioral therapy, what would your next step be?

- *Reinforce behavioral management skills to be used at home*
- *If PTBM is not available, or if it is tried and ineffective, it is reasonable to prescribe stimulant medication for moderate-to-severe cases of ADHD*
- *If medication is used, methylphenidate has better evidence for safety and efficacy in younger children, even though amphetamine has FDA approval for use in children <6yo (the approval happened in an era with less stringent criteria, but data since that time support the use of methylphenidate).*

**Case 5: Younger Child Behavioral Concerns/ADHD Case**

**Follow-up Visit #2 (Clinic Visit)**

Recap: Miles is a 4yo boy recently diagnosed with ADHD. At your last visit, you discussed parent treatment in behavioral management as first-line treatment, which the family was happy to pursue.

Two months later, Miles and his mother return again. They have been working on behavioral therapy and have noticed some improvements, but his mother notes that he is still struggling with hyperactivity both at school and home. While he is having less aggression since starting the counseling, he is still running around his class, having trouble staying on task when they do circle time, and even sitting down to listen to a book or stay in his seat at the dinner table at home. Mom wants to know what to do next.

1) How do you respond?

- *Double check what parenting changes have been made and how they are working*
- *Gauge how impairing the persistent hyperactive behaviors are. Answer: mom reports they are quite impairing, not allowing him to participate fully in class activities, causing significant stress at home*
- *Offer a trial of medication.*

2) Mom states she is open to medication, but she is worried about starting medication in a child this young. What is different for starting medication in children this age? Are there long-term side effects of stimulants in young children? Decide which medication you would recommend.

- *Reinforce from last visit: methylphenidate has better evidence for safety and efficacy in younger children, even though amphetamine has FDA approval for use in children <6yo (the approval happened in an era with less stringent criteria, but data since that time support the use of methylphenidate). Refer to ADHD medication reference chart to discuss options. Also consider which medications (such as Adderall) are available as liquid formulations or sprinkle tabs for children who cannot swallow pills.*
- *Start at lowest dose since probably metabolized slower, increase only in small increments*
- *Might see a higher risk of mood changes*
- *Little data exists about long-term effects on brain development and growth, but generally well tolerated*

3) A final concern brought up today is that he is having trouble going to bed at night. She is worried that the stimulants will make this worse. How do you counsel the family on this concern?

- *Assess sleep hygiene*
  - *Bedtime routine – consistency, timing, activities*
  - *Limiting screen time before bedtime for at least 1 hour*
  - *Physical activity during the daytime*
  - *Any possible sleep associations? A sleep association is something the child needs to go to sleep (e.g., a blanket or stuffed animal), but if it is not present when they wake up overnight, they will have problems going back to sleep. A blanket is not problematic in this regard, but if a parent is laying down with them to fall asleep, for example, it will be hard for them to fall asleep alone or to fall back asleep if they awaken overnight.*
- *Additional information needed:*
  - *Is it a problem with sleep initiation? Sleep maintenance?*
  - *When do they put him down? How long does it take him to fall asleep?*
  - *If he gets up out of bed, how do they handle it?*
  - *Any sleep training methods they have already tried? How did they work?*
- *Possible recommendations*
  - *Sleep hygiene*
  - *Melatonin – 1mg starting dose, OK to go up to about 3mg in his age (5-6mg max in older children)*
  - *Sleep training techniques – graduated extinction (providing less interaction each time they go in to check on the child or space out the times in which they check on them), fading (pushing bedtime back to when they are usually sleepy to take advantage of tiredness, then moving it back up slowly over time), or bedtime pass method (give child a token they can use once per night to get up to have an extra hug, extra water, etc, but after then they cannot get up; if they don’t use their token they can redeem it for a small reward or a sticker on a sticker chart) might be a good fit for his age range.*

**Additional Resources:**

American Academy of Pediatrics. AAP offers parent tips and resources for dealing with COVID-19 and its stresses. Available at: <https://www.aap.org/en/news-room/news-releases/aap/2020/aap-offers-parent-tips-and-resources-for-dealing-with-covid-19-and-its-stresses/> Accessed 03 Sep 22.

Attention-Deficit/Hyperactivity Disorder (ADHD): Parent Training. Available at: <https://www.cdc.gov/ncbddd/adhd/behavior-therapy.html>. Accessed 03 Sep 22.

Bauer NS, Childers DO, Curtin M. Principles of positive parenting can be shared during pediatric visits. AAP News. 2016. Available at: <https://publications.aap.org/aapnews/news/13135> Accessed 03 Sep 22.

Centers for Disease Control and Prevention. Watch Me! Celebrating milestones and sharing concerns. Available at: <https://www.cdc.gov/ncbddd/watchmetraining/index.html> Accessed 03 Sep 22.

Children and Adults with Attention-Deficit/Hyperactivity Disorder (CHADD) National Resource Center for ADHD: For Professionals. Available at: <https://chadd.org/for-professionals/overview/> Accessed 03 Sep 22.

Gleason MM, Egger HL, Emslie GJ, et al. J Psychopharmacological treatment for very young children: contexts and guidelines. Child Adoles Psych. 2007;46(12):1532-72.

Kisner K, MacLaughlin SS, Parlakian R. Nine elements that power positive parenting. Zero to Three. Available at: <https://www.zerotothree.org/resource/nine-elements-that-power-positive-parenting/> Accessed 03 Sep 22.

Wolrach ML, Hagan JF, Allan C, et al; Subcommittee on children and adolescents with attention-deficit/hyperactivity disorder. Clinical practice guideline for the diagnosis, evaluation, and treatment of attention-deficit/hyperactivity disorder in children and adolescents. Pediatrics. 2019;144(4):e20192528.
