# Supplementary material for: A Case-Based, Longitudinal Curriculum in Pediatric Behavioral and Mental Health
Source: MedEdPORTAL. 2024 Apr 29;20:11400. doi: 10.15766/mep_2374-8265.11400 (PMC11056487; doi:10.15766/mep_2374-8265.11400)
Supplement: Supplementary file 1 — Preteen Anxiety Case - Residents.docxPreteen Anxiety Case - Faculty Guide.docxPreteen Anxiety Case - SCARED Forms.pdfAnxiety Resources Handout.docxASD Delays Case - Residents.docxASD Delays Case - Faculty Guide.docxAutism Summary Handout and Resources.docxDepression Case - Residents.docxDepression Case - Faculty Guide.docxDepression Resources Handout.docxSchool-age ADHD Case - Residents.docxSchool-age ADHD Case - Faculty Guide.docxSchool-age ADHD Case - Vanderbilts.pdfADHD Handout.docxYoung ADHD and Behavior Case - Residents.docxYoung ADHD and Behavior Case - Faculty Guide.docxParenting Handout and Resource Sheet.docxBehavioral and Mental Health Curriculum Survey.docxBehavioral and Mental Health Pre-Post Test.docx [file mep_2374-8265.11400-s001.zip › M. School-age ADHD Case - Vanderbilts.pdf]

D3 NICHQ Vanderbilt Assessment Scale—PARENT Informant

Today's Date: Nov 13 Child's Name: Maria Date of Birth: ________

Parent's Name: Mom Parent's Phone Number: ________

Directions: Each rating should be considered in the context of what is appropriate for the age of your child. When completing this form, please think about your child's behaviors in the past 6 months.

Is this evaluation based on a time when the child ☐ **was on medication** ☒ **was not on medication** ☐ **not sure?**

Symptoms	Never	Occasionally	Often	Very Often
1. Does not pay attention to details or makes careless mistakes with, for example, homework	0	1	2	**3**
2. Has difficulty keeping attention to what needs to be done	0	1	2	**3**
3. Does not seem to listen when spoken to directly	0	1	**2**	3
4. Does not follow through when given directions and fails to finish activities (not due to refusal or failure to understand)	0	1	2	**3**
5. Has difficulty organizing tasks and activities	0	1	2	**3**
6. Avoids, dislikes, or does not want to start tasks that require ongoing mental effort	0	**1**	2	3
7. Loses things necessary for tasks or activities (toys, assignments, pencils, or books)	0	1	**2**	3
8. Is easily distracted by noises or other stimuli	0	**1**	2	3
9. Is forgetful in daily activities	0	1	**2**	3
10. Fidgets with hands or feet or squirms in seat	0	**1**	2	3
11. Leaves seat when remaining seated is expected	0	**1**	2	3
12. Runs about or climbs too much when remaining seated is expected	0	**1**	2	3
13. Has difficulty playing or beginning quiet play activities	0	1	**2**	3
14. Is "on the go" or often acts as if "driven by a motor"	**0**	1	2	3
15. Talks too much	0	1	**2**	3
16. Blurts out answers before questions have been completed	0	1	**2**	3
17. Has difficulty waiting his or her turn	0	**1**	2	3
18. Interrupts or intrudes in on others' conversations and/or activities	0	**1**	2	3
19. Argues with adults	**0**	1	2	3
20. Loses temper	**0**	1	2	3
21. Actively defies or refuses to go along with adults' requests or rules	0	**1**	2	3
22. Deliberately annoys people	**0**	1	2	3
23. Blames others for his or her mistakes or misbehaviors	**0**	1	2	3
24. Is touchy or easily annoyed by others	**0**	1	2	3
25. Is angry or resentful	**0**	1	2	3
26. Is spiteful and wants to get even	**0**	1	2	3
27. Bullies, threatens, or intimidates others	**0**	1	2	3
28. Starts physical fights	**0**	1	2	3
29. Lies to get out of trouble or to avoid obligations (ie, "cons" others)	**0**	1	2	3
30. Is truant from school (skips school) without permission	**0**	1	2	3
31. Is physically cruel to people	**0**	1	2	3
32. Has stolen things that have value	**0**	1	2	3

The information contained in this publication should not be used as a substitute for the medical care and advice of your pediatrician. There may be variations in treatment that your pediatrician may recommend based on individual facts and circumstances.

Copyright ©2002 American Academy of Pediatrics and National Initiative for Children's Healthcare Quality

Adapted from the Vanderbilt Rating Scales developed by Mark L. Wolraich, MD.

Revised - 1102

American Academy of Pediatrics

DEDICATED TO THE HEALTH OF ALL CHILDREN™

NICHQ

National Initiative for Children's Healthcare Quality

McNeil

Consumer & Specialty Pharmaceuticals

Today's Date: ________ Child's Name: ____________________ Date of Birth: ________

Parent's Name: Mom ____________________ Parent's Phone Number: ________

Symptoms (continued)	Never	Occasionally	Often	Very Often
33. Deliberately destroys others' property	**0**	1	2	3
34. Has used a weapon that can cause serious harm (bat, knife, brick, gun)	**0**	1	2	3
35. Is physically cruel to animals	**0**	1	2	3
36. Has deliberately set fires to cause damage	**0**	1	2	3
37. Has broken into someone else's home, business, or car	**0**	1	2	3
38. Has stayed out at night without permission	**0**	1	2	3
39. Has run away from home overnight	**0**	1	2	3
40. Has forced someone into sexual activity	**0**	1	2	3
41. Is fearful, anxious, or worried	0	**1**	2	3
42. Is afraid to try new things for fear of making mistakes	0	1	**2**	3
43. Feels worthless or inferior	**0**	1	2	3
44. Blames self for problems, feels guilty	**0**	1	2	3
45. Feels lonely, unwanted, or unloved; complains that "no one loves him or her"	**0**	1	2	3
46. Is sad, unhappy, or depressed	**0**	1	2	3
47. Is self-conscious or easily embarrassed	0	**1**	2	3

Performance	Excellent	Above Average	Average	Somewhat of a Problem	Problematic
48. Overall school performance	1	2	3	**4**	5
49. Reading	1	2	3	**4**	5
50. Writing	1	2	3	**4**	5
51. Mathematics	1	2	**3**	4	5
52. Relationship with parents	1	2	**3**	4	5
53. Relationship with siblings	1	2	**3**	4	5
54. Relationship with peers	1	2	3	**4**	5
55. Participation in organized activities (eg, teams)	1	2	**3**	4	5

Comments:

For Office Use Only

Total number of questions scored 2 or 3 in questions 1–9: ________

Total number of questions scored 2 or 3 in questions 10–18: ________

Total Symptom Score for questions 1–18: ________

Total number of questions scored 2 or 3 in questions 19–26: ________

Total number of questions scored 2 or 3 in questions 27–40: ________

Total number of questions scored 2 or 3 in questions 41–47: ________

Total number of questions scored 4 or 5 in questions 48–55: ________

Average Performance Score: ________

D3 NICHQ Vanderbilt Assessment Scale—PARENT Informant

Today's Date: 11/13 Child's Name: MARIA Date of Birth: ________

Parent's Name: DAD Parent's Phone Number: ________

Directions: Each rating should be considered in the context of what is appropriate for the age of your child. When completing this form, please think about your child's behaviors in the past 6 months.

Is this evaluation based on a time when the child ☐ **was on medication** ☒ **was not on medication** ☐ **not sure?**

Symptoms	Never	Occasionally	Often	Very Often
1. Does not pay attention to details or makes careless mistakes with, for example, homework	0	1	**2**	3
2. Has difficulty keeping attention to what needs to be done	0	1	**2**	3
3. Does not seem to listen when spoken to directly	0	**1**	2	3
4. Does not follow through when given directions and fails to finish activities (not due to refusal or failure to understand)	0	1	**2**	3
5. Has difficulty organizing tasks and activities	0	1	**2**	3
6. Avoids, dislikes, or does not want to start tasks that require ongoing mental effort	0	**1**	2	3
7. Loses things necessary for tasks or activities (toys, assignments, pencils, or books)	0	**1**	2	3
8. Is easily distracted by noises or other stimuli	0	1	**2**	3
9. Is forgetful in daily activities	0	**1**	2	3
10. Fidgets with hands or feet or squirms in seat	**0**	1	2	3
11. Leaves seat when remaining seated is expected	**0**	1	2	3
12. Runs about or climbs too much when remaining seated is expected	**0**	1	2	3
13. Has difficulty playing or beginning quiet play activities	0	1	**2**	3
14. Is "on the go" or often acts as if "driven by a motor"	**0**	1	2	3
15. Talks too much	0	**1**	2	3
16. Blurts out answers before questions have been completed	**0**	1	2	3
17. Has difficulty waiting his or her turn	0	1	**2**	3
18. Interrupts or intrudes in on others' conversations and/or activities	**0**	1	2	3
19. Argues with adults	0	**1**	2	3
20. Loses temper	**0**	1	2	3
21. Actively defies or refuses to go along with adults' requests or rules	**0**	1	2	3
22. Deliberately annoys people	**0**	1	2	3
23. Blames others for his or her mistakes or misbehaviors	**0**	1	2	3
24. Is touchy or easily annoyed by others	**0**	1	2	3
25. Is angry or resentful	**0**	1	2	3
26. Is spiteful and wants to get even	**0**	1	2	3
27. Bullies, threatens, or intimidates others	**0**	1	2	3
28. Starts physical fights	**0**	1	2	3
29. Lies to get out of trouble or to avoid obligations (ie, "cons" others)	**0**	1	2	3
30. Is truant from school (skips school) without permission	**0**	1	2	3
31. Is physically cruel to people	**0**	1	2	3
32. Has stolen things that have value	**0**	1	2	3

The information contained in this publication should not be used as a substitute for the medical care and advice of your pediatrician. There may be variations in treatment that your pediatrician may recommend based on individual facts and circumstances.

Copyright ©2002 American Academy of Pediatrics and National Initiative for Children's Healthcare Quality

Adapted from the Vanderbilt Rating Scales developed by Mark L. Wolraich, MD.

Revised - 1102

Today's Date: ____________ Child's Name: ______________________________ Date of Birth: ____________

Parent's Name: DAD ______________________________ Parent's Phone Number: ____________

Symptoms (continued)	Never	Occasionally	Often	Very Often
33. Deliberately destroys others' property	0	1	2	3
34. Has used a weapon that can cause serious harm (bat, knife, brick, gun)	0	1	2	3
35. Is physically cruel to animals	0	1	2	3
36. Has deliberately set fires to cause damage	0	1	2	3
37. Has broken into someone else's home, business, or car	0	1	2	3
38. Has stayed out at night without permission	0	1	2	3
39. Has run away from home overnight	0	1	2	3
40. Has forced someone into sexual activity	0	1	2	3
41. Is fearful, anxious, or worried	0	1	2	3
42. Is afraid to try new things for fear of making mistakes	0	1	2	3
43. Feels worthless or inferior	0	1	2	3
44. Blames self for problems, feels guilty	0	1	2	3
45. Feels lonely, unwanted, or unloved; complains that "no one loves him or her"	0	1	2	3
46. Is sad, unhappy, or depressed	0	1	2	3
47. Is self-conscious or easily embarrassed	0	1	2	3

Performance	Excellent	Above Average	Average	Somewhat of a Problem	Problematic
48. Overall school performance	1	2	3	4	5
49. Reading	1	2	3	4	5
50. Writing	1	2	3	4	5
51. Mathematics	1	2	3	4	5
52. Relationship with parents	1	2	3	4	5
53. Relationship with siblings	1	2	3	4	5
54. Relationship with peers	1	2	3	4	5
55. Participation in organized activities (eg, teams)	1	2	3	4	5

Comments:

For Office Use Only

Total number of questions scored 2 or 3 in questions 1–9: ____________

Total number of questions scored 2 or 3 in questions 10–18: ____________

Total Symptom Score for questions 1–18: ____________

Total number of questions scored 2 or 3 in questions 19–26: ____________

Total number of questions scored 2 or 3 in questions 27–40: ____________

Total number of questions scored 2 or 3 in questions 41–47: ____________

Total number of questions scored 4 or 5 in questions 48–55: ____________

Average Performance Score: ____________

American Academy of Pediatrics

DEDICATED TO THE HEALTH OF ALL CHILDREN™

NICHQ

National Initiative for Children's Healthcare Quality

McNeil

Consumer & Specialty Pharmaceuticals

11-19/rev1102

D4 NICHQ Vanderbilt Assessment Scale—TEACHER Informant

Teacher's Name: Mrs. Jones Class Time: ________ Class Name/Period: 1 G

Today's Date: 11/15 Child's Name: Maria Grade Level: 1st

Directions: Each rating should be considered in the context of what is appropriate for the age of the child you are rating and should reflect that child's behavior since the beginning of the school year. Please indicate the number of weeks or months you have been able to evaluate the behaviors: 3.

Is this evaluation based on a time when the child ☐ **was on medication** ☒ **was not on medication** ☐ **not sure?**

Symptoms	Never	Occasionally	Often	Very Often
1. Fails to give attention to details or makes careless mistakes in schoolwork	0	1	(2)	3
2. Has difficulty sustaining attention to tasks or activities	0	1	(2)	3
3. Does not seem to listen when spoken to directly	0	1	(2)	3
4. Does not follow through on instructions and fails to finish schoolwork (not due to oppositional behavior or failure to understand)	0	1	2	(3)
5. Has difficulty organizing tasks and activities	0	1	2	(3)
6. Avoids, dislikes, or is reluctant to engage in tasks that require sustained mental effort	0	(1)	2	3
7. Loses things necessary for tasks or activities (school assignments, pencils, or books)	0	1	(2)	3
8. Is easily distracted by extraneous stimuli	0	1	2	(3)
9. Is forgetful in daily activities	0	1	(2)	3
10. Fidgets with hands or feet or squirms in seat	0	1	(2)	3
11. Leaves seat in classroom or in other situations in which remaining seated is expected	(0)	1	2	3
12. Runs about or climbs excessively in situations in which remaining seated is expected	(0)	1	2	3
13. Has difficulty playing or engaging in leisure activities quietly	(0)	1	2	3
14. Is "on the go" or often acts as if "driven by a motor"	(0)	(1)	2	3
15. Talks excessively	0	(1)	2	3
16. Blurts out answers before questions have been completed	(0)	1	2	3
17. Has difficulty waiting in line	0	1	(2)	3
18. Interrupts or intrudes on others (eg, butts into conversations/games)	0	(1)	2	3
19. Loses temper	(0)	1	2	3
20. Actively defies or refuses to comply with adult's requests or rules	(0)	1	2	3
21. Is angry or resentful	(0)	1	2	3
22. Is spiteful and vindictive	(0)	1	2	3
23. Bullies, threatens, or intimidates others	(0)	1	2	3
24. Initiates physical fights	(0)	1	2	3
25. Lies to obtain goods for favors or to avoid obligations (eg, "cons" others)	(0)	1	2	3
26. Is physically cruel to people	(0)	1	2	3
27. Has stolen items of nontrivial value	(0)	1	2	3
28. Deliberately destroys others' property	(0)	1	2	3
29. Is fearful, anxious, or worried	(0)	1	2	3
30. Is self-conscious or easily embarrassed	0	(1)	2	3
31. Is afraid to try new things for fear of making mistakes	0	(1)	2	3

The recommendations in this publication do not indicate an exclusive course of treatment or serve as a standard of medical care. Variations, taking into account individual circumstances, may be appropriate.

Copyright ©2002 American Academy of Pediatrics and National Initiative for Children's Healthcare Quality

Adapted from the Vanderbilt Rating Scales developed by Mark L. Wolraich, MD.

Revised - 0303

American Academy of Pediatrics

DEDICATED TO THE HEALTH OF ALL CHILDREN™

NICHQ

National Initiative for Children's Healthcare Quality

McNeil

Consumer & Specialty Pharmaceuticals

Teacher's Name: Mrs. Jones Class Time: ______ Class Name/Period: ______

Today's Date: ______ Child's Name: ______ Grade Level: ______

Symptoms (continued)	Never	Occasionally	Often	Very Often
32. Feels worthless or inferior	**0**	1	2	3
33. Blames self for problems; feels guilty	**0**	1	2	3
34. Feels lonely, unwanted, or unloved; complains that "no one loves him or her"	**0**	1	2	3
35. Is sad, unhappy, or depressed	**0**	1	2	3

Performance<br>*Academic Performance*	Excellent	Above Average	Average	Somewhat of a Problem	Problematic
36. Reading	1	2	3	4	**5**
37. Mathematics	1	2	**3**	4	5
38. Written expression	1	2	3	**4**	5

Classroom Behavioral Performance	Excellent	Above Average	Average	Somewhat of a Problem	Problematic
39. Relationship with peers	1	**2**	3	4	5
40. Following directions	1	2	3	**4**	5
41. Disrupting class	1	2	**3**	4	5
42. Assignment completion	1	2	3	**4**	5
43. Organizational skills	1	2	3	**4**	5

Comments:

Please return this form to: ______

Mailing address: ______

______

Fax number: ______

For Office Use Only

Total number of questions scored 2 or 3 in questions 1–9: ______

Total number of questions scored 2 or 3 in questions 10–18: ______

Total Symptom Score for questions 1–18: ______

Total number of questions scored 2 or 3 in questions 19–28: ______

Total number of questions scored 2 or 3 in questions 29–35: ______

Total number of questions scored 4 or 5 in questions 36–43: ______

Average Performance Score: ______

American Academy of Pediatrics

DEDICATED TO THE HEALTH OF ALL CHILDREN®

11-20/rev0303

NICHQ National Initiative for Children's Healthcare Quality

McNeil Consumer & Specialty Pharmaceuticals
