# Supplementary material for: A Case-Based, Longitudinal Curriculum in Pediatric Behavioral and Mental Health
Source: MedEdPORTAL. 2024 Apr 29;20:11400. doi: 10.15766/mep_2374-8265.11400 (PMC11056487; doi:10.15766/mep_2374-8265.11400)
Supplement: Supplementary file 1 — Preteen Anxiety Case - Residents.docxPreteen Anxiety Case - Faculty Guide.docxPreteen Anxiety Case - SCARED Forms.pdfAnxiety Resources Handout.docxASD Delays Case - Residents.docxASD Delays Case - Faculty Guide.docxAutism Summary Handout and Resources.docxDepression Case - Residents.docxDepression Case - Faculty Guide.docxDepression Resources Handout.docxSchool-age ADHD Case - Residents.docxSchool-age ADHD Case - Faculty Guide.docxSchool-age ADHD Case - Vanderbilts.pdfADHD Handout.docxYoung ADHD and Behavior Case - Residents.docxYoung ADHD and Behavior Case - Faculty Guide.docxParenting Handout and Resource Sheet.docxBehavioral and Mental Health Curriculum Survey.docxBehavioral and Mental Health Pre-Post Test.docx [file mep_2374-8265.11400-s001.zip › S. Behavioral and Mental Health Pre-Post Test.docx]

Behavioral and Mental Health Pre- and Post-Test

AUTISM QUESTIONS:

1. At which of the following ages does the AAP recommend that an M-CHAT be administered as part of routine screening for autism?
   1. 12 months
   2. 18 months
   3. 30 months
   4. 36 months
2. You administer an M-CHAT to a toddler as part of his wellness check, and he scores a 5. What would be the most appropriate next step?
   1. Administer M-CHAT follow-up questions
   2. Bring back to clinic in 1-2 months
   3. Immediately refer to Developmental-Behavioral Pediatrics for an autism evaluation
   4. No further evaluation is necessary unless developmental surveillance indicates risk for ASD
3. You administer an M-CHAT to a toddler as part of his wellness check, and he scores a 1. What would be the most appropriate next step?
   1. Administer M-CHAT follow-up questions
   2. Bring back to clinic in 1-2 months
   3. Immediately refer to Developmental-Behavioral Pediatrics for an autism evaluation
   4. No further evaluation is necessary unless developmental surveillance indicates risk for ASD
4. Which of the following is the genetic syndrome most commonly associated with autism spectrum disorder?
   1. Angelman Syndrome
   2. Neurofibromatosis Type 1
   3. Rett Syndrome
   4. Fragile X Syndrome
5. Which of the following is a true statement regarding autism spectrum disorders?
   1. The incidence has seen a decrease in the past 10 years
   2. Rates of autism are higher in boys than in girls
   3. Children should be routinely screened with an M-CHAT R/F at 18 and 36 months of age
   4. Neuroimaging should be performed in all new ASD diagnoses
6. Which of the following statements about the diagnosis of autism spectrum disorder is true?
   1. To be diagnosed with autism, a child must be deficient in social-emotional development AND display some degree of impairing restrictive or repetitive behaviors
   2. Significant deficits in social and emotional reciprocity alone are sufficient for a diagnosis of autism
   3. Significantly impairing restrictive or repetitive behaviors (such as sensory differences or repetitive speech) alone are sufficient for a diagnosis of autism
   4. A child must be older than 24 months to be diagnosed with autism and to thus qualify for services
7. Which of the following statements regarding autism spectrum disorder is NOT true?
   1. The prevalence of autism has increased steadily, and now is about 1 in 54 children
   2. Children with autism spectrum disorder often have deficits in joint attention, which includes social interactions such as pointing and directing gaze
   3. Most individuals diagnosed with autism have similar symptoms and severity levels, and most will respond to similar treatment plans
   4. Most children with autism are diagnosed after the age of 4
8. Which of the following statements regarding the prevalence of autism is true?
   1. According to the CDC’s most recent report, about 1 in 90 girls is identified with autism
   2. About one-third of children with autism have an intellectual disability
   3. Boys are twice as likely to be diagnosed with autism than girls
   4. Minority groups tend to be diagnosed with autism earlier and more often
9. You are seeing a 4-year-old boy with autism spectrum disorder for a follow-up. He currently receives medically-based speech therapy and occupational-therapy, but he does not receive any other services or therapies. Which of the following is an example of a treatment modality that you would consider recommending to this family as part of their son’s treatment plan?
   1. A customized, delayed immunization schedule
   2. Applied behavior analysis (ABA) therapy
   3. Chelation therapy to remove mercury from the body
   4. All of the above
10. As part of a routine wellness check of a 2-year-old girl, you notice that she is significantly delayed in her language and social-emotional skills on her ASQ. Her parents have some concerns about autism, as they have a nephew who has the diagnosis. After your developmental surveillance is complete, you would like to pursue further evaluation. Which of the following should be done right now as part of the initial evaluation process?
    1. Genetic testing with a microarray given the family history of autism
    2. Referral to pediatric neurology to determine if brain imaging is necessary
    3. Referral to audiology for a hearing assessment
    4. Direct to early intervention only; plan to re-evaluate her in clinic in 6-12 months
11. You are seeing a 24-month-old boy in your clinic. The boy is generally healthy, but he does not regularly respond to his name and he seems to generally keep to himself. He only says about 5 words, and sometimes he uses these words in a nonspecific manner. When you try to play with him, his engagement and eye contact are inconsistent, but not absent (for example, he briefly looks at you and smiles when you blow bubbles). His mother reports that he is her only child and that she is not worried because both she and the boy’s father were “late talkers.” Please indicate what you may consider doing next in this child’s evaluation:
12. A 12-month old child presents for his wellness check. Based on your developmental screening and surveillance, he appears delayed in his receptive language, expressive language, and social-emotional skills. His problem-solving and motor skills are at or near age-appropriate levels. What would be an appropriate next step in management?
13. You are concerned about a toddler that you are seeing in your office, even though the M-CHAT and ASQ were within normal limits. What are some examples of play tools/activities you could use in your office to try to elicit normal (or abnormal) social interactions, and thus help you determine plans for further evaluation and management?
14. List 4 symptoms commonly seen in children diagnosed with an autism spectrum disorder.
15. True or False: A family that has one autistic child is not at increased risk over the general population of their future children having autism.
16. True or False: Asperger Syndrome, or “high-functioning autism,” is a diagnosable condition in DSM-5 and can describe individuals with autistic symptoms that have less impairing language and cognitive deficits than others on the autism spectrum.

ADHD QUESTIONS:

1. Which of the following is an example of an amphetamine-class stimulant?
   1. Concerta
   2. Metadate CD
   3. Ritalin
   4. Adderall
2. Which of the following is a common side effect of stimulant medications?
   1. Sedation/drowsiness
   2. Weight gain
   3. Decreased appetite
   4. Dry mouth
3. Which of the following is true about Concerta?
   1. It is an amphetamine-class stimulant
   2. It is a time-release tablet and thus cannot be cut or sprinkled
   3. It is short-acting and is often dosed more than once daily
   4. Constipation is a major and often limiting side-effect
4. Which of the following is true about stimulant medications?
   1. Stimulants are metabolized quickly and thus do not need to be weaned slowly if discontinuing
   2. Stimulants can take 3-6 weeks to reach steady state in the bloodstream
   3. Stimulants tend to act on serotonin receptors in the brain
   4. Stimulants are typically only useful in patients with the hyperactive/impulsive or combined types of ADHD
5. Which of the following is true regarding the diagnosis of ADHD?
   1. ADHD is more common in females than in males
   2. ADHD cannot be diagnosed in children younger than 6 years
   3. Vanderbilt screeners are helpful, but not necessary, for diagnosing ADHD
   4. To be diagnosed with ADHD, a child must have symptoms of both inattention and hyperactivity/impulsivity
6. Which of the following is true regarding ADHD management and prognosis?
   1. Most children with ADHD diagnosed in childhood “grow out” of it by early adolescence, with or without treatment
   2. Risks of not treating ADHD include increased risk-taking behaviors, school underperformance or failure, and decreased self-esteem
   3. Most individuals with ADHD are diagnosed after the age of 12
   4. Most individuals with ADHD diagnosed in early childhood require stimulants to function though adulthood
7. Which of the following medications is FDA approved for treatment of ADHD in children under the age of 6?
   1. Ritalin
   2. Intuniv
   3. Adderall
   4. Concerta
8. All of the above are true about stimulant medications EXCEPT:
   1. They are available in multiple formulations, including tablets, capsules that can be sprinkled, liquid solutions, and chewables
   2. Sometimes stimulants are used “off-label” to treat ADHD in certain age groups in which they are not approved
   3. Some stimulants can have a duration of effect of 10 or more hours
   4. Methylphenidate stimulants and amphetamine stimulants have different side effects that are unique to each class
9. You are seeing a 4-year-old preschooler who was recently diagnosed with ADHD by a psychologist in the community. When you evaluate him, he is quite hyperactive and very difficult to redirect. You agree with the diagnosis, and his parents wonder what treatment modalities are recommended, if any. What do you recommend as the first-line treatment of this child?
   1. Immediate medication trial with a long-acting stimulant
   2. Behavioral therapy to help the child and his parents manage his behaviors
   3. Trial a non-stimulant such as Strattera as stimulants are not indicated in this age group
   4. No formal treatment modalities are recommended for children this age, follow-up as necessary
10. Which of the following is recommended prior to initiating stimulant therapy?
    1. Screening with questions regarding individual cardiac risk and family history of cardiac conditions
    2. EKG for all patients prior to starting stimulants, regardless of age or risk factors
    3. Cardiology consultation for all children under the age of 6 starting stimulants
    4. A trial of behavioral therapy alone is typically indicated prior to starting stimulants
11. A 7-year-old boy is taking Ritalin LA, 20mg daily, for his ADHD, combined type. This helps him stay focused and attentive for most of the day, but he still has occasional issues with impulsivity. Because he is thin and has had a decreased appetite on this medication, you are considering adding Intuniv as adjunctive therapy with his stimulant. What is the typical recommended starting dose of Intuniv?
    1. 1mg
    2. 2mg
    3. 5mg
    4. 10mg
12. Which of the following is a common side effect of Intuniv?
    1. Diarrhea
    2. Weight loss
    3. Sedation
    4. Photosensitivity
13. Which of the following about Intuniv is true?
    1. It may be cut in half or crushed to be given to children who cannot swallow pills
    2. It has a duration of action of 4-6 hours
    3. It should always be given in the morning because it can cause insomnia
    4. Constipation is a side effect that is occasionally seen
14. Which of the following medications commonly used for ADHD has a black-box warning for an increased risk of suicidal thinking in pediatric patients?
    1. Strattera
    2. Concerta
    3. Adderall
    4. Vyvanse
15. Which of the following is true about Vyvanse (Lisdexamfetamine)?
    1. It is a methylphenidate-class stimulant
    2. It has a duration of action of 3-5 hours
    3. It is a prodrug that is converted to its active form after being absorbed by the body
    4. It is a time-release capsule and thus cannot be cut, opened, or sprinkled
16. You are evaluating a 5-year-old boy due to behavior challenges at home and at school. He is in Kindergarten and his parents are exhausted because they are contacted by his school multiple times per week about how he is “out of control” in the classroom. More specifically, he does not focus on his work, he does not listen well or follow directions, and he is often getting into mischief with others, sometimes even to the point of physically hurting others. His parents report that he is often the same at home: he does not listen well and he will often disobey and defy them. He seems as if he is “run by a motor,” and he is highly active and impulsive; redirecting him is quite the challenge, and he will not keep still to play, eat, or perform other important tasks. You are concerned about ADHD, but you want to know more. List 2 tools that could be helpful for further evaluation.
17. You are seeing a 10-year-old boy in your clinic who has ADHD, combined-type. He has been on Concerta for about 2 years now, starting at 18mg and going as high as 36mg, which is his current dose. He has not had many side effects related to this medication, but he has always been on the thin side, with his weight dropping off a little after initiating the medication and being stable at about the 5-10 percentile since then. His appetite is good for breakfast and dinner, but it is much less for lunch, and his parents aren’t sure how much he actually eats when he is at school. Recently, his teacher has been concerned about the quality of his work, and she thinks he is less-focused. His parents agree, and they also report that he seems to be more impulsive and active than previously, which has led to some behavior problems at home. They want to know if there are any other treatment options that could help with his symptoms, while keeping in mind his appetite and weight concerns as noted above. List 3 options (medication or otherwise) that you would consider discussing with his parents.
18. You are seeing a 6-year-old Kindergartener who is struggling with his behavior and performance at school. He also often gets into trouble at home, and he is described as being “defiant” and “incredibly hyperactive.” You suspect that ADHD may be the cause of his symptoms, but you wonder if there are any other explanations. List three alternative explanations that may explain this boy’s clinical presentation.
19. Please list five common side effects of stimulant medications.
20. List 2 common non-stimulant medications that are used to manage ADHD.
21. A 7-year-old girl with ADHD is treated with Adderall XR, 10mg daily. While she is much more focused and less impulsive on this medication, she continues to have difficulties with achievement in school. Her parents wonder if there is anything else going on with her in addition to her ADHD, and they want to know your thoughts. Please list 3 conditions that are commonly co-morbid with ADHD.
22. True or False: The mechanism of action of stimulant medications is that they promote the release of dopamine and norepinephrine in presynaptic nerve terminals in the prefrontal cortex of the brain.

GENERAL DEVELOPMENT QUESTIONS:

1. At which of the following wellness checks does the AAP, per its guidelines, recommend that an ASQ be administered?
   1. 12 months
   2. 18 months
   3. 30 months
   4. 36 months
2. You are seeing a 24-month-old child for a wellness check. You notice that this child does not seem to vocalize much, and you decide to ask his parents more about his language development. Which of the following describes what developmental findings you might expect to see from a child this age?
   1. Speaks at least 200 words
   2. Language is 75% understandable
   3. Starts putting 2 words together to make short sentences
   4. Can use pronouns correctly
3. You are seeing an 18-month-old boy for a wellness check. Which of the following developmental findings would be concerning for a child this age?
   1. Not yet walking
   2. Not yet putting two words together to make sentences
   3. Not yet able to copy a circle
   4. Not yet following 2-step commands
4. Which of the following is true regarding services for children with developmental delays?
   1. Children younger than 3 may be evaluated for an Individual Family Service Plan (IFSP) if they are exhibiting developmental delays
   2. An Individualized Education Program (IEP) can be obtained at all ages for children who are developmentally delayed
   3. A child with autism typically does not qualify for an IEP, but may be able to receive other support services
   4. For a child to be evaluated for an IFSP or IEP, a referral needs to be placed by their primary care provider
5. At a 12-month wellness check, you observe that your patient is behind in her expressive language and fine motor skills. After you administer an ASQ, you find that these domains are indeed in the gray or black areas, which signifies concern for developmental delays. She is otherwise healthy, and she is an only child. Which of the following would be a reasonable next step in the evaluation and management of this child?
   1. Referral to developmental pediatrics
   2. Recommend birth-to-three services
   3. Recommend an IEP evaluation through the school district
   4. Recommend physical therapy to help with motor skills
6. A 15-month-old girl is behind in her expressive and receptive language development. She is socially appropriate and playful, and her motor skills appear to be age-appropriate. Her parents are concerned about her language. Upon further evaluation, you learn that she is generally healthy, and that she passed her newborn hearing screen. You also learn that she has not received any therapies or services in the past. Currently, she does not say any words with specific meaning, and she does not consistently follow 1-step commands. Which of the following you be most likely to recommend to these parents with regards to their daughter’s development?
   1. No further action is necessary; follow-up in 3-6 months
   2. Refer to Pediatric Neurology to explore her significant language delay
   3. Refer to Developmental Pediatrics for an autism evaluation
   4. Refer to audiology for a hearing evaluation
7. You are seeing a child at his 24-month wellness check. Based on your developmental surveillance, he appears to be behind in multiple developmental domains. Please describe 3 developmental milestones expected to be achieved by the age of 24 months.
8. You are evaluating a 21-month-old boy in your clinic, and you are immediately alerted to the fact that he is not yet walking. You review his medical record and notice that, to date, no formal evaluation has been done to assess for this. List at least 2 next steps you may consider in the evaluation and treatment of this patient.
9. True or False: At 48 months of age, most typically developing children are 100% understandable with a vast vocabulary, and they are able to copy a circle and square.
10. True or False: At 36 months of age, most typically developing children are able to gallop and skip.
11. True or False: Echolalia can be considered a normal part of language development up until 30 months of age.
12. True or False: Most typically developing children are able to sit unsupported by 4 months of age.

SLEEP QUESTIONS:

1. Which of the following is considered the first-line medicinal treatment for sleep initiation in pediatric patients?
   1. Trazodone
   2. Melatonin
   3. Guanfacine, immediate release
   4. Diphenhydramine
2. Which of the following is true about melatonin?
   1. It is only available as prescribed by a medical professional
   2. The starting dose for children under 5 is 10mg before bed
   3. Melatonin receptors in the brain can become desensitized, and thus some patients on higher doses can become less receptive to melatonin supplements over time
   4. Most melatonin supplements cannot be crushed, cut, chewed, or otherwise altered for them to be effective
3. A 6-year-old boy has significant daytime somnolence, which is contributing to difficulties with learning at school and challenging behaviors both at home and at school. His teacher has brought up concerns about poor focus and possible ADHD. While you cannot rule-out ADHD as a cause of his learning and behavioral challenges, you are concerned about his sleep habits. Which of the following next steps would be most appropriate in the initial management of this child?
   1. Ask if he snores, perform an HEENT exam, and consider evaluation for obstructive sleep apnea (OSA)
   2. Manage symptoms with a trial of extended-release Guanfacine (Intuniv), which may help him fall asleep at night and control his symptoms of ADHD
   3. Consider a trial of Diphenhydramine (Benadryl) to help him with sleep initiation and maintenance, which may help him feel less tired during the day and may help with his learning
   4. Refer to Pediatric Neurology for a full neurologic exam and possible EEG to explain issues with falling asleep and focus
4. Which of the following is true about melatonin?
   1. It can become denatured and thus less active if left in a well-lit environment, so it is recommended that it is stored in a dark place
   2. It has a rare but well-established side effect of suicidal ideation, which should be discussed with all patients before starting it
   3. The chewable form is less bioavailable than the pill form, and should only be used if children cannot reliably swallow pills
   4. It is most effective when dosed two times daily, once in the morning and once in the evening
5. You are considering starting Clonidine to help a pediatric patient with sleep-onset insomnia. Which of the following is true about this medication?
   1. Clonidine increases the amount of REM sleep in patients
   2. Clonidine is an Alpha-2 Antagonist and thus can increase blood pressure at high doses
   3. It can potentially worsen symptoms of ADHD such as impulsivity, so it should be used with caution in this population
   4. Tolerance often develops with this medication, which sometimes leads to dose increases
6. Which of the following is true regarding antihistamine use for insomnia in children?
   1. About 5-10% of people experience activation with antihistamines like Benadryl, which can preclude their use as a sleep aid for these patients
   2. Tolerance rarely develops, and thus they can be easily used for chronic sleep-onset insomnia
   3. Other than drowsiness, side effects include extrapyramidal effects such as unusual movements
   4. Antihistamines not only act on histamine receptor sites in the body, but they also act on melatonin receptors in the suprachiasmatic nucleus in the brain
7. A 3-year-old girl has “always” had problems with sleep. She falls asleep late at night, wakes up multiple times at night, and will not fall asleep unless a parent lies down with her. Because of this, her parents typically will allow her to sleep in their bed with them; this has become problematic as her parents are not getting adequate sleep, but they do not know what else to do. List three recommendations that you might have for them to help with their family’s sleep issues.
8. A 4-year-old child is having difficulty falling asleep at night. Her parents are exhausted, and they are asking for your help. They are especially interested in medicinal management of the sleep issues. Before discussing medications for sleep, what are some questions you would consider asking these parents as part of your evaluation for sleep problems (list at least three)?
9. List three prescription medications that are commonly used for sleep problems in pediatric patients:
10. True or False: The Nonbenzodiazepine receptor agonists (i.e. the “Z-drugs” like Zolpidem, Zaleplon, and Eszopiclone) are approved for use for sleep-onset insomnia in children, and they do not demonstrate any significant side effects.

DEPRESSION AND ANXIETY QUESTIONS:

1. When evaluating a teenager you suspect has depression, what is of highest priority prior to the end of the session?
2. Arrange school nurse visits so that parents can work with the teen on a stepwise collaborative safety plan
3. Begin antidepressant medication in order to achieve early treatment success
4. Evaluate the teen for suicidality so that an initial safety plan can be discussed
5. Refer the teen for psychotherapy as this is the first line treatment for initial episodes of depression
6. Six weeks ago you saw a 15 yo male in your office and began a trial of fluoxetine for an initial presentation and first episode of MDD, single episode, moderate. In the first week after starting fluoxetine 20 mg daily, he had some mild anorexia and general GI discomfort, which had resolved after about one week. He presents to your office for follow-up and his PHQ-9 Modified score is 15. He is doing more around the house and is less isolative, but he continues to endorse low mood. What is the next most appropriate step in management?
7. Switch to sertraline 50 mg daily
8. Switch to venlafaxine extended release 37.5 mg daily
9. Switch to venlafaxine extended release 75 mg daily
10. Increase fluoxetine to 40 mg daily
11. Increase fluoxetine to 60 mg daily
12. You are seeing a 14 yo female in follow-up for her depression, and her PHQ-9 Modified score is 14. You collaborate with her and her mother to include increasing the sertraline to 100 mg daily as a part of her treatment planning. Psychotherapy intakes are limited at present due to the COVID-19 pandemic. While discussing a recent break-up, she is tearful and says it feels like she is never going to find a good guy and so is always going to be alone. She guiltily looks at her mother at this moment, as her parents are currently separated. This is an example of what type of cognitive distortion?
    1. Overgeneralization
    2. Mental filter
    3. Catastrophizing
    4. Disqualifying the positive
    5. All-or-nothing thinking
13. You are seeing a 16 yo female with depression whom you started on her first antidepressant, sertraline, about 6 months ago. She has tolerated a titration of sertraline to 200 mg daily. Her initial PHQ-9 score was 22, and at this dose of sertraline for one month, her PHQ-9 score is now 14. Symptom reports are consistent with these scores. According to the TORDIA trial, what would be the next most appropriate step in management?
14. Augment the sertraline with bupropion extended release
15. Increase the sertraline to 250 mg daily, and discuss the use of this medication above the FDA max
16. Switch to venlafaxine extended release
17. Emphasize the importance of psychotherapy for treatment-resistant depression to the teen/parent
18. Switch to fluoxetine
19. Which of the following depression symptoms may be more responsive to bupropion?
20. Difficulty falling asleep
21. Decreased appetite
22. Anhedonia
23. Negative cognitions
24. The majority of SSRIs are of which FDA Pregnancy Category?
25. A
26. B
27. C
28. D
29. X
30. You are seeing a 16 yo female in follow-up for menorrhagia. She has been overweight for two years. In discussing some of her recent dieting efforts, she says that she recently gave up her diet. She had been on vacation 3 weeks ago and ate more during that time, but when she returned from vacation, she felt overwhelmed, discouraged, and a sense of shame. She hasn’t returned to her diet since return, noting “I was doing good but did a bad job on vacation and gained like 5 pounds, I figured what’s the point?” This is an example of what type of cognitive distortion?
31. Overgeneralization
32. Mental filter
33. Catastrophizing
34. Disqualifying the positive
35. All-or-nothing thinking
36. Which of the following is an example of behavioral activation?
37. Advising a child that their suicidal thoughts need to be disclosed to a parent
38. Providing organization tips for a high school student with ADHD
39. Working through a parent-child conflict in the appointment
40. Encouraging a depressed teen to take a daily walk
41. Increasing a teen’s awareness of their cognitive distortions
42. What is the most common side effect of selective serotonergic reuptake inhibitors?
43. Headache
44. Weight gain
45. Dulled cognition
46. Gastrointestinal upset
47. Fatigue
48. Categorize the following factors related to suicide risk assessment:

___Anxiety/agitation A. Static Risk Factor

___History of hospitalization B. Modifiable Risk Factor

___Family history C. Protective Factor

___Insomnia

___Spiritual/religious beliefs

___Male gender

___Peer recently died by suicide

___Transgender non-acceptance

___Open communication with parents

___Substance use

Refer to the following case for Questions #11 and #12:

A 13-year-old male is seen for a well child evaluation. His mother reports concerns that he will not leave the house. They encourage him to run errands with them and to go outside for walks, but with an increasing frequency over the past 6 months, he has declined to participate in any activity that takes him out of the home. He has been home-schooled for about 2 years and had been doing well, although in recent weeks he identifies significant trouble concentrating on his work. He admits that when he cannot concentrate he thinks about what is happening due to the pandemic and worries about the safety of his family. When asked about the times he would not leave home prior to the pandemic, he identifies that he just does not like to be out. Once he got so worked up as his parents pressed him to leave that he had a panic attack. His mother has caught him awake long after his bedtime, and as a result he often takes naps during the day and complains of being tired. He admits that at the end of the day he tends to question his decisions from the day and feels particularly badly about not doing well in his classes.

1. What is the most likely diagnosis for this teen?
2. Social phobia
3. No disorder
4. Specific phobia
5. Generalized anxiety disorder
6. Panic disorder with agoraphobia
7. What initial step would you take to address his sleep disturbance?
8. Prescribe daytime sleep deprivation
9. Refer for psychotherapy to address underlying causes of sleep disturbance
10. Refer for a sleep study
11. Prescribe clonidine 30-60 minutes before bedtime
12. Which of the following is a reasonable starting regimen when a PRN medication to abort panic or acute anxiety attacks is indicated?
13. Buspirone 5 mg
14. Hydroxyzine 10 mg Q6H
15. Guanfacine 1 mg QHS
16. Lorazepam 0.5 mg daily
17. Melatonin 3 mg Q6H
18. You see a 14-year-old in clinic and begin sertraline 50 mg daily for a diagnosis of major depressive disorder. The teen returns for follow-up and is tolerating the medication. The PHQ-9 score has decreased from 21 to 15, and subjective report is consistent with this. What is an appropriate range of time for the patient to take the current dose prior to increasing the sertraline dose to 100 mg?
19. 1 week
20. 4 weeks
21. 12 weeks
22. There is no expected range of time for dose titration intervals
23. Which laboratory study is NOT routinely considered in an initial evaluation of a child presenting with depression as a symptom?
24. Urine HCG (if female)
25. Complete blood count
26. Thyroid studies
27. Urine drug screen
28. Lipid panel
29. Which of the following is the suicide method associated with the highest lethality?
30. Asphyxiation
31. Cutting major vessels
32. Gun shot wound
33. Overdose
34. Alcohol poisoning
35. You are seeing an 8-year-old for a well child check; the child missed his well check the summer prior due to family stressors and circumstances following a tornado that struck their home that year. His mother is apprehensive about the start of the school year. She reports “he never seems to want to leave my side… I don’t know if it was the tornado or what, but he used to play with other kids or I could leave him with our good friends down the street to go to the store. He has always been shy, but toward the end of the last year, he started to refuse to go to school and would sometimes scream. We cannot even leave him at my parents’ house overnight anymore.” According to the Child/Adolescent Anxiety Multimodal Study (CAMS), what is the next most appropriate step in management?
    1. Refer for an adequate trial of cognitive behavioral psychotherapy (CBT)
    2. Initiate a trial of duloxetine 30 mg daily
    3. Initiate a trial of sertraline 50 mg daily
    4. Initiate a trial of duloxetine 30 mg daily and refer for an adequate trial of CBT
    5. Initiate a trial of sertraline 50 mg daily and refer for an adequate trial of CBT

SPECIAL EDUCATION QUESTIONS:

1. There are 13 potential disability categories recognized under the Individuals with Disability Education Act (IDEA). List 5:
2. ________________________________________
3. ________________________________________
4. ________________________________________
5. ________________________________________
6. ________________________________________
7. Parental consent is required for each of the following support services except:
   1. 504 Rehabilitation Plan
   2. Evaluation Team Report (ETR)
   3. Individual Education Program (IEP)
   4. Individualized Family Services Plan (IFSP)
8. By definition, a child who meets criteria to qualify for a/an ___________ would also technically meet criteria for a/an ___________.
   1. IEP / 504 Plan
   2. 504 Plan / IEP
   3. Response To Intervention (RTI) / IEP
   4. IEP / RTI
9. Briefly explain why a child with a known disability (e.g., ADHD) would not necessarily qualify for an IEP.
10. True/False: If a parent requests a special education evaluation in writing, a school must complete a full evaluation to determine eligibility.
11. A child’s IEP must be reviewed every (how often?)_____________ and an ETR is re-accomplished every (how often?)__________.
12. From consent, schools must complete an initial evaluation within ________ calendar days and conduct an IEP meeting within ________ calendar days of determining eligibility.
    1. 30/30
    2. 30/60
    3. 60/30
    4. 60/60
13. Please match the following:

| 1. Special education and related services that are provided at public experience, meets state standards, are appropriate, and are provided in conformity with an IEP. | 1. Free and Appropriate Public Education |
| --- | --- |
| 1. Specially designed instruction, at no cost to the parents, to meet the unique needs of the child. | 1. Special Education |
| 1. Legal requirement to educate children with disabilities in general education classrooms with children who are not disabled to the maximum extent possible. | 1. Least Restrictive Environment |
| 1. Impairment that substantially affects one or more major life activities. | 1. Disability |
| 1. Special education and related services provided to children until age 3. | 1. Early Intervention |
| 1. Procedural safeguard to resolve disputes between parents and schools. | 1. Mediation |
| 1. The tiered process used to determine if a child has a learning disability; how a child responds to scientific, research-based strategies. | 1. Response to Intervention |
